# Effectiveness of Self-Training With a Web-Based Digital Health Application Versus Physiotherapy in the Treatment of Disorders of the Patella: Randomized Controlled Trial

**DOI:** 10.2196/66463

**Published:** 2025-05-05

**Authors:** Tobias A Mayer, Daniel Koska, Ann-Kathrin Harsch, Christian Maiwald

**Affiliations:** 1 Department of Research Methodology and Data Analysis in Biomechanics Institute of Human Movement Science and Health Chemnitz University of Technology Chemnitz Germany

**Keywords:** M22, disorders of the patella, randomized controlled trial, RCT, DiGA, physiotherapy, digital health application

## Abstract

**Background:**

Disorders of the patella are among the most prevalent knee injuries. While exercise therapy is widely accepted as an effective treatment strategy, the positive effects of conventional exercise therapy under the guidance of a physiotherapist may be offset by inherent limitations, such as difficulties in scheduling appointments or statutory policies restricting the number of training sessions. Home-based exercise interventions using digital health applications (DHAs) may help address some of these limitations.

**Objective:**

This study aimed to assess the efficacy of a 12-week exercise intervention using a web-based DHA for improving knee function and reducing pain in patients with disorders of the patella (*International Classification of Diseases* code M22).

**Methods:**

The outcomes of the DHA intervention group (IG) were compared to a control group (CG) that received conventional physiotherapy covered by statutory health insurance in Germany (SHI-PT). A total of 259 patients with diagnosed disorders of the patella were included in the trial and randomly allocated to IG DHA (n=136, 52.5%) and CG SHI-PT (n=123, 47.5%). Two primary end points were examined: “knee function” (Knee Injury and Osteoarthritis Outcome Score—Activities of Daily Living [KOOS_ADL_] subscale, range 0-100 points) and “knee pain” (visual analog scale [VAS], range 0-100 points). Participants were asked to complete 2 surveys: one before the first therapy session (PRE) and one after completing the treatment period of 12 weeks (POST).

**Results:**

Training with the DHA resulted in a 4.5-fold greater improvement in “knee function” (PRE–POST differences in KOOS_ADL_ score; IG DHA: 15.7 points, 95% CI 13.7-17.6 vs CG SHI-PT: 3.5 points, 95% CI 1.5-5.5) and a 3.5-fold greater reduction in “knee pain” (PRE–POST differences in VAS pain score; IG DHA: –22.5 points, 95% CI –25.2 to –19.9 vs CG SHI-PT: –6.5 points, 95% CI –8.7 to –4.4) compared to SHI-PT. The improvements in IG DHA exceeded the limits of clinical relevance. The differences between the treatment groups (KOOS_ADL_ score –10.1 points, 95% CI –infinity to -8.0; VAS pain score 14.3 points 95% CI 11.7-infinity) were statistically significant (*P*<.001) for both end points in favor of IG DHA. No effect was found for age or sex. The reported use of pain medication decreased substantially in IG DHA, and showed almost no change in CG SHI-PT.

**Conclusions:**

Our findings indicated that the investigated DHA is superior to SHI-PT for treating disorders of the patella. Therefore, DHA has been approved by the German Federal Institute for Drugs and Medical Devices for treating disorders of the patella in persons of all sexes aged ≥12 years.

**Trial Registration:**

German Clinical Trials Register (DRKS) DRKS00023454; https://drks.de/search/en/trial/DRKS00023454

## Introduction

### Background

The knee is the second most common site for musculoskeletal pain [1], with 11% to 17% of the cases affecting the anterior knee or the patellofemoral joint area. Muscular dysfunctions are considered an important biomechanical cause of such patella-related disorders [2-4]. Therefore, exercise therapy is widely regarded as an effective treatment strategy [4-8]. Conventional exercise therapy is typically administered on an outpatient basis under the guidance of a physiotherapist. However, the benefits of having a trained physiotherapist may be offset by drawbacks such as difficulties in scheduling appointments or a limited number of training sessions available due to regulatory limitations of German statutory health insurance. Therefore, home-based exercise interventions may present a valid alternative, as they have been reported to be at least as effective as traditional physiotherapy in reducing patellofemoral pain [9] and improving knee function [10]. Kettunen et al [11,12] found that an 8-week home exercise program alone was as effective as knee arthroscopy combined with an 8-week home exercise program in improving knee function and reducing pain. Similar findings have been reported for other knee conditions, such as meniscal tears [13-15], knee osteoarthritis [16,17], and other nonoperative knee conditions [18]. While unsupervised home-based exercise programs have demonstrated clinical effectiveness, they often lack structured feedback, personalization, and sustained engagement [19]. Frequent barriers to following physiotherapist-prescribed exercise programs also include limited social support, low self-efficacy, and a sense of helplessness [20]. These limitations have led to increased interest in digital health applications (DHAs), which aim to address these gaps by delivering exercise therapy through interactive, software-based tools, such as mobile apps or web-based platforms [21,22]. Compared to conventional physiotherapy, DHAs may also improve accessibility, especially for individuals with limited mobility or scheduling constraints [23,24], and enable more flexible and frequent engagement with exercise programs. Furthermore, they offer scalable, cost-effective solutions that can help alleviate health care resource limitations [25], as well as comprehensive possibilities to visualize exercises, educate about the condition in question, and track training sessions and progress, which may boost individual training motivation. There is evidence that DHAs may achieve better efficacy than conventional home training, for example, in treating degenerative meniscal tears [26], low back pain [27], or shoulder disorders [28]. However, only a few randomized controlled trials (RCTs) have compared DHAs directly to conventional physiotherapy covered by statutory health insurance in Germany (SHI-PT), and none of them have investigated patellar disorders. In Germany, the Digital Healthcare Act allows for DHAs to be covered by statutory health insurance once their efficacy is demonstrated. This encompasses device safety, data protection, as well as demonstrable benefits compared to standard care. Following this, DHAs have undergone rapid development in Germany since 2020. As of March 31, 2025, 40 applications have been permanently approved for prescription, while 19 applications have been preliminarily approved. This includes several DHAs for exercise therapy [29-32] that have been granted approval for coverage by German statutory health insurance.

### Objective

In this context, this study aimed to evaluate the efficacy (improvement in knee function and pain) of a 12-week exercise intervention using a novel DHA (Mawendo) compared to SHI-PT in patients with disorders of the patella (*International Classification of Diseases, Tenth Revision* [*ICD-10*] code M22). The corresponding research question was as follows: is the novel DHA clinically superior to SHI-PT in improving knee function and reducing knee pain for patients with *ICD-10* M22 over a 12-week intervention period?

## Methods

### Ethical Considerations

The prespeciﬁed study protocol was approved and registered by the Institutional Ethics Committee of the Faculty of Behavioural and Social Sciences of the Chemnitz University of Technology (registration number V-439-17-CM-MAWENDO-II-18042021) in agreement with current data protection regulations. The trial was registered with the German Clinical Trials Register (World Health Organization [WHO] Primary Register) under the ID DRKS00023454 for 14 different orthopedic *ICD-10* indications. All participants had to give written informed consent before enrollment in the study, and were allowed to withdraw at any time without incurring any negative consequences. The DHA Mawendo complies with all currently active data protection regulations and laws in Germany and Europe. The participants of the study did not receive any compensation, or supplemental assistance regarding the use of the DHA beyond what is generally provided. Data were deidentified before analysis.

### Study Design

The aforementioned 14 *ICD-10* indications will be examined in 11 separate randomized controlled prospective clinical trials with 2 treatment groups. This RCT is the first among the 11 RCTs to complete the treatment of the prespecified number of patients. At the time of writing this manuscript, the 10 remaining RCTs are still in the stage of data collection. In this work, different strategies for treating diseases of the patella (*ICD-10* M22) were compared between an active control group (CG) that received the current standard therapy in Germany (CG SHI-PT) and an intervention group (IG) using the DHA Mawendo (IG DHA). Participants and investigators were not blinded to the type of treatment or group allocation.

### Study Population

The participants in this study were recruited by orthopedists (recruiters) during their regular consultation activities at orthopedic clinics and medical practices. Patients with confirmed disorders of the patella and internet access were eligible for participation. Children (aged <12 years) were not allowed to take part in the study. Adolescents (aged <18 years) required the consent of a legal representative. The patients were fully informed about the study by the attending orthopedist and received a study information sheet outlining the study’s objectives, procedures, and treatment conditions (DHA vs physiotherapy). The pain intensity level was assessed by the recruiter before the study inclusion using a Verbal Numerical Rating Scale (VNRS, value range 0-10) [33]. Patients with very low (VNRS pain intensity ≤2) or very severe pain (VNRS pain intensity ≥8) were excluded from the study to control for disease severity. The following illnesses or physical conditions were defined as exclusion criteria:

- Knee surgery up to 6 months before the start of treatment
- Severe or acute diseases of the cardiovascular system (eg, acute myocardial infarction, acute ischemic heart disease, high blood pressure with heart failure, or hypertensive crisis)
- Diseases of the lungs or respiratory tract (eg, pneumonia or pulmonary embolism)
- Tumor diseases (eg, malignant neoplasm of the bone and articular cartilage or malignant neoplasm of internal organs)
- Infection and fever (eg, rheumatic fever, purulent arthritis, sepsis, or bacterial infection)
- Injuries or diseases of the musculoskeletal system outside the indication ICD-10 M22
- Bleeding tendencies (history of increased bleeding or taking anticoagulant medication)
- Mental disorders (eg, acute psychosis)
- Severe visual impairment
- Pregnancy

The study data were analyzed according to the intention-to-treat principle. Therefore, the occurrence of an exclusion criterion during the study did not lead to the exclusion of participants from further data analysis. Data on additional patient characteristics, such as BMI, activity level, duration of symptoms, type of patellar disorder, and previous therapies, were not collected, as the primary aim was to evaluate the effectiveness of the DHA in real-world conditions, where broad accessibility is prioritized over detailed subgroup analyses. Collecting only essential data for the primary analysis also helped minimize participant burden. Previous experience with digital devices was not assessed, as the intervention (DHA Mawendo) was designed to be intuitive and accessible regardless of technological familiarity.

### Randomization

Participants were allocated to the treatment arms using simple randomization within the following strata: (1) recruiter; (2) pain medication intake of maximum WHO level 1 (yes or no); and (3) baseline VNRS pain level (3-4 or 5-7)

**Figure 1:**
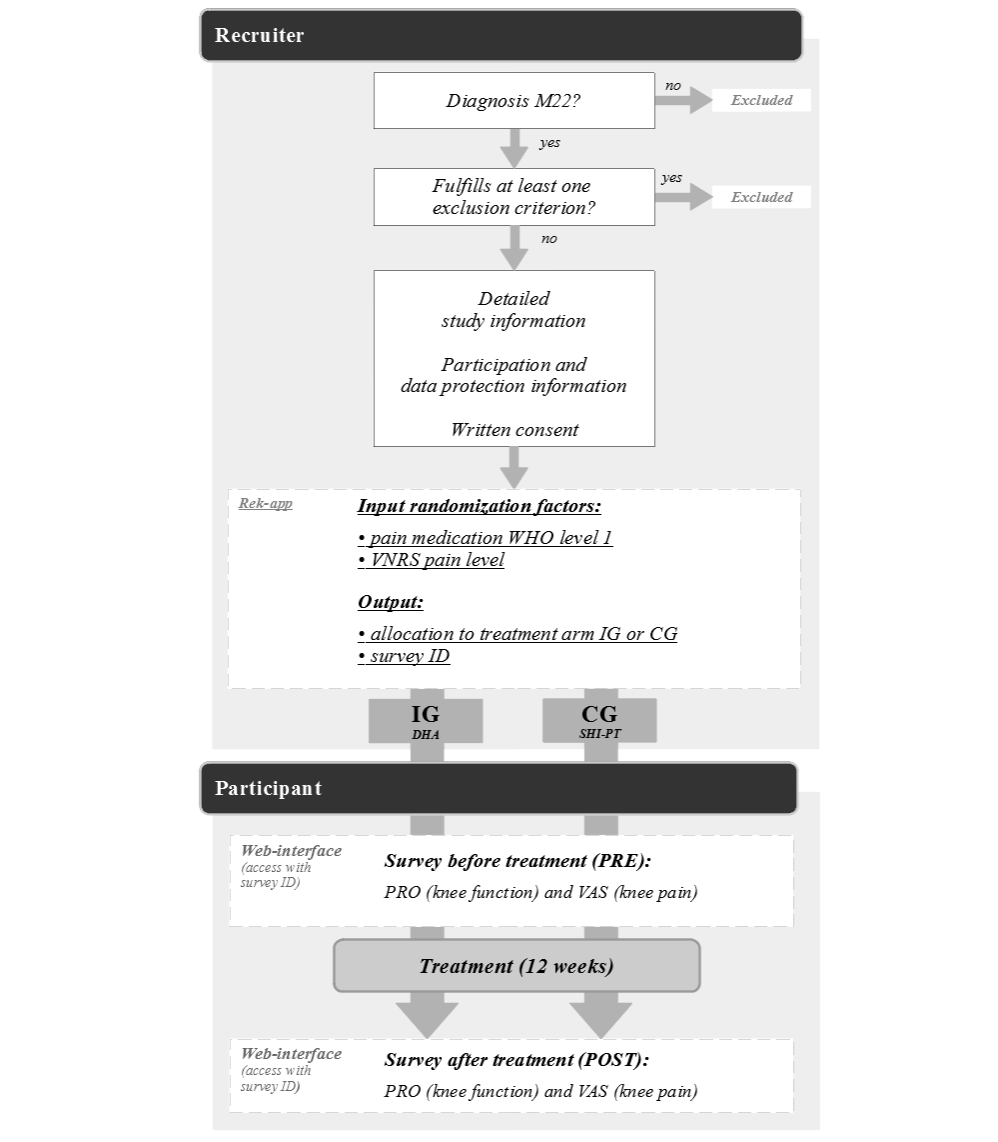
Flowchart of recruitment, randomization, and data collection. CG: control group; DHA: digital health application; IG: intervention group; SHI-PT: physiotherapy covered by statutory health insurance in Germany; VAS: visual analog scale; VNRS: Verbal Numerical Rating Scale; WHO: World Health Organization.

Simple randomization was used to impede the recruiter’s ability to predict the sequence order. Stratification by recruiter was implemented to account for potential variability in patient selection, initial assessments, and treatment initiation timelines across recruitment centers. This approach aimed to enhance internal validity and reduce confounding effects arising from differences in clinical judgment or regional health care structures. Current literature provided no evidence for potential bias between treatment arms for treatment history, stage of disease, age, or sex in previous studies and thus did not include them as strata for randomization. A browser-based web application (Rek-app; Mawendo GmbH) created specifically for recruiting purposes was provided to the recruiters. A set of 4 randomization lists (2 levels for pain medication times 2 levels for baseline VNRS pain) with 100 patients each was stored for each recruiter in the Rek-app. The statistical software R (version 3.6.2; R Foundation for Statistical Computing) was used to create these randomization lists [34]. The recruiters used the Rek-app to check exclusion criteria, include participants in the study, and randomly allocate participants to treatment arms according to the stored randomization lists (allocation sequence was concealed from the recruiters). The Rek-app provided recruiters and participants with a participant-specific ID, through which the participants gained access to the web-based survey platform. A total of 7 recruitment centers were involved with this study. Figure 1 provides a summary of the recruitment process.

### Intervention

The intervention for the active CG SHI-PT consisted of conventional physiotherapy (Germany’s current standard medical care), where participants selected their physiotherapists and made their appointments. IG DHA was treated using DHA Mawendo (version 1.6; certified medical device, risk class 1) and used the DHA at any suitable location.

#### Treatment of CG SHI-PT

- Preparation of an individual treatment plan
- Assistance provided by the physiotherapist
- Execution of physiotherapeutic measures on or with the patient
- Standard treatment duration
- Necessary rest following treatment
- Progress documentation and, if necessary, progress report to the prescribing physician
- Additional work and administrative tasks

Ideally, the first physiotherapy treatment session should include a medical history, pain localization, and, if necessary, mobility tests. The physiotherapist then develops a therapy plan that is implemented in the subsequent sessions. This may include active and passive mobilization and strengthening exercises or manual therapy. The number of sessions and prescriptions within the study period was determined by the treating orthopedist according to the current statutory health regulations in Germany. These permit 6 to 12 sessions during a 12-week treatment period, with up to 18 sessions allowed in severe cases.

#### Treatment of IG DHA

**Figure 2:**
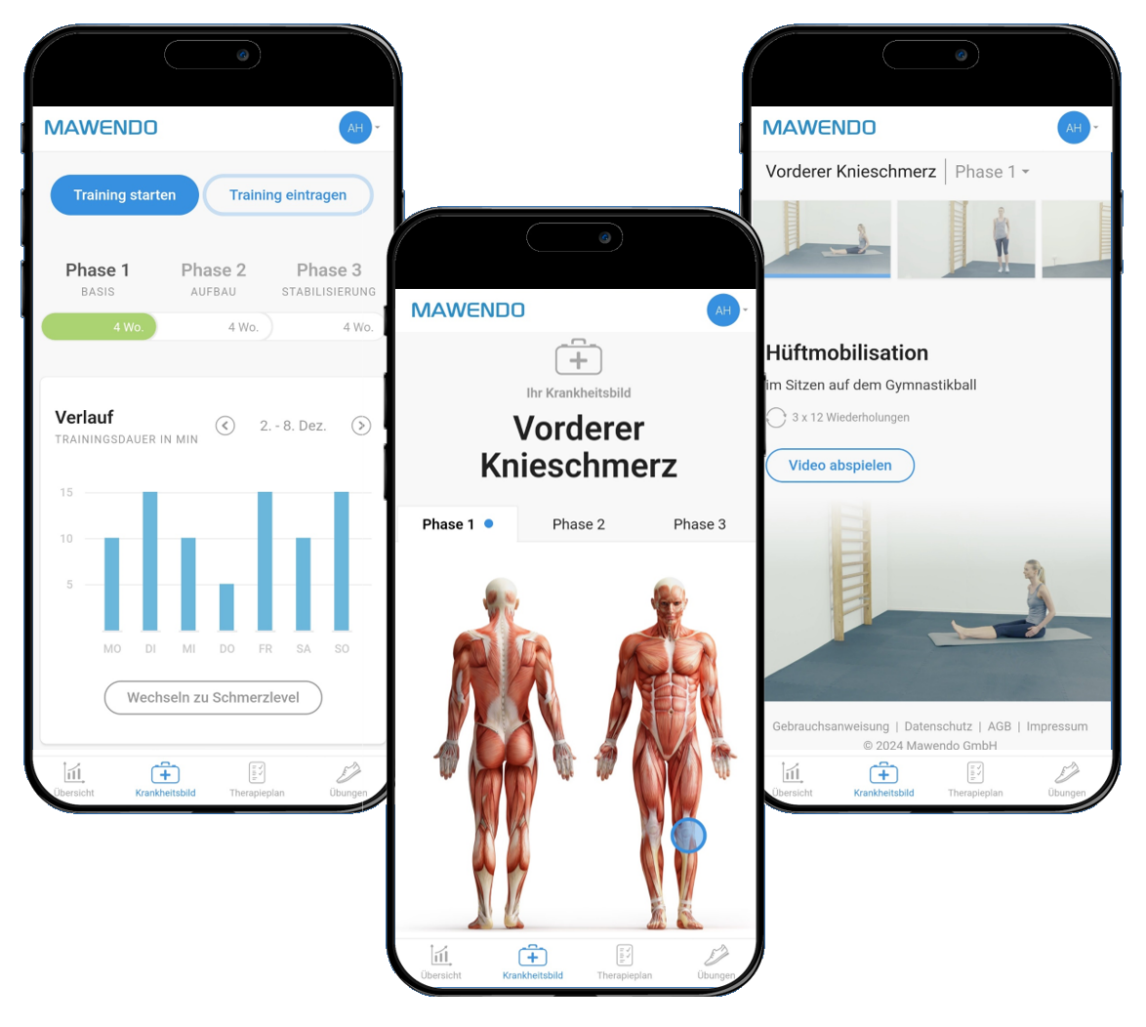
Screenshots of the digital health application Mawendo for International Classification of Diseases code M22.

The DHA used in this study is a browser-based web application that provides digital exercise videos with self-explanatory exercise instructions, health information, and documentation options for self-administered home training (screenshots present in Figure 2). Training sessions using the DHA contain sets of exercises lasting between 20 and 40 minutes, corresponding to the duration of a physiotherapy session. The user interface of the app includes the following sections: (1) overview, (2) education and information, (3) therapy plan, and (4) exercises.

In the overview section, users can record their training frequency and pain levels. Entries are used to feed a progress chart, which is used to track changes over time. The education and information section contains information and educational material regarding the indication being treated. The therapy plan section includes a therapy timeline, which is divided into 3 successive phases: basic, advanced, and stabilization. To ensure training progress and to avoid excessive demands, the 3 phases are designed to provide increasing exercise intensity and difficulty. The default duration of the 3 phases is 4 weeks. However, the prescribing physician may adjust the duration of the phases to align the training program optimally with the patient’s circumstances. In each phase, 8 to 16 exercises are provided (mobilization, coordination, strengthening, stretching, and massage), which should be performed 2 to 3 times a week during the treatment period of 12 weeks. The exercise section) provides instructional videos for the exercises to be performed by participants in their current therapy phase. An overview of available exercises in each phase, including their objectives, is provided in the Multimedia Appendix 1 of this paper. Generally, the exercises for the various medical indications of DHA Mawendo are selected and compiled following the guidelines established by scientific medical societies and other scientific studies [35-54]. A systematic literature review is conducted for each medical indication on a biennial basis, under the supervision of an orthopedic surgeon, to ensure that the content of DHA aligns with the most recent scientific findings. A total of 215 articles were identified as relevant for the compilation of exercises for M22. These articles were then systematically analyzed for practical relevance. The list of articles relevant to the *ICD-10* M22 exercises can be found in the Multimedia Appendix 2. For IG DHA, the treating orthopedist selected the training program in the DHA for the 12-week treatment period, with the option to deselect exercises deemed unsuitable for the patient. Participants could initiate their treatment immediately after recruitment.

### Outcome Measures

#### Overview

Two primary end points were examined in this study: (1) improvement in “knee function” and (2) reduction of “knee pain.”

Both end points describe different aspects of positive treatment effects and were therefore considered equally valid to assess medical benefit. The study was designed to evaluate therapeutic superiority of the DHA over SHI-PT in at least 1 of the 2 end points to comply with regulations given by the Federal Institute for Drugs and Medical Devices in Germany (Bundesinstitut für Arzneimittel und Medizinprodukte [BfArM]).

The end points were assessed using an electronic questionnaire accessible using a web browser and an internet connection. The questionnaire was hosted and operated independently from any IT infrastructure associated with the DHA.

Participants were asked to complete a survey twice during the course of the study: (1) PRE: survey before the first therapy session with DHA or the first physiotherapy treatment and (2) POST: survey after completing the treatment period of 12 weeks.

Participants received the survey URL by email at the specified times and were reminded a maximum of 3 times by email to complete the pending questionnaires.

The end point “knee function” was quantified using Knee Injury and Osteoarthritis Outcome Score (KOOS), the German version of the Patient Reported Outcome [55]. KOOS is a validated questionnaire that measures aspects of health and functional status in diseases and injuries of the knee joint using 5 scales (“Pain,” “Symptoms,” “Activities of Daily Living,” “Functionality in Sports and Leisure,” and “Quality of Life-Related to the Affected Knee”). The KOOS—Activities of Daily Living (KOOS_ADL_) subscale was used to represent “knee function.” The KOOS_ADL_ score is calculated from the 17 “Activities of Daily Living” items, with each item scored from best (0 points) to worst (4 points). A KOOS_ADL_ score of 0 points represents extreme knee problems, and 100 points represents no knee problems (equation 1).

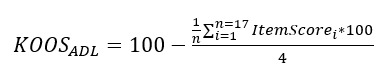



The end point “knee pain” was assessed using a separate pain score based on a visual analog scale (VAS, range 0-100 points, where 100 points represents the worst pain imaginable). Participants were asked to quantify the intensity of their pain over the past week [56].

#### Adherence

In addition to the primary outcome measures, adherence was assessed through self-reported data collected via the web-based survey platform at the end of the intervention period. Participants in both groups were asked to indicate how many of the prescribed therapy sessions they completed, selecting from predefined response categories: “all sessions completed,” “almost all sessions completed,” “more than half completed,” “half completed,” or “fewer than half completed.”

#### Medication

Participants were asked to report whether they were using medication, including pain medication, before (PRE) and after the intervention (POST). However, no further details on medication frequency or dosage were collected. A list of all reported pain medications is included in the Multimedia Appendix 3.

#### Safety Monitoring and Concomitant Care

Safety and adverse events were assessed through self-reported data collected via the web-based survey platform in the POST questionnaire. Participants were specifically asked whether any exclusion criteria had emerged during the treatment phase. These included severe or acute cardiovascular diseases, respiratory disorders, malignancies, infections, fever, musculoskeletal injuries or disorders unrelated to the training program, bleeding tendencies, psychiatric disorders, pregnancy, or other similar physical impairments. In addition, participants reported whether they had undergone surgery related to the diagnosed condition (*ICD-10* M22). However, no information was available regarding whether adverse events were directly attributable to the respective therapy.

Concomitant care was assessed using the PRE and POST questionnaires. Participants were asked whether they used additional therapies, such as acupuncture, and if so, they could list up to 10 additional therapies.

### Sample Size

Due to multiple testing (one hypothesis for each of the 2 primary end points), the significance level was adjusted using the Bonferroni method and set at α=0.0125 (1-tailed testing). The minimal clinically important difference was used as the effect size to be detected, and a power of 1–β=0.9 was specified to calculate the required sample size for a superiority scenario. The minimal clinically important differences for both outcomes were determined from several studies (KOOS_ADL_: 10 points, SD 15 points) [57-59] and VAS: 10 points, SD 22 points [56,60,61]). This resulted in sample sizes of n=56 (KOOS_ADL_) and n=121 (VAS). The more conservative (larger) sample size of n=121 was used to generate sufficient statistical power for both end points. Sample sizes were determined using the SampleSize4ClinicalTrials R package [62].

### Statistical Analysis

#### Overview

Hypothesis tests were performed on null hypotheses of DHA not providing superior medical benefit in either one of the end points compared to SHI-PT.

A linear model (analysis of covariance [ANCOVA]) was used to determine the effectiveness of DHA for both end points. The outcome score in the respective end point (“knee function,” “knee pain”) at POST was used as the dependent variable, the treatment group as the predictor variable, and the PRE score as a covariate. The strata “recruitment center,” “pain medication intake,” and “VNRS pain level” were used as control variables. Type II sum of squares were used due to simple randomization and unbalanced group sizes. For missing values in either PRE or POST the Missing at Random assumption was assumed to be valid. These missing values were imputed using the jump to reference method (J2R, R package RefBasedMI) [63,64]. The J2R method was selected for its ability to mitigate the risk of overestimating the efficacy of the IG. This approach is characterized by its conservatism and robustness in handling missing data in RCTs. For KOOS_ADL_, the imputations were executed at item level and the total KOOS_ADL_ score was calculated for each of 50 imputations (reference group: SHI-PT; covariates: “recruitment center,” “pain medication intake,” and “VNRS pain level”). Implausible values <0 resulting from the J2R imputation were replaced by the value 0, and floating-point numbers were rounded to integers. Variables that were not required for the inferential statistical analysis of the 2 primary clinical end points, such as adherence data or information on additional therapies, were not imputed.

The model assumptions of the ANCOVA models of both outcomes were checked graphically for all 50 imputed datasets and were met sufficiently. The mi.anova function of the R package miceadds [65] was used to calculate the pooled ANCOVA tables. All analyses were performed using the statistical software R (version 4.2.3; R Foundation for Statistical Computing) [66].

#### Nonprespecified Additional Analyses

In addition to the prespecified ANCOVA analysis, exploratory statistical analyses were conducted to further examine potential intervention differences for the categorical factor “sex” and the numerical covariate “age” [67-73]. Apart from that, descriptive analyses were performed to better illustrate the relationship between PRE scores, and treatment success (POST–PRE difference), as well as recruiter-specific effects (statistically significant covariates). To this end, a scatter plot was generated to examine the association between PRE scores and treatment success (POST–PRE). Furthermore, a difference plot was used to illustrate the distribution of treatment success across different recruiters.

## Results

### Participants and Adherence (Self-Reporting)

**Figure 3:**
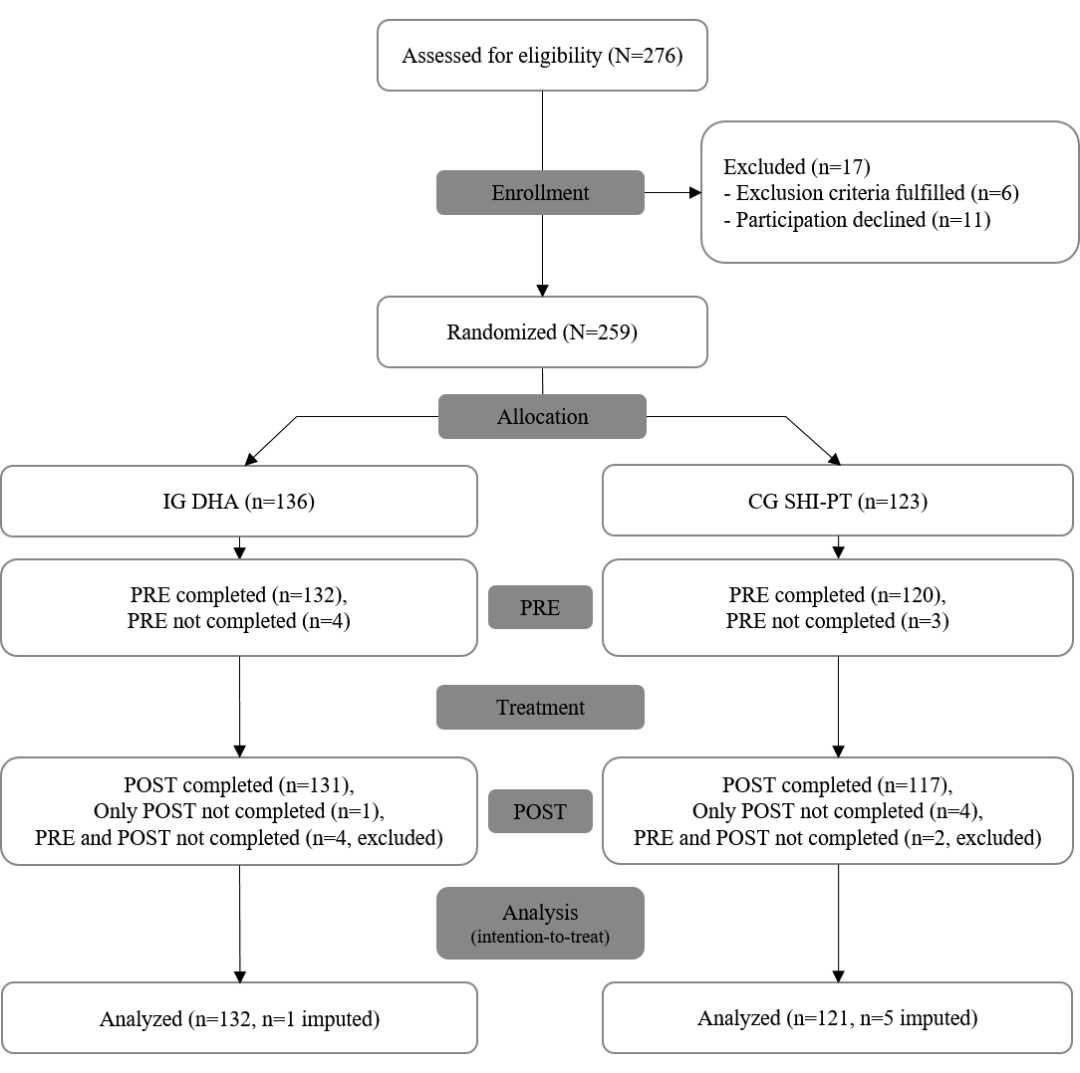
Study flowchart according to CONSORT (Consolidated Standards of Reporting Trials). CG: control group; DHA: digital health application; IG: intervention group; POST: survey after completing the treatment period of 12 weeks; PRE: survey before the first therapy session; SHI-PT: physiotherapy covered by statutory health insurance in Germany.

In total, 259 participants were recruited between November 10, 2021, and January 21, 2023, and randomly assigned to either IG DHA or CG SHI-PT. Among the 259 participants, IG DHA (n=136, 52.5%) received treatment using the DHA, while CG SHI-PT (n=123, 47.5%) received SHI-PT. A total of 6 participants (IG DHA: 4/136, 2.9%; CG SHI-PT: 2/123, 1.6%) provided no survey data despite several email inquiries. These participants were assumed to be missing completely at random and excluded from further analysis. Subsequently, data from 253 participants (IG DHA: n=132, 51%; CG SHI-PT: n=121, 46.7%) were analyzed. Another 6 of the remaining 253 participants provided incomplete datasets (IG DHA: 1/132, 0.8%; CG SHI-PT: 5/121, 4.1%; assumption missing at random): 0.4% (1/253) of participants did not provide PRE data, and 2% (5/253) of participants were lost to follow-up (POST data missing). These missing data were imputed for the inferential statistical analysis [74]. Figure 3 summarizes the aforementioned information in a flowchart according to CONSORT (Consolidated Standards of Reporting Trials) [75]. The demographic and clinical characteristics of IG DHA and CG SHI-PT are listed in Table 1.

In total, 118 (89.4%) out of 132 participants in IG DHA and 103 (85.1%) out of 121 participants in CG SHI-PT reported completing “all” or “almost all” treatment sessions. Additional adherence data can be found in Table 2.

A total of 2 participants (IG DHA: 1/132, 0.8%; CG SHI-PT: 1/121, 0.8%) reported a change in therapy during the course of the study due to time restrictions (IG DHA) and supplementary medication (CG SHI-PT). Of 121 participants, 3 (2.5%) in CG SHI-PT reported the occurrence of ≥1 exclusion criteria during the treatment phase. Of 3 participants, 2 (66.7%) reported an infection during treatment (answer option “infections and fever, eg, rheumatic fever, purulent arthritis, sepsis, bacterial infections”) and 1 (33.3%) participant reported an unspecified mental disorder (answer option “mental disorders, eg, acute psychosis”).

**Table 1:** Demographic and clinical characteristics of IG DHA and CG SHI-PT.

Characteristics			IG DHA^a^ (n=132)		CG SHI-PT^b^ (n=121)
**Sex,** **n (%)**					
	Male	71 (53.8)		74 (61.2)	
	Female	59 (44.7)		44 (36.4)	
	Not specified	2 (1.5)		3 (2.5)	
Age (y), mean (SD)			35.4 (14.4)		36.8 (12.1)
**Pain medication upon enrollment, n (%)**					
	Yes	34 (25.8)		37 (30.6)	
	No	98 (74.2)		84 (69.4)	
**Pain level upon enrollment (range 0-10), n (%)**					
	3-4	78 (59.1)		72 (59.5)	
	5-7	54 (40.9)		49 (40.5)	

^a^IG DHA: intervention group using digital health application.

^b^CG SHI-PT: control group with physiotherapy covered by statutory health insurance in Germany.

**Table 2:** Number of completed treatment sessions.

	IG DHA^a^ (n=132), n (%)	CG SHI-PT^b^ (n=121), n (%)
All	84 (63.6)	94 (77.7)
Almost all	34 (25.8)	9 (7.4)
More than half	12 (9.1)	8 (6.6)
Half	0 (0)	2 (1.7)
Not specified	2 (1.5)	8 (6.6)

^a^IG DHA: intervention group using digital health application.

^b^CG SHI-PT: control group with physiotherapy covered by statutory health insurance in Germany.

### Primary End Point “Knee Function” (KOOS_ADL_ Score)

In IG DHA, the pooled mean KOOS_ADL_ score improved from 66.2 points (95% CI 64.3-68.1 points) to 81.9 points (95% CI 80.3-83.5 points) from PRE to POST. This corresponds to an improvement in “knee function” of 15.7 points (95% CI 13.7-17.6 points).

In CG SHI-PT, “knee function” (pooled mean KOOS_ADL_ score) improved by 3.5 points (95% CI 1.5-5.5 points) from 70.4 points (95% CI 68.3-72.5 points) PRE to 73.9 points (95% CI 71.5-76.3 points) POST.

**Figure 4:**
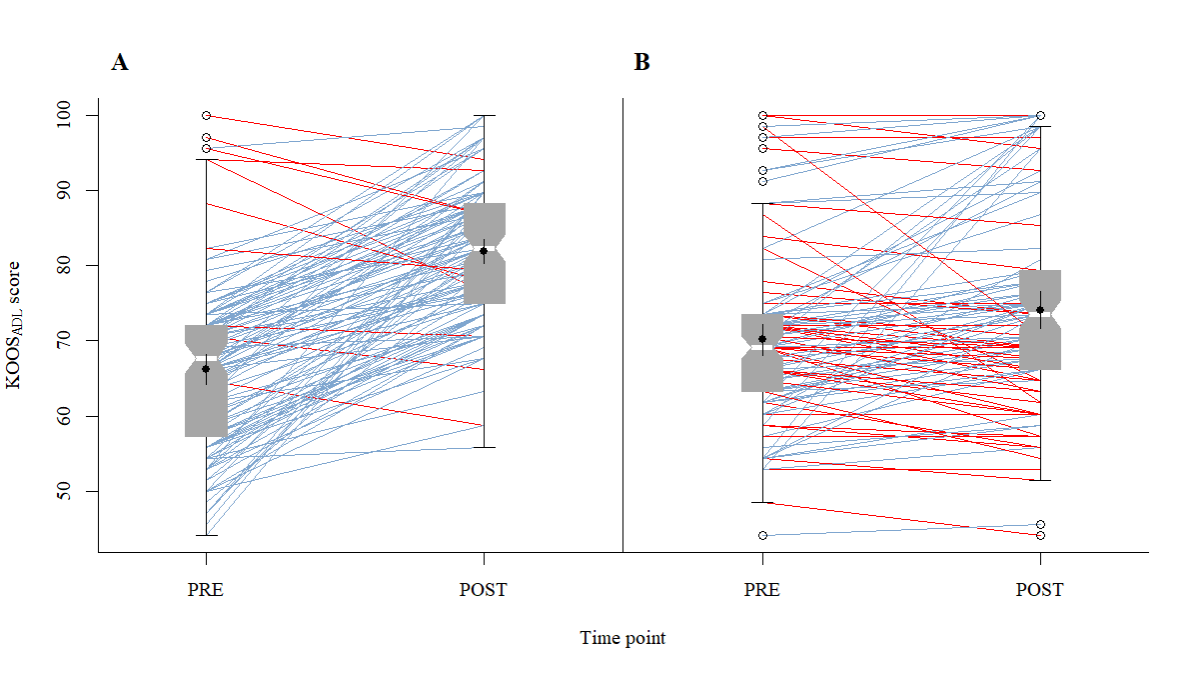
The comparison of Knee Injury and Osteoarthritis Outcome Score—Activities of Daily Living score (KOOS_ADL_) between PRE and POST intervention surveys (A: IG DHA; and B: CG SHI-PT), using the imputed dataset with the largest effect for CG SHI-PT. Each panel displays notched boxplots (95% CI of median), including mean with 95% CI. Intraindividual positive treatment effects are in blue and negative or no effects are in red.

Training with DHA resulted in a 4.5 times greater improvement in “knee function” compared to SHI-PT. Figure 4 illustrates this result using the imputed dataset with the largest effect for CG SHI-PT.

The difference in KOOS_ADL_ score between IG DHA and CG SHI-PT was statistically significant (*P*<.001; Table 3) and estimated at –10.1 points (-infinity to –8.0 points; adjusted 1-sided 95% CI; Table 4) by the pooled ANCOVA (factor “Intervention”).

**Table 3:** Analysis of covariance (ANCOVA) “knee function” (R2=0.389).

	SSQ^a^	*df* 1	*F*	*P* value	*η* ^2^	Partial *η*^2^
KOOS_ADL_^b^ score PRE^c^	6890.216	1	63.479	<.001	0.186	0.234
Intervention	6391.123	1	67.002	<.001	0.173	0.221
Recruiter	983.384	6	1.256	.28	0.027	0.042
Pain medication	74.278	1	0.785	.38	0.002	0.003
Pain level	28.946	1	0.266	.61	0.001	0.001
Residual	22,584.157	—^d^	—	—	—	—

^a^SSQ: sum of squares.

^b^KOOS_ADL_: Knee Injury and Osteoarthritis Outcome Score—Activities of Daily Living.

^c^PRE: survey before the first therapy session.

^d^Not applicable.

**Table 4:** Analysis of covariance (ANCOVA) “knee function” pooled estimates with adjusted 1-sided 95% CI.

Term	Coefficient	SE	95% CI
Intercept	48.512	4.965	40.310 to inf^a^
KOOS_ADL_^b^ score PRE^c^	0.478	0.071	0.361 to inf
Intervention SHI-PT^d^	–10.070	1.264	–inf to –7.984
Recruiter_C1	–9.584	9.730	–inf to 6.482
Recruiter_D1	–6.576	3.851	–inf to –0.209
Recruiter_E1	3.182	1.873	0.088 to inf
Recruiter_F1	5.657	6.931	–5.788 to inf
Recruiter_G1	0.547	5.069	–7.824 to inf
Recruiter_H1	–0.232	5.657	–inf to 9.109
Pain medication: no	1.442	1.458	–0.965 to inf
Pain level range: 5-7	0.757	1.465	–1.662 to inf

^a^inf: infinity.

^b^KOOS_ADL_: Knee Injury and Osteoarthritis Outcome Score—Activities of Daily Living.

^c^PRE: survey before the first therapy session.

^d^SHI-PT: physiotherapy covered by statutory health insurance in Germany.

### Primary End Point: “Knee Pain” (VAS Pain Score)

In IG DHA, the pooled mean VAS pain score decreased from 48.2 points (95% CI 46.1-50.3 points) to 25.6 points (95% CI 23.5-27.8 points) from PRE to POST. This corresponds to an improvement in “knee pain” of –22.5 points (95% CI –25.2 to –19.9 points).

In CG SHI-PT, pooled mean VAS pain score improved by –6.5 points (95% CI –8.7 to –4.4 points), from PRE 44.7 points (95% CI 42.4-47.1 points) to POST 38.2 points (95% CI 35.3 p-41.0 points).

**Figure 5:**
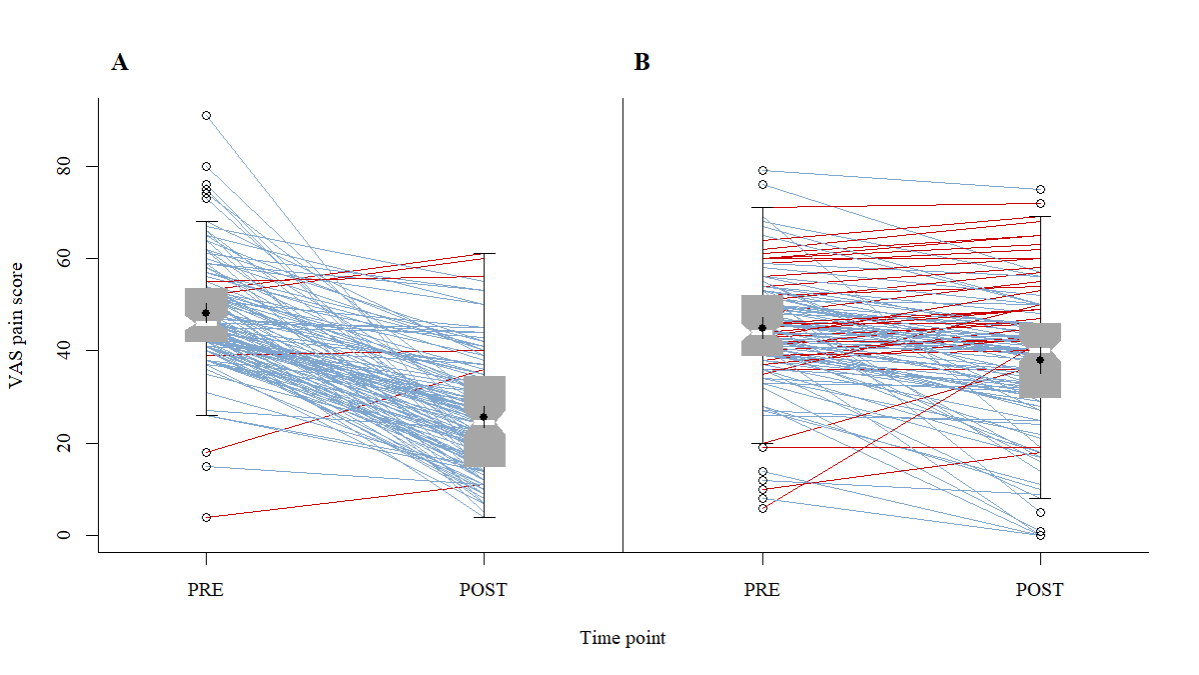
The comparison of visual analog scale (VAS) pain score between PRE and POST intervention surveys (A: IG DHA; and B: CG SHI-PT), using the imputed dataset with the largest effect for CG SHI-PT. Each panel displays notched boxplots (95% CI of median), including mean with 95% CI. Intraindividual positive treatment effects are in blue and negative or no effects are in red.

Thus, training with the DHA resulted in a 3.5 times greater reduction in “knee pain” compared with SHI-PT. Figure 5 illustrates this result using the imputed dataset with the greatest effect for CG SHI-PT.

In the pooled ANCOVA for VAS pain score, the group difference between IG DHA and CG SHI-PT was also statistically significant (*P*<.001; Table 5), and was estimated at 14.3 points (11.7 points-infinity; adjusted 1-sided 95% CI; Table 6).

**Table 5:** Analysis of covariance (ANCOVA) “knee pain” (R2=0.392).

	SSQ^a^	*df*1	*F*	*P* value	*η* ^2^	Partial *η*^2^
VAS^b^ pain score PRE^c^	8395.020	1	51.784	<.001	0.138	0.185
Intervention	12,855.179	1	82.779	<.001	0.211	0.258
Recruiter	2259.651	6	2.185	.04	0.037	0.058
Pain medication	214.351	1	1.391	.24	0.004	0.006
Pain level	164.886	1	0.974	.32	0.003	0.004
Residual	36,984.465	—^d^	—	—	—	—

^a^SSQ: sum of squares.

^b^VAS: visual analogue scale.

^c^PRE: survey before the first therapy session.

^d^Not applicable.

**Table 6:** Analysis of covariance (ANCOVA) “knee pain” pooled estimates with adjusted 1-sided 95% CI.

Term	Coefficient	SE	95% CI
Intercept	5.629	4.319	–1.504 to inf^a^
VAS^b^ pain score PRE^c^	0.441	0.084	0.302 to inf
Intervention SHI-PT^d^	14.328	1.596	11.692 to inf
Recruiter_C1	5.349	12.451	–15.210 to inf
Recruiter_D1	7.618	4.683	–0.117 to inf
Recruiter_E1	–6.155	2.165	–inf to –2.579
Recruiter_F1	–3.658	8.825	–inf to 10.913
Recruiter_G1	–2.330	6.519	–inf to 8.435
Recruiter_H1	8.950	7.263	–3.043 to inf
Pain medication: no	–1.719	1.861	–inf to 1.355
Pain level range: 5-7	2.018	2.041	–1.353 to inf

^a^inf: infinity.

^b^VAS: visual analog scale.

^c^PRE: survey before the first therapy session.

^d^SHI-PT: physiotherapy covered by statutory health insurance in Germany.

### Pain Medication and Concomitant Care

During the course of the intervention, the reported use of pain medication decreased substantially in IG DHA (PRE: 35/132, 26.5%; POST: 4/132, 3.0%), while it remained almost unchanged in CG SHI-PT (PRE: 37/121, 30.6%; POST: 33/121, 27.3%). The difference POST–PRE was -23.5% (–31/132) for IG DHA, and –3.3% (–4/121) for CG SHI-PT. Only a few participants provided no information on pain medication usage (PRE CG SHI-PT: 1/121, 0.8%; POST IG DHA: 1/132, 0.8%; POST CG SHI-PT: 4/121, 3.3%).

Table 7 enumerates the patients who received concomitant care during the course of the study. In the IG DHA group, no patient used additional physiotherapy. In contrast, of 121 patients, 1 patient (0.8%) in the CG SHI-PT group underwent additional physiotherapy sessions. The other concomitant therapies were as follows: ultrasound therapy, shock-wave therapy, cortisone injection into the knee, massage, strengthening exercises, yoga, and meditation.

**Table 7:** Number of participants using concomitant care.

Group	PRE^a^, n (%)	POST^b^, n (%)	Difference POST–PRE, n (%)
IG DHA^c^ (n=132)	4 (3)	1 (0.8)	–3 (–2.2)
CG SHI-PT^d^ (n=121)	5 (4.1)	4 (3.3)	–1 (–0.8)

^a^PRE: survey before the first therapy session.

^b^POST: survey after completing the treatment period of 12 weeks.

^c^IG DHA: intervention group using digital health application.

^d^CG SHI-PT: control group with physiotherapy covered by statutory health insurance in Germany.

### Nonprespecified Analyses

After extending the ANCOVA models with the factor “sex” and the covariate “age” (numerical), including interaction effects with factor “intervention,” no statistically significant isolated effect of either “sex” or “age” on “knee function” and “knee pain” was found (*P* value range=.28-.91; Tables 8 and 9). The effect sizes (partial *η*^2^) of “sex” and “age” are negligible and explain almost no additional variance compared to the statistical models without these factors or covariates (Tables 8 and 9). For the interaction effects “intervention:age” and “intervention:sex,” again no statistically significant effect was found for either end point (*P* value range=.48-.74; Tables 8 and 9). The effect sizes (partial *η*^2^) of the 2 interaction effects can be described as negligible and provide almost no additional explanation (Tables 8 and 9).

**Figure 6:**
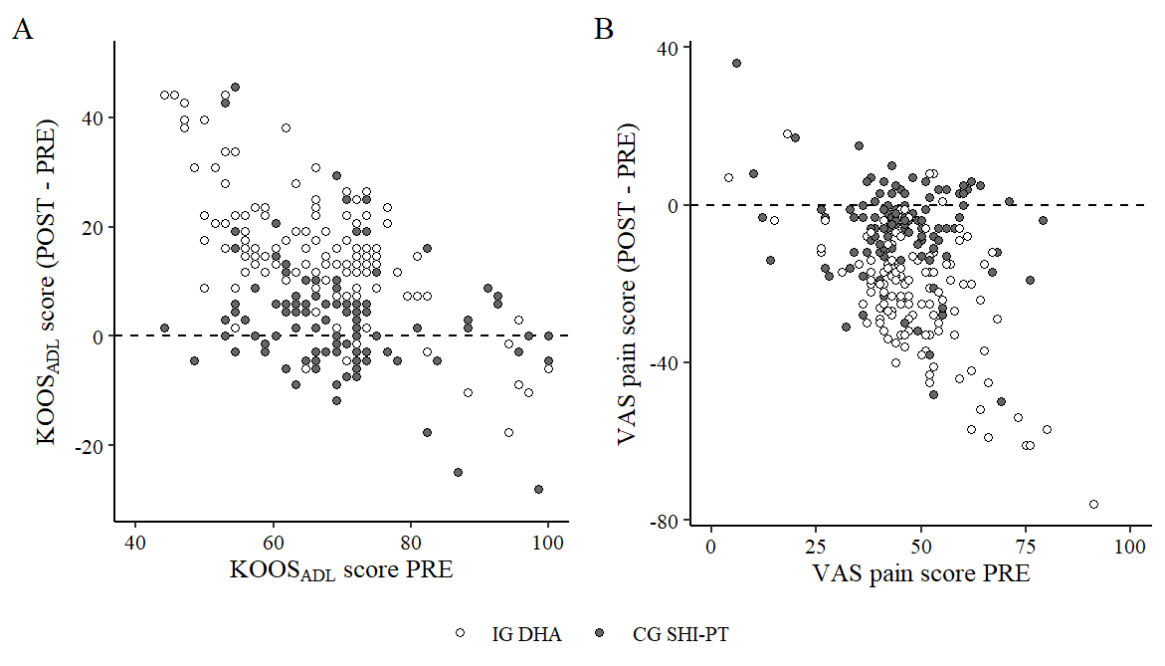
Relationship between PRE score and treatment success (POST score–PRE score) for both end points. A: “knee function,” B: “knee pain.” The intervention group (IG) with the digital health application (DHA) data are in white and the control group (CG) with physiotherapy covered by statutory health insurance in Germany (SHI-PT) data are in gray. KOOS_ADL_: Knee Injury and Osteoarthritis Outcome Score—Activities of Daily Living; VAS: visual analog scale.

**Figure 7:**
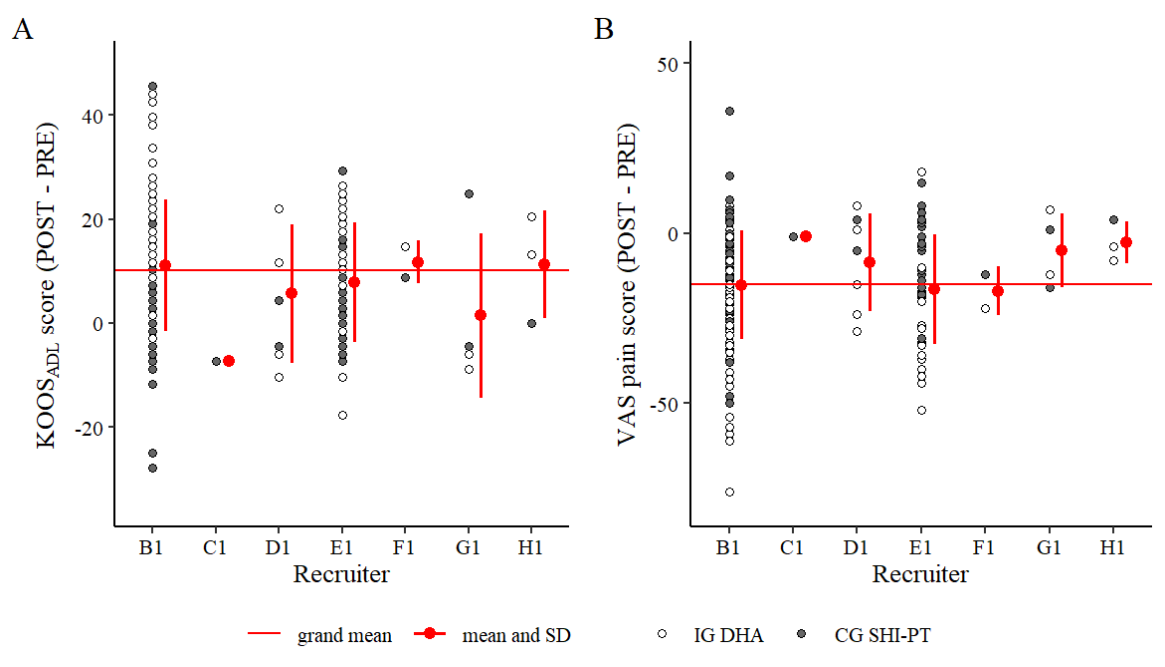
Treatment success (POST score–PRE score) for both end points across all recruiters. A: “knee function” by recruiter; B: “knee pain” by recruiter. The intervention group (IG) with the digital health application (DHA) data are in white and the control group (CG) with physiotherapy covered by statutory health insurance in Germany (SHI-PT) data are in gray. KOOS_ADL_: Knee Injury and Osteoarthritis Outcome Score—Activities of Daily Living; VAS: visual analog scale.

Figures 6 and 7 display a descriptive analysis of both statistically significant covariates (PRE score and recruiter), the relationship between PRE scores and treatment success (POST–PRE scores), as well as recruiter-specific effects (treatment success across different recruiters) for both end points.

**Table 8:** Analysis of covariance (ANCOVA) “knee function” with the factor “sex” and the covariate “age” (numerical), including interaction effects with the factor “intervention” (R2=0.402).

	SSQ^a^	*df* 1	*F*	*P* value	*η* ^2^	Partial *η*^2^
KOOS_ADL_^b^ score PRE^c^	6851.619	1	62.332	<.001	0.189	0.240
Intervention	6548.347	1	68.131	<.001	0.180	0.232
Sex	4.004	1	0.028	.87	<0.001	<0.001
Age	79.046	1	0.786	.38	0.002	0.004
Recruiter	845.762	6	1.093	.36	0.023	0.037
Pain medication	99.632	1	1.046	.31	0.003	0.005
Pain level	106.118	1	1.038	.31	0.003	0.005
Intervention: sex	13.906	1	0.112	.74	<0.001	0.001
Intervention: age	46.122	1	0.440	.51	0.001	0.002
Sex: age	2.104	1	0.012	.91	<0.001	<0.001
Intervention: sex: age	4.034	1	0.028	.87	<0.001	<0.001
Residual	21,732.292	—^d^	—	—	—	—

^a^SSQ: sum of squares.

^b^KOOS_ADL_: Knee Injury and Osteoarthritis Outcome Score—Activities of Daily Living.

^c^PRE: survey before the first therapy session.

^d^Not applicable.

**Table 9:** Analysis of covariance (ANCOVA) “knee pain” with the factor “sex” and the covariate “age” (numerical), including interaction effects with factor “intervention” (R2=0.405).

	SSQ^a^	*df* 1	*F*	*P* value	*η* ^2^	Partial *η*^2^
KOOS_ADL_^b^ score PRE^c^	8309.270	1	50.873	<.001	0.139	0.190
Intervention	13,146.396	1	84.324	<.001	0.220	0.270
Sex	2.885	1	0.013	.91	<0.001	<0.001
Age	185.610	1	1.177	.28	0.003	0.005
Recruiter	2075.138	6	2.013	.06	0.035	0.055
Pain medication	249.414	1	1.612	.20	0.004	0.007
Pain level	38.459	1	0.176	.68	0.001	0.001
Intervention: sex	28.349	1	0.167	.68	<0.001	0.001
Intervention: age	82.436	1	0.506	.48	0.001	0.002
Sex: age	43.666	1	0.268	.61	0.001	0.001
Intervention: sex: age	5.144	1	0.022	.88	<0.001	<0.001
Residual	35,484.314	—^d^	—	—	—	—

^a^SSQ: sum of squares.

^b^KOOS_ADL_: Knee Injury and Osteoarthritis Outcome Score—Activities of Daily Living.

^c^PRE: survey before the first therapy session.

^d^Not applicable.

## Discussion

### Primary Outcomes

This study provides evidence of the clinical superiority of DHA over SHI-PT for both primary end points, “knee function” (KOOS_ADL_ score) and “knee pain” (VAS pain score).

#### Knee Function

The improvement achieved by IG DHA in our study (15.7 points KOOS_ADL_ score) not only meets the criterion of clinical relevance, but also that of “substantial clinical benefit,” suggested by several previous studies [57,59,76]. The improvement through SHI-PT was 4.5 times lower than in IG DHA and did not reach clinical relevance in our study.

Previous uncontrolled research on the efficacy of the DHA Mawendo reported a 14% improvement in the Kujala score over 12 weeks [10]. In this study, “knee function” improved by 23.7% in IG DHA compared to 4.8% in CG SHI-PT, further underscoring the effectiveness of the DHA. Regarding the external validity of our findings, companion patella [30], a similar DHA for *ICD-10* M22 conditions, was also evaluated against SHI-PT in a multicenter, prospective RCT. This study is particularly relevant as it was also conducted within the German health care system. The authors found DHA to be superior to SHI-PT in improving knee function, as assessed using the Kujala score (+8.68 points, 95% CI 3.74-13.62 points; *P*<.001). The study, including its results, is listed in the DiGA-Verzeichnis of the German Federal Institute for Drugs and Medical Devices [29,30], indicating that it underwent thorough regulatory review. Although no further studies were identified that directly compare DHA to SHI-PT, other research supports the general efficacy of home-based exercise interventions for treating patellar conditions. Hong et al [77] examined the effectiveness of a 6-week home-based exercise program combined with health education via remote support in patients with patellofemoral pain. Their RCT (CG health education) found that this approach improved function by 14.6%, as measured by the Anterior Knee Pain Scale (CG by 2.4%, between-group difference 9 points, 95% CI 4.1-13.9; *P*<.01). Similarly, Nilmart et al [78] reported that a 4-week telehealth-based therapeutic exercise program with real-time supervision significantly enhanced various functional parameters in patients with patellofemoral pain. In contrast, the CG, which engaged in self-guided stretching exercises, exhibited minimal change. In addition, Mecklenburg et al [58] compared a 12-week “Digital Care Program for Chronic Knee Pain” to an education-only CG and observed a 17% reduction in KOOS—Physical Function Short-form score in IG DHA, compared to only 3.7% in CG SHI-PT. The difference between the IG and CG was –7.2 points (95% CI –11.5 to –3 points; *P*=.001), further demonstrating the potential benefits of digitally assisted home exercise programs.

#### Knee Pain

The pain reduction of 22.5 points (VAS pain score) in IG DHA classifies as clinically relevant, and as substantially clinically beneficial [60,61]. We observed no clinically relevant effects for pain reduction through SHI-PT in our study. The achieved reduction in “knee pain” using the DHA Mawendo was 47% compared to 14% through SHI-PT.

Using the same DHA and treatment period as in this study, Kölle et al [10] reported a 64% reduction in knee pain on a knee pain VAS. Other studies investigating DHA interventions for patellofemoral pain support these results. DHA companion patella [30] demonstrated significant and clinically relevant pain reduction on a numeric pain rating scale (between-group difference –1.5, 95% CI –2.18 to –0.81; *P*<.001). In addition, Hong et al [77] found improved pain outcomes (pain reduction up to 51.6%) for home-based exercise program with health education using a VAS range 1 to 100 mm (worst pain reduction, between-group difference –19.3, 95% CI –23.2 to –15.5; *P*<.01 and pain with daily activity, between-group difference –22.9, 95% CI –28.3 to –17.4; *P*<.01). In comparison, the reduction in knee pain in the CG was minimal at a maximum of 5.2% [77]. Nilmart et al [78] reported that a 4-week telehealth-supervised therapeutic exercise program significantly reduced VAS pain (0-10 cm) by 67.3% in patients with patellofemoral pain (–3.7 cm, 95% CI –4.2 to –3.3 cm; *P*<.001). In contrast, the CG (self-guided stretching) exhibited minimal change (–0.1 cm, 95% CI –0.8 cm to 0.4 cm; *P*=.80) [78]. Similar patterns were observed for DHAs in other orthopedic conditions. Toelle et al [31] found a 47% reduction in back pain using the Kaia DHA versus 37% for physiotherapy, while Weise et al [32] reported a 53.1% reduction in back pain using the Vivira DHA versus 14.6% for physiotherapy. These findings reinforce the effectiveness of structured remote exercise programs in managing musculoskeletal pain. Together, these studies underscore the mounting evidence base supporting the use of DHA-assisted interventions for enhancing knee function and alleviating pain.

#### Comparison of Treatment Arms

We found large between-group differences for both end points in our study. One reason may be the different treatment strategies used by different physiotherapists. Furthermore, an earlier start of treatment is associated with faster healing [79-82], and problems scheduling timely appointments may, in turn, limit the efficacy of SHI-PT. In comparison, self-managed training using a DHA can begin immediately after being prescribed by a physician and eliminates the need for travel, further anamnesis, and potential waiting for appointments. DHAs also allow for more treatment sessions during a 12-week intervention period, since the German regulations usually limit SHI-PT to 6 to 12 physiotherapy sessions (maximum 18 sessions) during the same period. In IG DHA, patients should exercise 2 to 3 times per week. Since self-reported adherence was comparable between IG DHA and CG SHI-PT, patients in IG DHA received at least roughly twice the number of therapy sessions compared to CG SHI-PT. We hypothesize the greater training frequency to be one of the causal reasons for the significantly greater treatment success of DHA compared to SHI-PT. The actual start of the treatment after diagnosis and the exact treatment frequency were not recorded in this study. Therefore, further research should address the relationship between onset of treatment, treatment frequency, and therapy success using DHAs.

### Further Benefits of DHA

In both treatment arms, around 30% of patients used pain medication at PRE. Pain medication rate at POST decreased substantially to 3% for IG DHA, but remained largely unchanged in CG SHI-PT. The reduction in pain medication corresponds to the greater pain reduction achieved in IG DHA. Therefore, DHA may also have indirect beneficial health effects by limiting the risk of pain medication (WHO level 1), such as nonsteroidal anti-inflammatory drug–induced side effects and associated costs.

### Statistically Significant Covariates in the ANCOVA

As expected, a statistically significant effect of the PRE scores was found for both end points [83]. Accordingly, participants with a worse PRE score tended to achieve greater improvements (Figure 6). However, the distribution of PRE scores was similar in both treatment groups (IG DHA, CG SHI-PT), indicating a limited risk of confounding. The control variable “recruiter” showed a statistically significant effect on the end point “knee pain,” while not being statistically significant on the end point “knee function.” Our statistical modeling results suggest that recruiting facilities affect treatment effectiveness. This effect is subject to a high degree of uncertainty, as recruiting performance across the 7 recruiting centers in our study varied considerably. In total, 93% (241/259) of all participants were recruited by 2 recruiting centers, while the remaining centers only acquired 1 to 8 participants, each. This suggests that this effect stems from small subsamples, which are more likely to produce extreme results. Figure 7 illustrates the distribution of treatment success (mean difference: POST–PRE) in both end points across all recruiters, for the end point “knee pain,” slightly lower recruiter variances led to statistical significance in our ANCOVA model (*P*=.04).

### Adherence, Change of Therapy, and Concomitant Care

In both treatment arms, self-reporting revealed a comparably high level of adherence: 89.4% of IG DHA patients and 85.1% of CG SHI-PT patients attended “all” or “almost all” therapy sessions. Thus, treatment frequency, and not adherence, may provide a better explanation for the between-group differences found in our study. In addition, similar treatment changes occurred in both treatment arms (1 participant in each group (1/132, 0.8%; 1/121, 0.8%); no information for 2/132, 1.5% participants in DHA, and 7/121, 5.8% participants in SHI-PT). We found a slightly larger POST dropout rate in SHI-PT (4/121, 3.3% in SHI-PT vs 1/132, 0.8% in DHA). The reasons for this are speculative and may include the aforementioned scheduling and travel required for SHI-PT. In both treatment groups, the number of patients receiving concomitant care was minimal. No patient in the IG DHA group used additional physiotherapy, and the other concomitant therapies were comparable between IG DHA and CG SHI-PT. Consequently, any potential bias attributable to concomitant care is highly improbable.

### Possible Limitations of the Study

While we controlled for “recruiter,” “pain medication,” and “pain level” in the ANCOVA model, there may be further confounding variables that were not considered in the model or during randomization. Since we found no evidence for possible bias of treatment effects due to age and sex of the participants, we did not include these covariates in the prespecification of the statistical analysis. The distribution of age and sex in the treatment arms is comparable, suggesting that neither of these variables had a systematic influence on the results. For confirmation purposes, the prespecified ANCOVA model was exploratively extended for both end points to include the covariates age and sex. We found no evidence of relevant effects of age or sex on either end point, including interactions with intervention. It can therefore be concluded that neither age nor sex has a statistically significant effect on the efficacy of the IG DHA and CG SHI-PT interventions [67-73].

Blinding was not feasible in this study, as the nature of the interventions made it impossible to conceal the treatment strategy from participants or recruiters. A placebo app was not implemented, as BfArM guidelines for the evaluation of DHAs require evidence of superiority over usual care (SHI-PT), which does not include any additional DHA. A potential concern related to the lack of blinding is expectancy effects, where participants using the DHA may have rated the DHA systematically better due to its structured progression, interactive feedback, or perceived novelty compared to conventional physiotherapy. However, any resulting bias is likely limited, as all outcome measures were based on standardized, validated patient-reported outcome scores. The study did not include direct inquiries regarding the perceived quality of the intervention. While self-reported outcomes inherently carry the risk of expectancy effects, these instruments are widely used in clinical research and have established validity and reliability in similar patient populations. All outcomes in this study were assessed using validated patient-reported instruments (KOOS_ADL_ and VAS). Patients in both groups completed the surveys independently via a web-based platform, minimizing the potential for interviewer and social desirability bias. However, the use of self-reported measures introduces an unquantifiable risk of bias, particularly in the absence of blinding. Expectation effects and differences in perceived engagement may have influenced how participants, especially in the DHA group, reported pain, function, adherence, and medication use. Nevertheless, randomization helps ensure that any general tendency to over- or underreport is equally distributed between groups, thereby preserving internal validity. Given the significant and large clinically relevant between-group differences, we consider the risk of substantially biased results to be low.

Adherence was assessed subjectively through self-reported data collected at the end of the intervention period, introducing the potential for recall bias. Participants may not have accurately remembered or reported the number of sessions they completed, and responses could have been influenced by social desirability bias. Future studies could consider alternative methods, such as real-time adherence tracking, to improve accuracy. We did not use log-in data to verify adherence in the IG DHA group because we could not objectively record adherence in the CG SHI-PT group, making a valid comparison impossible. All physiotherapy patients chose their physiotherapist and made their appointments. Therefore, it was not economically feasible to objectively collect adherence data for IG SHI-PT. The same applies to the specific therapy content of the sessions received by participants in CG SHI-PT. While German statutory health insurance physiotherapy follows standardized guidelines, individual treatment strategies may vary depending on the treating physiotherapist. This variability limits direct comparability between DHA and SHI-PT. A comparison of physiotherapy administered under standardized ideal conditions and DHA could potentially yield a group difference that is less pronounced than this study. However, as our study aimed to evaluate the efficacy of DHA compared to standard care under real-world conditions (intention-to-treat), rather than to directly compare digital and face-to-face physiotherapy, the resulting heterogeneity reflects typical clinical practice and, therefore, supports the external validity of our findings.

Patients who did not respond to either the PRE or POST survey generated missing values. As we conducted this study according to the intention-to-treat principle, missing values were imputed. In total, only 6 (2.4%) incomplete datasets were available for 253 analyzed patients. Of these, 5 (2%) cases came from CG SHI-PT and 1 (0.4%) from IG DHA. Consequently, the likelihood of systematic bias in the study results due to missing values is minimal. Regardless of the imputation strategy used, the study’s findings are likely to be robust against the influence of missing values, given the substantial differences between the treatment arms.

Furthermore, we did not assess participants’ previous expertise, experience, or attitudes toward DHAs in general or Mawendo specifically. While participants in the IG had the necessary resources, it remains possible that some faced technological barriers. In addition, varying levels of digital literacy could influence the ease of use and engagement with the DHA. However, such difficulties would likely bias results in favor of SHI-PT rather than DHA, suggesting that any observed treatment effects were not artificially inflated by technology accessibility. Furthermore, given the increasing integration of digital health solutions in clinical practice, these barriers are expected to diminish over time.

### Future Research Directions

While this study provides evidence for the short-term efficacy of DHA-based interventions, future research should investigate their long-term impact. In this regard, one potential advantage of DHAs is that they can be used beyond the prescribed therapy duration, potentially extending their benefits over time. In addition to functional improvements and pain reduction, future studies should explore other clinically relevant end points, such as quality of life and additional physical and functional measures (eg, range of motion, strength, and mobility). These may be particularly relevant for specific populations, such as athletes aiming for an early return to sport or older adults focusing on fall prevention and independence.

DHAs may also have the potential to complement traditional physiotherapy rather than replace it. Their integration into routine care could enhance accessibility, provide continuous support between in-person therapy sessions, and facilitate personalized rehabilitation strategies. Future studies should explore hybrid models that combine DHAs with supervised physiotherapy to optimize patient outcomes. Furthermore, identifying patient subgroups that benefit most from DHA interventions could improve targeted therapy approaches, ensuring more efficient allocation of health care resources. Finally, investigations into usability aspects may help to further improve the effect of DHAs.

### Conclusions

Our findings indicate that the investigated DHA is superior to SHI-PT for treating disorders of the patella. Sex and age did not have any effect on the intervention outcomes of the study. In addition, posttreatment pain medication intake was substantially lower for DHA compared to SHI-PT. Therefore, DHA Mawendo has been approved by BfArM for persons of all sexes aged ≥12 years for the treatment of disorders of the patella.

## Appendix

Multimedia Appendix 1 Digital health application Mawendo International Classification of Diseases code M22 phases and exercises.

Multimedia Appendix 2 Relevant literature for digital health application Mawendo International Classification of Diseases code M22 exercise compilation.

Multimedia Appendix 3 List of pain medications used.

Multimedia Appendix 4 CONSORT-eHEALTH checklist (V 1.6.1).

## Abbreviations

ANCOVA: analysis of covariance
BfArM: Bundesinstitut für Arzneimittel und Medizinprodukte
CG: control group
CONSORT: Consolidated Standards of Reporting Trials
DHA: digital health application
ICD-10: International Classification of Diseases, Tenth Revision
IG: intervention group
KOOS: Knee Injury and Osteoarthritis Outcome Score
KOOS_ADL_: Knee Injury and Osteoarthritis Outcome Score—Activities of Daily Living
POST: survey after completing the treatment period of 12 weeks
PRE: survey before the first therapy session
RCT: randomized controlled trial
SHI-PT: physiotherapy covered by statutory health insurance in Germany
VAS: visual analog scale
VNRS: Verbal Numerical Rating Scale
WHO: World Health Organization

## Data Availability

The datasets generated or analyzed during this study are available from the corresponding author on reasonable request.
