# Supplementary material for: Effectiveness of Self-Training With a Web-Based Digital Health Application Versus Physiotherapy in the Treatment of Disorders of the Patella: Randomized Controlled Trial
Source: J Med Internet Res. 2025 May 5;27:e66463. doi: 10.2196/66463 (PMC12089869; doi:10.2196/66463)
Supplement: Multimedia Appendix 1 [file jmir_v27i1e66463_app1.pdf]

Detailed information on exercises for ICD-10 M22

Objectives of the Three Successive Phases

The default duration is 4 weeks for each phase, but this may be modified by the prescribing physician.

Phase 1: Introduction and Adaptation

Objective: Introduce simple exercises with minimal load to allow participants to become familiar with the movements while maintaining a focus on precision and awareness of pain thresholds.

Key Features:

- Familiarization with movement patterns
- Small ranges of motion
- Low exercise intensity

Phase 2: Consolidation and Progression

Objective: Advance to slightly more challenging exercises that improve coordination and address muscle imbalances while still preventing overexertion.

Key Features:

- Enhancement of coordinated movement sequences
- Reduction of muscular imbalances
- Incremental increase in load intensity

Phase 3: Optimization and Transition to Normal Load

Objective: Facilitate further progress toward full joint and muscle functionality by normalizing movement patterns and optimizing load capacity. Regular practice of these exercises is recommended for sustained benefits.

Key Features:

- Normalization of movement sequences
- Improvement in load-bearing capacity
- Transition to varied and regular exercise routines

Detailed Exercises

Each phase's exercises are demonstrated in the app via instructional videos. The exercises are detailed as follows:

Phase 1 Exercises

1. Seated Hip Mobilization on a Gym Ball
2. Standing Hip Lift
3. Step Up – Step Down
4. Small Lateral Step
5. Standing Lateral Thigh Strengthening (Light Variation)
6. Single-Leg Squat with Reduced Knee Flexion
7. Small Plie Squat with Reduced Knee Flexion
8. Seated Leg Extension with TheraBand (Light Variation)
9. Standing Lateral Thigh Stretch
10. Side-Lying Anterior Thigh Stretch with Gym Band
11. Self-Massage of the Lateral Thigh
12. Lateral Thigh Massage

Phase 2 Exercises

1. Standing Hip Awareness Exercise
2. Gait Over Obstacles (Light Variation)
3. Step Up – Step Down
4. Large Lateral Step
5. Standing Lateral Thigh Strengthening
6. Moderate Squat with a Ball (Medium Knee Flexion Angle)
7. Single-Leg Squat with Gym Band (with Inward Pull)
8. Seated Leg Extension with TheraBand (with External Rotation)
9. Seated Lateral Thigh Stretch
10. Side-Lying Anterior Thigh Stretch
11. Lateral Thigh Massage

Phase 3 Exercises

1. Standing Hip Awareness Exercise with Eyes Closed
2. Gait Over Obstacles (Challenging Variation)
3. Step Up – Step Down
4. Walking with TheraBand
5. Lying Lateral Thigh Strengthening with Gym Band
6. Large Lunge with Reduced Knee Flexion
7. Single-Leg Squat on an Unstable Surface (Balance Pad)
8. Squat with TheraBand and Airex on an Unstable Surface (Small Knee Flexion Angle)
9. Seated Leg Extension with TheraBand (with External Rotation)
10. Lateral Thigh Stretch at a Table
11. Standing Anterior Thigh Stretch
12. Lateral Thigh Massage Using a Foam Roller
