# Supplementary material for: Effectiveness of Self-Training With a Web-Based Digital Health Application Versus Physiotherapy in the Treatment of Disorders of the Patella: Randomized Controlled Trial
Source: J Med Internet Res. 2025 May 5;27:e66463. doi: 10.2196/66463 (PMC12089869; doi:10.2196/66463)
Supplement: Multimedia Appendix 2 [file jmir_v27i1e66463_app2.pdf]

4.1 Relevante Artikel zur Auswahl der Übungen (2):

1. *_arabon, D. M. M. Z. D. S. N. (2022). Strength, Flexibility and Postural Control of the Trunk and Lower Body in Participants with and without Patellofemoral Pain. Applied Sciences, Vol 12, Iss 3238, p, 3238(eng).*
2. *Ahmed Hamada, H., Hussein Draz, A., Koura, G. M., & Saab, I. M. (2017). Carryover effect of hip and knee exercises program on functional performance in individuals with patellofemoral pain syndrome. Journal of Physical Therapy Science, 29(8), 1341-1347. https://doi.org/10.1589/jpts.29.1341*
3. *Alammari, A., Spence, N., Narayan, A., Karnad, S. D., & Ottayil, Z. C. (2023). Effect of hip abductors and lateral rotators' muscle strengthening on pain and functional outcome in adult patients with patellofemoral pain: A systematic review and meta-analysis. Journal of back and musculoskeletal rehabilitation, 36(1), 35-60. https://doi.org/10.3233/BMR-220017*
4. *Alba-Martín, P., Gallego-Izquierdo, T., Plaza-Manzano, G., Romero-Franco, N., Núñez-Nagy, S., & Pecos-Martín, D. (2015). Effectiveness of therapeutic physical exercise in the treatment of patellofemoral pain syndrome: a systematic review. Journal of Physical Therapy Science, 27(7), 2387-2390. https://doi.org/10.1589/jpts.27.2387*
5. *Albornoz-Cabello, M., Barrios-Quinta, C. J., Barrios-Quinta, A. M., Escobio-Prieto, I., Cardero-Durán, M. d. L. A., & Espejo-Antunez, L. (2021). Effectiveness of Tele-Prescription of Therapeutic Physical Exercise in Patellofemoral Pain Syndrome during the COVID-19 Pandemic. International journal of environmental research and public health, 18(3). https://doi.org/10.3390/ijerph18031048*
6. *Aliberti, S. P., Mezêncio, B. P., Amadio, A. C. P., Serrão, J. C. P., & Mochizuki, L. P. (2019). Immediate effects of a distal gait modification during stair descent in individuals with patellofemoral pain. Physiotherapy Theory and Practice, 35(12), 1243-1249. https://doi.org/10.1080/09593985.2018.1477212*
7. *Almeida, G. P. L., Rodrigues, H. L. d. N., Coelho, B. A. L., Rodrigues, C. A. S., & Lima, P. O. d. P. (2021). Anteromedial versus posterolateral hip musculature strengthening with dose-controlled in women with patellofemoral pain: A randomized controlled trial. Physical therapy in sport : official journal of the Association of Chartered Physiotherapists in Sports Medicine, 49, 149-156. https://doi.org/10.1016/j.ptsp.2021.02.016*
8. *Almeida, G. P. L., Silva, A. P. d. M. C. C. E., França, F. J. R., Magalhães, M. O., Burke, T. N., & Marques, A. P. (2016). Relationship between frontal plane projection angle of the knee and hip and trunk strength in women with and without patellofemoral pain. Journal of back and musculoskeletal rehabilitation, 29(2), 259-266. https://doi.org/10.3233/BMR-150622*
9. *Almeida, G. P. L., Silva, A. P. d. M. C. C. E., França, F. J. R., Magalhães, M. O., Burke, T. N., & Marques, A. P. (2016). Q-angle in patellofemoral pain: relationship with dynamic knee valgus, hip abductor torque, pain and function. Revista brasileira de ortopedia, 51(2), 181-186. https://doi.org/10.1016/j.rboe.2016.01.010*
10. *Alsaleh, S. A., Murphy, N. A., Miller, S. C., Morrissey, D., & Lack, S. D. (2021). Local neuromuscular characteristics associated with patellofemoral pain: A systematic review and meta-analysis. Clinical biomechanics (Bristol, Avon), 90, 105509. https://doi.org/10.1016/j.clinbiomech.2021.105509*
11. *Arendt, E. (2012). Hip-strengthening exercises before functional exercises reduced pain in women with patellofemoral pain syndrome. The Journal of bone and joint surgery. American volume, 94(10), 940. https://doi.org/10.2106/JBJS.9410.ebo274*
12. *Arora, R. K. R. A. L. (2016). Efficacy of posterolateral hip muscles strengthening on patellofemoral pain. International Journal of Research in Medical Sciences, Vol 4, Iss 7, Pp 2921-, 2925(eng).*
13. *Arslan, T., & Gültekin, M. Z. (2023). The effect of a supervised online group exercise program on symptoms associated with patellofemoral pain syndrome in women. Technology and health care : official journal of the European Society for Engineering and Medicine, 31(2), 771-782. https://doi.org/10.3233/THC-220533*
14. *Ashnagar, Z., Hadian, M.-R., Sajjadi, E., Kajbafvala, M., Olyaei, G., Pashazadeh, F., & Rezasoltani, A. (2021). Quadriceps architecture in individuals with patellofemoral pain: A systematic review. Journal of bodywork and movement therapies, 25, 248-254. https://doi.org/10.1016/j.jbmt.2020.08.007*

15. *Azab, A. R., Abdelbasset, W. K., Basha, M. A., Mahmoud, W. S., Elsayed, A. E., Saleh, A. K., & Elnaggar, R. K. (2022). Incorporation of Pilates-based core strengthening exercises into the rehabilitation protocol for adolescents with patellofemoral pain syndrome: a randomized clinical trial. European review for medical and pharmacological sciences, 26(4), 1091-1100. https://doi.org/10.26355/eurrev_202202_28098*
16. *Bagheri, S., Naderi, A., Mirali, S., Calmeiro, L., & Brewer, B. W. (2021). Adding Mindfulness Practice to Exercise Therapy for Female Recreational Runners With Patellofemoral Pain: A Randomized Controlled Trial. Journal of athletic training, 56(8), 902-911. https://doi.org/10.4085/1062-6050-0214.20*
17. *Baldon, R. d. M., Serrão, F. V., Scattone Silva, R., & Piva, S. R. (2014). Effects of functional stabilization training on pain, function, and lower extremity biomechanics in women with patellofemoral pain: a randomized clinical trial. The Journal of orthopaedic and sports physical therapy, 44(4), 240-251, A241-A248. https://doi.org/10.2519/jospt.2014.4940*
18. *Barton, C. J., de Oliveira Silva, D., Patterson, B. E., Crossley, K. M., Pizzari, T., & Nunes, G. S. (2019). A proximal progressive resistance training program targeting strength and power is feasible in people with patellofemoral pain. Physical therapy in sport : official journal of the Association of Chartered Physiotherapists in Sports Medicine, 38, 59-65. https://doi.org/10.1016/j.ptsp.2019.04.010*
19. *Barton, C. J., Lack, S., Hemmings, S., Tufail, S., & Morrissey, D. (2015). The 'Best Practice Guide to Conservative Management of Patellofemoral Pain': incorporating level 1 evidence with expert clinical reasoning. British journal of sports medicine, 49(14), 923-934. https://doi.org/10.1136/bjsports-2014-093637*
20. *Barton, C. J., Lack, S., Malliaras, P., & Morrissey, D. (2013). Gluteal muscle activity and patellofemoral pain syndrome: a systematic review. British journal of sports medicine, 47(4), 207-214. https://doi.org/10.1136/bjsports-2012-090953*
21. *Bennell, K., Duncan, M., Cowan, S., McConnell, J., Hodges, P., & Crossley, K. (2010). Effects of vastus medialis oblique retraining versus general quadriceps strengthening on vasti onset. Medicine and science in sports and exercise, 42(5), 856-864. https://doi.org/10.1249/MSS.0b013e3181c12771*
22. *Bhattacharya, U. R., R. (2015). ACTIVATION OF VASTUS MEDIALIS OBLIQUES AT DIFFERENT KNEE ANGLES IN CLOSED KINETIC CHAIN AND OPEN KINETIC CHAIN POSITION IN SUBJECTS WITH PATELLO FEMORAL PAIN SYNDROME. International Journal of Physiotherapy, Vol 2, Iss, 3(eng).*
23. *Bolgla, L. A., & Boling, M. C. (2011). An update for the conservative management of patellofemoral pain syndrome: a systematic review of the literature from 2000 to 2010. International journal of sports physical therapy, 6(2), 112-125. https://www.ncbi.nlm.nih.gov/pmc/articles/PMC3109895/pdf/ijspt-06-112.pdf*
24. *Bolgla, L. A., Boling, M. C., Mace, K. L., DiStefano, M. J., Fithian, D. C., & Powers, C. M. (2018). National Athletic Trainers' Association Position Statement: Management of Individuals With Patellofemoral Pain. Journal of athletic training, 53(9), 820-836. https://doi.org/10.4085/1062-6050-231-15*
25. *Bolgla, L. A., Earl-Boehm, J., Emery, C., Hamstra-Wright, K., & Ferber, R. (2015). Comparison of hip and knee strength in males with and without patellofemoral pain. Physical therapy in sport : official journal of the Association of Chartered Physiotherapists in Sports Medicine, 16(3), 215-221. https://doi.org/10.1016/j.ptsp.2014.11.001*
26. *Bolgla, L. A., Earl-Boehm, J., Emery, C., Hamstra-Wright, K., & Ferber, R. (2016). PAIN, FUNCTION, AND STRENGTH OUTCOMES FOR MALES AND FEMALES WITH PATELLOFEMORAL PAIN WHO PARTICIPATE IN EITHER A HIP/CORE- OR KNEE-BASED REHABILITATION PROGRAM. International journal of sports physical therapy, 11(6), 926-935. https://www.ncbi.nlm.nih.gov/pmc/articles/PMC5095944/pdf/ijspt-11-926.pdf*
27. *Bolgla, L. A., Malone, T. R., Umberger, B. R., & Uhl, T. L. (2008). Hip strength and hip and knee kinematics during stair descent in females with and without patellofemoral pain syndrome. The Journal of orthopaedic and sports physical therapy, 38(1), 12-18. https://doi.org/10.2519/jospt.2008.2462*
28. *Bolgla, L. A., Malone, T. R., Umberger, B. R., & Uhl, T. L. (2011). Comparison of hip and knee strength and neuromuscular activity in subjects with and without patellofemoral pain syndrome. International journal of sports physical therapy, 6(4), 285-296. https://www.ncbi.nlm.nih.gov/pmc/articles/PMC3230156/pdf/ijspt-06-285.pdf*
29. *Boling, M., & Padua, D. (2013). Relationship between hip strength and trunk, hip, and knee kinematics during a jump-landing task in individuals with patellofemoral pain. International journal of sports physical therapy, 8(5), 661-669. https://www.ncbi.nlm.nih.gov/pmc/articles/PMC3811740/pdf/ijspt-10-661.pdf*
30. *Brian J. Theisen, P. T., D.P.T., S.C.S., A.T.C. ; Peter D. Larson, P.T., D.P.T., O.C.S. ; Caitlin C. Chambers, M.D. (2022). Optimizing Rehabilitation and Return to Sport in Athletes With Anterior Knee Pain Using a Biomechanical Perspective. Arthroscopy, Sports Medicine, and Rehabilitation, Vol 4, Iss 1, Pp e199-e, 207(eng).*

31. Briani, R. V., Waiteman, M. C., de Albuquerque, C. E., Gasoto, E., Segatti, G., Oliveira, C. B., de Azevedo, F. M., & de Oliveira Silva, D. (2019). Lower Trunk Muscle Thickness Is Associated With Pain in Women With Patellofemoral Pain. Journal of ultrasound in medicine : official journal of the American Institute of Ultrasound in Medicine, 38(10), 2685-2693. https://doi.org/10.1002/jum.14973
32. Callaghan, M. J. (2018). Exercise is effective for patellofemoral pain, but what type, who benefits most and by how much remain unknown. British journal of sports medicine, 52(10), 625-626. https://doi.org/10.1136/bjsports-2017-098296
33. Capin, J. J., & Snyder-Mackler, L. (2018). The current management of patients with patellofemoral pain from the physical therapist's perspective. Annals of Joint, 3, 40. https://doi.org/10.21037/aoj.2018.04.11
34. Carry, P. M., Gala, R., Worster, K., Kanai, S., Miller, N. H., James, D., Provance, A. J., & Carollo, J. J. (2017). POSTURAL STABILITY AND KINETIC CHANGE IN SUBJECTS WITH PATELLOFEMORAL PAIN AFTER A NINE-WEEK HIP AND CORE STRENGTHENING INTERVENTION. International journal of sports physical therapy, 12(3), 314-323. https://www.ncbi.nlm.nih.gov/pmc/articles/PMC5455181/pdf/ijspt-12-314.pdf
35. Chang, W.-D., Huang, W.-S., Lee, C.-L., Lin, H.-Y., & Lai, P.-T. (2014). Effects of open and closed kinetic chains of sling exercise therapy on the muscle activity of the vastus medialis oblique and vastus lateralis. Journal of Physical Therapy Science, 26(9), 1363-1366. https://doi.org/10.1589/jpts.26.1363
36. Chevidikunnan, M. F., Al Saif, A., Gaowgzeh, R. A., & Mamdouh, K. A. (2016). Effectiveness of core muscle strengthening for improving pain and dynamic balance among female patients with patellofemoral pain syndrome. Journal of Physical Therapy Science, 28(5), 1518-1523. https://doi.org/10.1589/jpts.28.1518
37. Clijsen, R., Fuchs, J., & Taeymans, J. (2014). Effectiveness of exercise therapy in treatment of patients with patellofemoral pain syndrome: systematic review and meta-analysis. Physical therapy, 94(12), 1697-1708. https://doi.org/10.2522/ptj.20130310
38. Collins, N. J., Barton, C. J., van Middelkoop, M., Callaghan, M. J., Rathleff, M. S., Vicenzino, B. T., Davis, I. S., Powers, C. M., Macri, E. M., Hart, H. F., de Oliveira Silva, D., & Crossley, K. M. (2018). 2018 Consensus statement on exercise therapy and physical interventions (orthoses, taping and manual therapy) to treat patellofemoral pain: recommendations from the 5th International Patellofemoral Pain Research Retreat, Gold Coast, Australia, 2017. British journal of sports medicine, 52(18), 1170-1178. https://doi.org/10.1136/bjsports-2018-099397
39. Collins, N. J., Bisset, L. M., Crossley, K. M., & Vicenzino, B. (2012). Efficacy of nonsurgical interventions for anterior knee pain: systematic review and meta-analysis of randomized trials. Sports medicine (Auckland, N.Z.), 42(1), 31-49. https://doi.org/10.2165/11594460-000000000-00000
40. Collins, N. J., van der Heijden, R. A., Macri, E. M., de Kanter, J. L., Oei, E. H. G., Crossley, K. M., Bierma-Zeinstra, S. M. A., & van Middelkoop, M. (2021). Patellofemoral alignment, morphology and structural features are not related to sitting pain in individuals with patellofemoral pain. The Knee, 28, 104-109. https://doi.org/10.1016/j.knee.2020.10.009
41. Collins, N. J., Vicenzino, B., van der Heijden, R. A., & van Middelkoop, M. (2016). Pain During Prolonged Sitting Is a Common Problem in Persons With Patellofemoral Pain. The Journal of orthopaedic and sports physical therapy, 46(8), 658-663. https://doi.org/10.2519/jospt.2016.6470
42. Costa, P. M. P. J. S. B. F. C. J. D. J. F. J. T. (2022). Patellofemoral Pain Syndrome Risk Associated with Squats. International Journal of Environmental Research and Public Health, Vol 19, Iss 9241, p, 9241(eng).
43. Cowan, S. M., Crossley, K. M., & Bennell, K. L. (2009). Altered hip and trunk muscle function in individuals with patellofemoral pain. British journal of sports medicine, 43(8), 584-588. https://doi.org/10.1136/bjsm.2008.053553
44. Cronström, A., Creaby, M. W., Nae, J., & Ageberg, E. (2016). Modifiable Factors Associated with Knee Abduction During Weight-Bearing Activities: A Systematic Review and Meta-Analysis. Sports medicine (Auckland, N.Z.), 46(11), 1647-1662. https://doi.org/10.1007/s40279-016-0519-8
45. Crossley, K. M., van Middelkoop, M., Callaghan, M. J., Collins, N. J., Rathleff, M. S., & Barton, C. J. (2016). 2016 Patellofemoral pain consensus statement from the 4th International Patellofemoral Pain Research Retreat, Manchester. Part 2: recommended physical interventions (exercise, taping, bracing, foot orthoses and combined interventions). British journal of sports medicine, 50(14), 844-852. https://doi.org/10.1136/bjsports-2016-096268

46. *da Silva Boitrago, M. V., de Mello, N. N., Barin, F. R., Júnior, P. L., de Souza Borges, J. H., & Oliveira, M. (2021). Effects of proprioceptive exercises and strengthening on pain and functionality for patellofemoral pain syndrome in women: A randomized controlled trial. Journal of clinical orthopaedics and trauma, 18, 94-99. https://doi.org/10.1016/j.jcot.2021.04.017*
47. *de Moura Campos Carvalho-E-Silva, A. P., Peixoto Leão Almeida, G., Oliveira Magalhães, M., Renovato França, F. J., Vidal Ramos, L. A., Comachio, J., & Pasqual Marques, A. (2016). Dynamic postural stability and muscle strength in patellofemoral pain: Is there a correlation? The Knee, 23(4), 616-621. https://doi.org/10.1016/j.knee.2016.04.013*
48. *de Oliveira Silva, D., Barton, C. J., Briani, R. V., Taborda, B., Ferreira, A. S., Pazzinatto, M. F., & Azevedo, F. M. d. (2019). Kinesiophobia, but not strength is associated with altered movement in women with patellofemoral pain. Gait & posture, 68, 1-5. https://doi.org/10.1016/j.gaitpost.2018.10.033*
49. *De Oliveira Silva, D., Pazzinatto, M. F., Crossley, K. M., Azevedo, F. M., & Barton, C. J. (2020). Novel Stepped Care Approach to Provide Education and Exercise Therapy for Patellofemoral Pain: Feasibility Study. Journal of Medical Internet Research, 22(7), e18584. https://doi.org/10.2196/18584*
50. *de Oliveira Silva, D., Pazzinatto, M. F., Rathleff, M. S., Holden, S., Bell, E., Azevedo, F., & Barton, C. (2020). Patient Education for Patellofemoral Pain: A Systematic Review. The Journal of orthopaedic and sports physical therapy, 50(7), 388-396. https://doi.org/10.2519/jospt.2020.9400*
51. *De Oliveira Silva, D., Willy, R. W., Barton, C. J., Christensen, K., Pazzinatto, M. F., & Azevedo, F. M. (2020). Pain and disability in women with patellofemoral pain relate to kinesiophobia, but not to patellofemoral joint loading variables. Scandinavian journal of medicine & science in sports, 30(11), 2215-2221. https://doi.org/10.1111/sms.13767*
52. *De Oliveira Silva, D. P., Marcella F ; Crossley, Kay M ; Azevedo, Fabio M ; Barton, Christian J. (2020). Novel Stepped Care Approach to Provide Education and Exercise Therapy for Patellofemoral Pain. Journal of Medical Internet Research, Vol 22, Iss 7, p e, 18584(eng).*
53. *Dischiavi, S. L., Wright, A. A., Tarara, D. T., & Bleakley, C. M. (2021). Do exercises for patellofemoral pain reflect common injury mechanisms? A systematic review. Journal of science and medicine in sport, 24(3), 229-240. https://doi.org/10.1016/j.jsams.2020.09.001*
54. *Dolak, K. L., Silkman, C., Medina McKeon, J., Hosey, R. G., Lattermann, C., & Uhl, T. L. (2011). Hip strengthening prior to functional exercises reduces pain sooner than quadriceps strengthening in females with patellofemoral pain syndrome: a randomized clinical trial. The Journal of orthopaedic and sports physical therapy, 41(8), 560-570. https://doi.org/10.2519/jospt.2011.3499*
55. *Dong, C., Li, M., Hao, K., Zhao, C., Piao, K., Lin, W., Fan, C., Niu, Y., & Fei, W. (2021). Dose atrophy of vastus medialis obliquus and vastus lateralis exist in patients with patellofemoral pain syndrome. Journal of orthopaedic surgery and research, 16(1), 128. https://doi.org/10.1186/s13018-021-02251-6*
56. *Dos Santos, A. F., Nakagawa, T. H., Lessi, G. C., Luz, B. C., Matsuo, H. T. M., Nakashima, G. Y., Maciel, C. D., & Serrão, F. V. (2019). Effects of three gait retraining techniques in runners with patellofemoral pain. Physical therapy in sport : official journal of the Association of Chartered Physiotherapists in Sports Medicine, 36, 92-100. https://doi.org/10.1016/j.ptsp.2019.01.006*
57. *DS, K. S. O. L. T. H. (2011). A systematic review of randomized controlled trials on exercise parameters in the treatment of patellofemoral pain. Journal of Multidisciplinary Healthcare, Vol 2011, Iss default, Pp 383-, 392(eng).*
58. *Ducatti, M. H. M., Waiteman, M. C., Botta, A. F. B., Lopes, H. D. S., Glaviano, N. R., Azevedo, F. M. d., & Briani, R. V. (2021). Knee flexor strength, rate of torque development and flexibility in women and men with patellofemoral pain: Relationship with pain and the performance in the single leg bridge test. Physical therapy in sport : official journal of the Association of Chartered Physiotherapists in Sports Medicine, 50, 166-172. https://doi.org/10.1016/j.ptsp.2021.05.006*
59. *Dutton, R. A., Khadavi, M. J., & Fredericson, M. (2014). Update on rehabilitation of patellofemoral pain. Current sports medicine reports, 13(3), 172-178. https://doi.org/10.1249/JSR.0000000000000056*
60. *Earl, J. E., & Hoch, A. Z. (2011). A proximal strengthening program improves pain, function, and biomechanics in women with patellofemoral pain syndrome. The American journal of sports medicine, 39(1), 154-163. https://doi.org/10.1177/0363546510379967*
61. *Earl-Boehm, J. E., Bolgla, L. A., Emory, C., Hamstra-Wright, K. L., Tarima, S., & Ferber, R. (2018). Treatment Success of Hip and Core or Knee Strengthening for Patellofemoral Pain: Development of Clinical Prediction Rules. Journal of athletic training, 53(6), 545-552. https://doi.org/10.4085/1062-6050-510-16*

62. *Emamvirdi, M., Letafatkar, A., & Khaleghi Tazji, M. (2019). The Effect of Valgus Control Instruction Exercises on Pain, Strength, and Functionality in Active Females With Patellofemoral Pain Syndrome. Sports Health, 11(3), 223-237. https://doi.org/10.1177/1941738119837622*
63. *Escamilla, R., Zheng, N., MacLeod, T. D., Imamura, R., Wilk, K. E., Wang, S., Rubenstein, I., Yamashiro, K., & Fleisig, G. S. (2022). Patellofemoral Joint Loading During the Performance of the Forward and Side Lunge with Step Height Variations. International journal of sports physical therapy, 17(2), 174-184. https://doi.org/10.26603/001c.31876*
64. *Esculier, J.-F., Bouyer, L. J., Dubois, B., Fremont, P., Moore, L., McFadyen, B., & Roy, J.-S. (2018). Is combining gait retraining or an exercise programme with education better than education alone in treating runners with patellofemoral pain?A randomised clinical trial. British journal of sports medicine, 52(10), 659-666. https://doi.org/10.1136/bjsports-2016-096988*
65. *Esculier, J.-F., Maggs, K., Maggs, E., & Dubois, B. (2020). A Contemporary Approach to Patellofemoral Pain in Runners. Journal of athletic training, 55(12), 0. https://doi.org/10.4085/1062-6050-0535.19*
66. *Espejo-Antunez, M. A.-C. C. J. B.-Q. A. M. B.-Q. I. E.-P. M. d. I. A. C.-D. L. (2021). Effectiveness of Tele-Prescription of Therapeutic Physical Exercise in Patellofemoral Pain Syndrome during the COVID-19 Pandemic. International Journal of Environmental Research and Public Health, Vol 18, Iss 1048, p, 1048(eng).*
67. *Espejo-Antúnez, M. A.-C. A. J. I.-V. C. J. B.-Q. I. C. L.-P. M. d. I. Á. C.-D. L. (2023). Effects of Radiofrequency Diathermy Plus Therapeutic Exercises on Pain and Functionality of Patients with Patellofemoral Pain Syndrome. Journal of Clinical Medicine, Vol 12, Iss 2348, p, 2348(eng).*
68. *Faller, B., Bonneau, D., Wooten, L., & Jayaseelan, D. J. (2021). Eccentric exercise in the prevention of patellofemoral pain in high-volume runners: A rationale for integration. Sports medicine and health science, 3(2), 119-124. https://doi.org/10.1016/j.smhs.2021.04.003*
69. *Felicio, L. R., de Carvalho, C. A. M., Dias, C. L. C. A., & Vigário, P. D. S. (2019). Electromyographic activity of the quadriceps and gluteus medius muscles during/different straight leg raise and squat exercises in women with patellofemoral pain syndrome. Journal of electromyography and kinesiology : official journal of the International Society of Electrophysiological Kinesiology, 48, 17-23. https://doi.org/10.1016/j.jelekin.2019.05.017*
70. *Felicio, L. R., Saad, M. C., Liporaci, R. F., Baffa, A. d. P., dos Santos, A. C., & Bevilaqua-Grossi, D. (2012). Correlation between trochlear groove depth and patellar position during open and closed kinetic chain exercises in subjects with anterior knee pain. Journal of applied biomechanics, 28(3), 335-342. https://doi.org/10.1123/jab.28.3.335*
71. *Ferber, R., Bolgla, L., Earl-Boehm, J. E., Emery, C., & Hamstra-Wright, K. (2015). Strengthening of the hip and core versus knee muscles for the treatment of patellofemoral pain: a multicenter randomized controlled trial. Journal of athletic training, 50(4), 366-377. https://doi.org/10.4085/1062-6050-49.3.70*
72. *Ferber, R., Kendall, K. D., & Farr, L. (2011). Changes in knee biomechanics after a hip-abductor strengthening protocol for runners with patellofemoral pain syndrome. Journal of athletic training, 46(2), 142-149. https://doi.org/10.4085/1062-6050-46.2.142*
73. *Ferreira, A. S., de Oliveira Silva, D., Ferrari, D., Magalhães, F. H., Pappas, E., Briani, R. V., Pazzinatto, M. F., & de Azevedo, F. M. (2021). Knee and Hip Isometric Force Steadiness Are Impaired in Women With Patellofemoral Pain. Journal of strength and conditioning research, 35(10), 2878-2885. https://doi.org/10.1519/JSC.0000000000003215*
74. *Ford, K. R., Nguyen, A.-D., Dischiavi, S. L., Hegedus, E. J., Zuk, E. F., & Taylor, J. B. (2015). An evidence-based review of hip-focused neuromuscular exercise interventions to address dynamic lower extremity valgus. Open Access Journal of Sports Medicine, 6, 291-303. https://doi.org/10.2147/OAJSM.S72432*
75. *Foroughi, F., Sobhani, S., Yoosefinejad, A. K., & Motealleh, A. (2019). Added Value of Isolated Core Postural Control Training on Knee Pain and Function in Women With Patellofemoral Pain Syndrome: A Randomized Controlled Trial. Archives of physical medicine and rehabilitation, 100(2), 220-229. https://doi.org/10.1016/j.apmr.2018.08.180*
76. *Freeman, A. J., Jacobson, N. A., & Fogg, Q. A. (2008). Anatomical variations of the plantaris muscle and a potential role in patellofemoral pain syndrome. Clinical anatomy (New York, N.Y.), 21(2), 178-181. https://doi.org/10.1002/ca.20594*
77. *Frye, J. L., Ramey, L. N., & Hart, J. M. (2012). The effects of exercise on decreasing pain and increasing function in patients with patellofemoral pain syndrome: a systematic review. Sports Health, 4(3), 205-210. https://doi.org/10.1177/1941738112441915*

78. *Fu, C. Y. S. X. Y. Y. X. Z. J. W. W. (2019). Patellofemoral Joint Loads during Running Immediately Changed by Shoes with Different Minimalist Indices. Applied Sciences, Vol 9, Iss 19, p, 4176(eng).*
79. *Fukuda, T. Y., Melo, W. P., Zaffalon, B. M., Rossetto, F. M., Magalhães, E., Bryk, F. F., & Martin, R. L. (2012). Hip posterolateral musculature strengthening in sedentary women with patellofemoral pain syndrome: a randomized controlled clinical trial with 1-year follow-up. The Journal of orthopaedic and sports physical therapy, 42(10), 823-830. https://doi.org/10.2519/jospt.2012.4184*
80. *Fukuda, T. Y., Rossetto, F. M., Magalhães, E., Bryk, F. F., Lucareli, P. R. G., & de Almeida Aparecida Carvalho, N. (2010). Short-term effects of hip abductors and lateral rotators strengthening in females with patellofemoral pain syndrome: a randomized controlled clinical trial. The Journal of orthopaedic and sports physical therapy, 40(11), 736-742. https://doi.org/10.2519/jospt.2010.3246*
81. *Gandomi, A. Y. M. A. F. (2020). The Effects of 12-Weeks of Sensorimotor Exercise on Pain, Strength, Pelvic Drop, and Dynamic Knee Valgus in Males With Patellofemoral Pain Syndrome. Physical Treatments, Vol 10, Iss 3, Pp 159-, 168(eng).*
82. *Giles, L. S., Webster, K. E., McClelland, J. A., & Cook, J. (2013). Does quadriceps atrophy exist in individuals with patellofemoral pain? A systematic literature review with meta-analysis. The Journal of orthopaedic and sports physical therapy, 43(11), 766-776. https://doi.org/10.2519/jospt.2013.4833*
83. *Glaviano, N. R., Bazett-Jones, D. M., & Norte, G. (2019). Gluteal muscle inhibition: Consequences of patellofemoral pain? Medical hypotheses, 126, 9-14. https://doi.org/10.1016/j.mehy.2019.02.046*
84. *Glaviano, N. R., & Saliba, S. (2016). Impairment based rehabilitation for patellofemoral pain patients. The Physician and sportsmedicine, 44(3), 311-323. https://doi.org/10.1080/00913847.2016.1200443*
85. *Glaviano, N. R., & Saliba, S. (2018). Relationship Between Lower-Extremity Strength and Subjective Function in Individuals With Patellofemoral Pain. Journal of sport rehabilitation, 27(4), 327-333. https://doi.org/10.1123/jsr.2016-0177*
86. *Goulette, D., Griffith, P., Schiller, M., Rutherford, D., & Kernozek, T. W. (2021). Patellofemoral joint loading during the forward and backward lunge. Physical therapy in sport : official journal of the Association of Chartered Physiotherapists in Sports Medicine, 47, 178-184. https://doi.org/10.1016/j.ptsp.2020.12.001*
87. *Greaves, H., Comfort, P., Liu, A., Herrington, L., & Jones, R. (2021). How effective is an evidence-based exercise intervention in individuals with patellofemoral pain? Physical therapy in sport : official journal of the Association of Chartered Physiotherapists in Sports Medicine, 51, 92-101. https://doi.org/10.1016/j.ptsp.2021.05.013*
88. *Gross, C. (2016). Effektivität von Übungen zur Kräftigung der Hüftmuskulatur bei Patienten mit patellofemoralem Schmerzsyndrom. manuelletherapie, 20(03), 137-148.*
89. *Halabchi, F., Mazaheri, R., Mansournia, M. A., & Hamedi, Z. (2015). Additional Effects of an Individualized Risk Factor-Based Approach on Pain and the Function of Patients With Patellofemoral Pain Syndrome: A Randomized Controlled Trial. Clinical journal of sport medicine : official journal of the Canadian Academy of Sport Medicine, 25(6), 478-486. https://doi.org/10.1097/JSM.0000000000000177*
90. *Halabchi, F., Mazaheri, R., & Seif-Barghi, T. (2013). Patellofemoral pain syndrome and modifiable intrinsic risk factors; how to assess and address? Asian journal of sports medicine, 4(2), 85-100. https://doi.org/10.5812/asjsm.34488*
91. *Hamstra-Wright, K. L., Aydemir, B., Earl-Boehm, J., Bolgla, L., Emery, C., & Ferber, R. (2017). Lasting Improvement of Patient-Reported Outcomes 6 Months After Patellofemoral Pain Rehabilitation. Journal of sport rehabilitation, 26(4), 223-233. https://doi.org/10.1123/jsr.2015-0176*
92. *Hansen, R., Brushøj, C., Rathleff, M. S., Magnusson, S. P., & Henriksen, M. (2023). Quadriceps or hip exercises for patellofemoral pain? A randomised controlled equivalence trial. British journal of sports medicine, bjsports-2022-106197. https://doi.org/10.1136/bjsports-2022-106197*
93. *Hart, H. F., Patterson, B. E., Crossley, K. M., Culvenor, A. G., Khan, M. C. M., King, M. G., & Sritharan, P. (2022). May the force be with you: understanding how patellofemoral joint reaction force compares across different activities and physical interventions-a systematic review and meta-analysis. British journal of sports medicine, 56(9), 521-530. https://doi.org/10.1136/bjsports-2021-104686*
94. *Harvie, D., O'Leary, T., & Kumar, S. (2011). A systematic review of randomized controlled trials on exercise parameters in the treatment of patellofemoral pain: what works? Journal of multidisciplinary healthcare, 4, 383-392. https://doi.org/10.2147/JMDH.S24595*

95. Hasan, S. F. F. L. S. S. A. F. R. K. (2022). Isometric & short arc exercises effect on the quadricep muscle of patello-femoral knee pain patients. International Journal of Endorsing Health Science Research, Vol 9, Iss, 4(eng).
96. Hatefi, F. B. M. H. M. (2020). Effects of Total Resistance Exercise versus Physioball Exercise Training Programs on Pain and Functional Disability among Women with Patellofemoral Pain. Women_s Health Bulletin, Vol 7, Iss 3, Pp 27-, 35(eng).
97. Hiemstra, L. A., Kerslake, S., & Arendt, E. A. (2017). Clinical Rehabilitation of Anterior Knee Pain: Current Concepts. American journal of orthopedics (Belle Mead, N.J.), 46(2), 82-86.
98. Hoglund, L. T., Burns, R. O., & Stepney, A. L. J. (2018). DO MALES WITH PATELLOFEMORAL PAIN HAVE POSTEROLATERAL HIP MUSCLE WEAKNESS? International journal of sports physical therapy, 13(2), 160-170. https://www.ncbi.nlm.nih.gov/pmc/articles/PMC6063054/pdf/ijspt-13-160.pdf
99. Holden, S., Matthews, M., Rathleff, M. S., Kasza, J., & Vicenzino, B. (2021). How Do Hip Exercises Improve Pain in Individuals With Patellofemoral Pain? Secondary Mediation Analysis of Strength and Psychological Factors as Mechanisms. The Journal of orthopaedic and sports physical therapy, 51(12), 602-610. https://doi.org/10.2519/jospt.2021.10674
100. Hott, A., Brox, J. I., Pripp, A. H., Juel, N. G., & Liavaag, S. (2020). Patellofemoral pain: One year results of a randomized trial comparing hip exercise, knee exercise, or free activity. Scandinavian journal of medicine & science in sports, 30(4), 741-753. https://doi.org/10.1111/sms.13613
101. Hott, A., Brox, J. I., Pripp, A. H., Juel, N. G., Paulsen, G., & Liavaag, S. (2019). Effectiveness of Isolated Hip Exercise, Knee Exercise, or Free Physical Activity for Patellofemoral Pain: A Randomized Controlled Trial. The American journal of sports medicine, 47(6), 1312-1322. https://doi.org/10.1177/0363546519830644
102. Irish, S. E., Millward, A. J., Wride, J., Haas, B. M., & Shum, G. L. K. (2010). The effect of closed-kinetic chain exercises and open-kinetic chain exercise on the muscle activity of vastus medialis oblique and vastus lateralis. Journal of strength and conditioning research, 24(5), 1256-1262. https://doi.org/10.1519/JSC.0b013e3181cf749f
103. Ismail, M. M., Gamaleldein, M. H., & Hassa, K. A. (2013). Closed kinetic chain exercises with or without additional hip strengthening exercises in management of patellofemoral pain syndrome: a randomized controlled trial. European journal of physical and rehabilitation medicine, 49(5), 687-698.
104. Jang, J. H. L. K. H. S. G. B. L. S. S. K.-M. (2023). Comparison of Functional Outcomes between Supervised Rehabilitation and Telerehabilitation in Female Patients with Patellofemoral Pain Syndrome during the COVID-19 Pandemic. International Journal of Environmental Research and Public Health, Vol 20, Iss 2233, p, 2233(eng).
105. Jellad, A., Kalai, A., Guedria, M., Jguirim, M., Elmhamdi, S., Salah, S., & Frih, Z. B. S. (2021). Combined Hip Abductor and External Rotator Strengthening and Hip Internal Rotator Stretching Improves Pain and Function in Patients With Patellofemoral Pain Syndrome: A Randomized Controlled Trial With Crossover Design. Orthopaedic journal of sports medicine, 9(4), 2325967121989729. https://doi.org/10.1177/2325967121989729
106. Kalra, R. M. J. Y. S. (2016). THE EFFECT OF ADDING SPECIFIC HIP STRENGTHENING EXERCISES TO CONVENTIONAL KNEE EXERCISES IN PATIENTS WITH PATELLO FEMORAL PAIN SYNDROME. International Journal of Physiotherapy, Vol 3, Iss, 1(eng).
107. Karolak, J. W. s. P. a. K. J. (2015). The influence of isometric exercises of the quadriceps muscle on young female patients with anterior knee pain. Studia Medyczne, Vol 31, Iss 3, Pp 183-, 186(eng).
108. Kaya, D., Citaker, S., Kerimoglu, U., Atay, O. A., Nyland, J., Callaghan, M., Yakut, Y., Yüksel, I., & Doral, M. N. (2011). Women with patellofemoral pain syndrome have quadriceps femoris volume and strength deficiency. Knee surgery, sports traumatology, arthroscopy : official journal of the ESSKA, 19(2), 242-247. https://doi.org/10.1007/s00167-010-1290-2
109. Kaya, D., Doral, M. N., & Callaghan, M. (2012). How can we strengthen the quadriceps femoris in patients with patellofemoral pain syndrome? Muscles, Ligaments and Tendons Journal, 2(1), 25-32. https://www.ncbi.nlm.nih.gov/pmc/articles/PMC3666499/pdf/mltj_1-2012_pag_25-32.pdf
110. Keays, S. L., Mason, M., & Newcombe, P. A. (2015). Individualized physiotherapy in the treatment of patellofemoral pain. Physiotherapy research international : the journal for researchers and clinicians in physical therapy, 20(1), 22-36. https://doi.org/10.1002/pri.1593

111. Keays, S. L., Mason, M., & Newcombe, P. A. (2016). Three-Year Outcome After a 1-Month Physiotherapy Program of Local and Individualized Global Treatment for Patellofemoral Pain Followed by Self-Management. Clinical journal of sport medicine : official journal of the Canadian Academy of Sport Medicine, 26(3), 190-198. https://doi.org/10.1097/JSM.0000000000000226
112. Kernozek, T. W., Gheidi, N., Zellmer, M., Hove, J., Heinert, B. L., & Torry, M. R. (2018). Effects of Anterior Knee Displacement During Squatting on Patellofemoral Joint Stress. Journal of sport rehabilitation, 27(3), 237-243. https://doi.org/10.1123/jsr.2016-0197
113. Khayambashi, K., Fallah, A., Movahedi, A., Bagwell, J., & Powers, C. (2014). Posterolateral hip muscle strengthening versus quadriceps strengthening for patellofemoral pain: a comparative control trial. Archives of physical medicine and rehabilitation, 95(5), 900-907. https://doi.org/10.1016/j.apmr.2013.12.022
114. Khayambashi, K., Mohammadkhani, Z., Ghaznavi, K., Lyle, M. A., & Powers, C. M. (2012). The effects of isolated hip abductor and external rotator muscle strengthening on pain, health status, and hip strength in females with patellofemoral pain: a randomized controlled trial. The Journal of orthopaedic and sports physical therapy, 42(1), 22-29. https://doi.org/10.2519/jospt.2012.3704
115. Kim, H.-J., Cho, J., & Lee, S. (2022). Talonavicular joint mobilization and foot core strengthening in patellofemoral pain syndrome: a single-blind, three-armed randomized controlled trial. BMC musculoskeletal disorders, 23(1), 150. https://doi.org/10.1186/s12891-022-05099-x
116. Kim, T. W. B., Gay, N., Khemka, A., & Garino, J. (2016). Internet-Based Exercise Therapy Using Algorithms for Conservative Treatment of Anterior Knee Pain: A Pragmatic Randomized Controlled Trial. JMIR rehabilitation and assistive technologies, 3(2), e12. https://doi.org/10.2196/rehab.5148
117. Kısacık, P., Tunay, V. B., Bek, N., Atay, Ö. A., Selfe, J., & Karaduman, A. A. (2021). Short foot exercises have additional effects on knee pain, foot biomechanics, and lower extremity muscle strength in patients with patellofemoral pain. Journal of back and musculoskeletal rehabilitation, 34(6), 1093-1104. https://doi.org/10.3233/BMR-200255
118. Knobloch, K., Yoon, U., & Vogt, P. M. (2008). Acute and overuse injuries correlated to hours of training in master running athletes. Foot & Ankle International, 29(7), 671-676. https://doi.org/10.3113/FAI.2008.0671
119. Kölle, T., Alt, W., & Wagner, D. (2020). Effects of a 12-week home exercise therapy program on pain and neuromuscular activity in patients with patellofemoral pain syndrome. Archives of orthopaedic and trauma surgery, 140(12), 1985-1992. https://doi.org/10.1007/s00402-020-03543-y
120. Kooiker, L., Van De Port, I. G. L., Weir, A., & Moen, M. H. (2014). Effects of physical therapist-guided quadriceps-strengthening exercises for the treatment of patellofemoral pain syndrome: a systematic review. The Journal of orthopaedic and sports physical therapy, 44(6), 391-402, B391. https://doi.org/10.2519/jospt.2014.4127
121. Kunene, S. H., Taukobong, N. P., & Ramklass, S. (2020). Rehabilitation approaches to anterior knee pain among runners: A scoping review. The South African journal of physiotherapy, 76(1), 1342. https://doi.org/10.4102/sajp.v76i1.1342
122. Kuriki, K. M. M. e. L. J. d. S. F. R. I. B. A. M. M. M. G. d. A. D. C. d. S. H. U. (2020). Effects of a 12-week hip abduction exercise program on the electromyographic activity of hip and knee muscles of women with patellofemoral pain. Motriz: Revista de Educacao Fisica, Vol 26, Iss, 1(eng).
123. Lack, S. (2018). The interaction of hip and foot biomechanics in the presentation and management of patellofemoral pain. British journal of sports medicine, 52(8), 544-545. https://doi.org/10.1136/bjsports-2017-098241
124. Lack, S., Barton, C., Sohan, O., Crossley, K., & Morrissey, D. (2015). Proximal muscle rehabilitation is effective for patellofemoral pain: a systematic review with meta-analysis. British journal of sports medicine, 49(21), 1365-1376. https://doi.org/10.1136/bjsports-2015-094723
125. Lack, S., Neal, B., De Oliveira Silva, D., & Barton, C. (2018). How to manage patellofemoral pain - Understanding the multifactorial nature and treatment options. Physical therapy in sport : official journal of the Association of Chartered Physiotherapists in Sports Medicine, 32, 155-166. https://doi.org/10.1016/j.ptsp.2018.04.010
126. Lankhorst, N. E., van Middelkoop, M., van Trier, Y. D. M., van Linschoten, R., Koes, B. W., Verhaar, J. A. N., & Bierma-Zeinstra, S. M. A. (2015). Can we predict which patients with patellofemoral pain are more likely to benefit from exercise therapy? A secondary exploratory analysis of a randomized controlled trial. The Journal of orthopaedic and sports physical therapy, 45(3), 183-189. https://doi.org/10.2519/jospt.2015.5583

127. Lee, J., Yoon, J., & Cynn, H. (2017). Foot exercise and taping in patients with patellofemoral pain and pronated foot. Journal of bodywork and movement therapies, 21(1), 216-222. https://doi.org/10.1016/j.jbmt.2016.07.010
128. Lee, J. H., Jang, K.-M., Kim, E., Rhim, H. C., & Kim, H.-D. (2021). Static and Dynamic Quadriceps Stretching Exercises in Patients With Patellofemoral Pain: A Randomized Controlled Trial. Sports Health, 13(5), 482-489. https://doi.org/10.1177/1941738121993777
129. Lee, J. H., Jang, K.-M., Kim, E., Rhim, H. C., & Kim, H.-D. (2021). Effects of Static and Dynamic Stretching With Strengthening Exercises in Patients With Patellofemoral Pain Who Have Inflexible Hamstrings: A Randomized Controlled Trial. Sports Health, 13(1), 49-56. https://doi.org/10.1177/1941738120932911
130. Lee, J. H., Shin, K. H., Han, S.-B., Sun Hwang, K., Lee, S. J., & Jang, K.-M. (2022). Prospective comparative study between knee alignment-oriented static and dynamic balance exercise in patellofemoral pain syndrome patients with dynamic knee valgus. Medicine, 101(37), e30631. https://doi.org/10.1097/MD.0000000000030631
131. Lee, J. H., Shin, K. H., Lee, G. B., Son, S., & Jang, K.-M. (2023). Comparison of Functional Outcomes between Supervised Rehabilitation and Telerehabilitation in Female Patients with Patellofemoral Pain Syndrome during the COVID-19 Pandemic. International journal of environmental research and public health, 20(3). https://doi.org/10.3390/ijerph20032233
132. Lenhart, R. L., Smith, C. R., Vignos, M. F., Kaiser, J., Heiderscheit, B. C., & Thelen, D. G. (2015). Influence of step rate and quadriceps load distribution on patellofemoral cartilage contact pressures during running. Journal of biomechanics, 48(11), 2871-2878. https://doi.org/10.1016/j.jbiomech.2015.04.036
133. Liang, J. N., Budge, S., Madriaga, A., Meske, K., Nguyenton, D., & Ho, K.-Y. (2021). Neurophysiological changes of brain and spinal cord in individuals with patellofemoral pain: a systematic review and meta-analysis protocol. BMJ Open, 11(7), e049882. https://doi.org/10.1136/bmjopen-2021-049882
134. Luz, B. C., Dos Santos, A. F., & Serrão, F. V. (2021). Are hip and knee kinematics and training load characteristics relate to pain intensity and physical function level in runners with Patellofemoral Pain? Gait & posture, 84, 162-168. https://doi.org/10.1016/j.gaitpost.2020.11.027
135. Magalhães, E., Fukuda, T. Y., Sacramento, S. N., Forgas, A., Cohen, M., & Abdalla, R. J. (2010). A comparison of hip strength between sedentary females with and without patellofemoral pain syndrome. The Journal of orthopaedic and sports physical therapy, 40(10), 641-647. https://doi.org/10.2519/jospt.2010.3120
136. Magalhães, E., Silva, A. P. M. C. C., Sacramento, S. N., Martin, R. L., & Fukuda, T. Y. (2013). Isometric strength ratios of the hip musculature in females with patellofemoral pain: a comparison to pain-free controls. Journal of strength and conditioning research, 27(8), 2165-2170. https://doi.org/10.1519/JSC.0b013e318279793d
137. Manojlović, D., Kozinc, Ž., & Šarabon, N. (2021). Trunk, Hip and Knee Exercise Programs for Pain Relief, Functional Performance and Muscle Strength in Patellofemoral Pain: Systematic Review and Meta-Analysis. Journal of Pain Research, 14, 1431-1449. https://doi.org/10.2147/JPR.S301448
138. McClinton, S. M., Cobian, D. G., & Heiderscheit, B. C. (2020). Physical Therapist Management of Anterior Knee Pain. Current Reviews in Musculoskeletal Medicine, 13(6), 776-787. https://doi.org/10.1007/s12178-020-09678-0
139. Moghadam, Z. A., AE ; Javaheri, SAH. (2016). Comparing the Effect of Open and Closed Kinetic Chain Exercises in Patients Suffering From Patellofemoral Pain Syndrome. International Journal of Basic Science in Medicine, Vol 1, Iss 2, Pp 53-, 57(eng).
140. Mølgaard, C. M., Rathleff, M. S., Andreasen, J., Christensen, M., Lundbye-Christensen, S., Simonsen, O., & Kaalund, S. (2018). Foot exercises and foot orthoses are more effective than knee focused exercises in individuals with patellofemoral pain. Journal of science and medicine in sport, 21(1), 10-15. https://doi.org/10.1016/j.jsams.2017.05.019
141. Motealleh, A., Mohamadi, M., Moghadam, M. B., Nejati, N., Arjang, N., & Ebrahimi, N. (2019). Effects of Core Neuromuscular Training on Pain, Balance, and Functional Performance in Women With Patellofemoral Pain Syndrome: A Clinical Trial. Journal of Chiropractic Medicine, 18(1), 9-18. https://doi.org/10.1016/j.jcm.2018.07.006
142. Moyano, F. R., Valenza, M. C., Martin, L. M., Caballero, Y. C., Gonzalez-Jimenez, E., & Demet, G. V. (2013). Effectiveness of different exercises and stretching physiotherapy on pain and movement in patellofemoral pain syndrome: a randomized controlled trial. Clinical rehabilitation, 27(5), 409-417. https://doi.org/10.1177/0269215512459277

143. Muniz, A. M. d. S., Zeitoune, G., Alvim, F., Grassi, G. B. A., Britto, P. A. A., & Nadal, J. (2023). Do exist differences in kinematics and EMG of the hip and knee between male runners with and without patellofemoral pain in different running speeds? Physical therapy in sport : official journal of the Association of Chartered Physiotherapists in Sports Medicine, 59, 122-129. https://doi.org/10.1016/j.ptsp.2022.12.006
144. Na, Y., Han, C., Shi, Y., Zhu, Y., Ren, Y., & Liu, W. (2021). Is Isolated Hip Strengthening or Traditional Knee-Based Strengthening More Effective in Patients With Patellofemoral Pain Syndrome? A Systematic Review With Meta-analysis. Orthopaedic journal of sports medicine, 9(7), 23259671211017503. https://doi.org/10.1177/23259671211017503
145. Nascimento, L. R., Teixeira-Salmela, L. F., Souza, R. B., & Resende, R. A. (2018). Hip and Knee Strengthening Is More Effective Than Knee Strengthening Alone for Reducing Pain and Improving Activity in Individuals With Patellofemoral Pain: A Systematic Review With Meta-analysis. The Journal of orthopaedic and sports physical therapy, 48(1), 19-31. https://doi.org/10.2519/jospt.2018.7365
146. Neal, B. S., Bartholomew, C., Barton, C. J., Morrissey, D., & Lack, S. D. (2022). Six Treatments Have Positive Effects at 3 Months for People With Patellofemoral Pain: A Systematic Review With Meta-analysis. The Journal of orthopaedic and sports physical therapy, 52(11), 750-768. https://doi.org/10.2519/jospt.2022.11359
147. Neal, B. S., Barton, C. J., Gallie, R., O'Halloran, P., & Morrissey, D. (2016). Runners with patellofemoral pain have altered biomechanics which targeted interventions can modify: A systematic review and meta-analysis. Gait & posture, 45, 69-82. https://doi.org/10.1016/j.gaitpost.2015.11.018
148. Nielsen, T. G., Miller, L. L., Mygind-Klavsen, B., & Lind, M. (2020). A simple rehabilitation regime improves functional outcome in patients with patellafemoral pain after 12 month. Journal of experimental orthopaedics, 7(1), 5. https://doi.org/10.1186/s40634-020-00223-z
149. Nunes, G. S., Rodrigues, D. Z., Hörbe, L., Prates, I., Tessarin, B. M., Serrão, F. V., & de Noronha, M. (2022). Is Postural Control Affected in People with Patellofemoral Pain and Should it be Part of Rehabilitation? A Systematic Review with Meta-analysis. Sports medicine - open, 8(1), 144. https://doi.org/10.1186/s40798-022-00538-4
150. Olesen, M. S. R. C. R. R. S. H. K. T. J. L. (2018). Exercise therapy, patient education, and patellar taping in the treatment of adolescents with patellofemoral pain. Pilot and Feasibility Studies, Vol 4, Iss 1, Pp 1-, 9(eng).
151. Østerås, B., Østerås, H., & Torstensen, T. A. (2013). Long-term effects of medical exercise therapy in patients with patellofemoral pain syndrome: results from a single-blinded randomized controlled trial with 12 months follow-up. Physiotherapy, 99(4), 311-316. https://doi.org/10.1016/j.physio.2013.04.001
152. Pereira, P. M., Baptista, J. S., Conceição, F., Duarte, J., Ferraz, J., & Costa, J. T. (2022). Patellofemoral Pain Syndrome Risk Associated with Squats: A Systematic Review. International journal of environmental research and public health, 19(15). https://doi.org/10.3390/ijerph19159241
153. Peters, J. S. J., & Tyson, N. L. (2013). Proximal exercises are effective in treating patellofemoral pain syndrome: a systematic review. International journal of sports physical therapy, 8(5), 689-700. https://www.ncbi.nlm.nih.gov/pmc/articles/PMC3811739/pdf/ijspt-10-689.pdf
154. Piva, S. R., Fitzgerald, G. K., Irrgang, J. J., Fritz, J. M., Wisniewski, S., McGinty, G. T., Childs, J. D., Domenech, M. A., Jones, S., & Delitto, A. (2009). Associates of physical function and pain in patients with patellofemoral pain syndrome. Archives of physical medicine and rehabilitation, 90(2), 285-295. https://doi.org/10.1016/j.apmr.2008.08.214
155. Piva, S. R., Fitzgerald, G. K., Wisniewski, S., & Delitto, A. (2009). Predictors of pain and function outcome after rehabilitation in patients with patellofemoral pain syndrome. Journal of rehabilitation medicine, 41(8), 604-612. https://doi.org/10.2340/16501977-0372
156. Powers, C. M., Ho, K.-Y., Chen, Y.-J., Souza, R. B., & Farrokhi, S. (2014). Patellofemoral joint stress during weight-bearing and non-weight-bearing quadriceps exercises. The Journal of orthopaedic and sports physical therapy, 44(5), 320-327. https://doi.org/10.2519/jospt.2014.4936
157. Prins, M. R., & van der Wurff, P. (2009). Females with patellofemoral pain syndrome have weak hip muscles: a systematic review. The Australian Journal of Physiotherapy, 55(1), 9-15. https://doi.org/10.1016/s0004-9514(09)70055-8
158. Rabelo, N. D. D. A., Costa, L. O. P., Lima, B. M. d., Dos Reis, A. C., Bley, A. S., Fukuda, T. Y., & Lucareli, P. R. G. (2017). Adding motor control training to muscle strengthening did not substantially improve the effects on clinical or kinematic outcomes in women with patellofemoral pain: A randomised controlled trial. Gait & posture, 58, 280-286. https://doi.org/10.1016/j.gaitpost.2017.08.018

159. Rabelo, N. D. D. A., Lima, B., Reis, A. C. d., Bley, A. S., Yi, L. C., Fukuda, T. Y., Costa, L. O. P., & Lucareli, P. R. G. (2014). Neuromuscular training and muscle strengthening in patients with patellofemoral pain syndrome: a protocol of randomized controlled trial. BMC musculoskeletal disorders, 15, 157. https://doi.org/10.1186/1471-2474-15-157
160. Raisi, A. S., S. ; Habibi, R. (2020). The Effect and Durability of Hip and Core Exercises on Pain and Performance in Females With Patellofemoral Pain Syndrome. Physical Treatments, Vol 10, Iss 3, Pp 145-, 158(eng).
161. Ramazzina, I., Pogliacomi, F., Bertuletti, S., & Costantino, C. (2016). Long term effect of selective muscle strengthening in athletes with patellofemoral pain syndrome. Acta bio-medica : Atenei Parmensis, 87 Suppl 1, 60-68.
162. Rasti, E., Rojhani-Shirazi, Z., Ebrahimi, N., & Sobhan, M. R. (2020). Effects of whole body vibration with exercise therapy versus exercise therapy alone on flexibility, vertical jump height, agility and pain in athletes with patellofemoral pain: a randomized clinical trial. BMC musculoskeletal disorders, 21(1), 705. https://doi.org/10.1186/s12891-020-03732-1
163. Rathleff, C. R., Baird, W. N., Olesen, J. L., Roos, E. M., Rasmussen, S., & Rathleff, M. S. (2013). Hip and knee strength is not affected in 12-16 year old adolescents with patellofemoral pain--a cross-sectional population-based study. PLoS One, 8(11), e79153. https://doi.org/10.1371/journal.pone.0079153
164. Rathleff, M. S., Graven-Nielsen, T., Hölmich, P., Winiarski, L., Krommes, K., Holden, S., & Thorborg, K. (2019). Activity Modification and Load Management of Adolescents With Patellofemoral Pain: A Prospective Intervention Study Including 151 Adolescents. The American journal of sports medicine, 47(7), 1629-1637. https://doi.org/10.1177/0363546519843915
165. Rathleff, M. S., Rathleff, C. R., Crossley, K. M., & Barton, C. J. (2014). Is hip strength a risk factor for patellofemoral pain? A systematic review and meta-analysis. British journal of sports medicine, 48(14), 1088. https://doi.org/10.1136/bjsports-2013-093305
166. Rathleff, M. S., Rathleff, C. R., Holden, S., Thorborg, K., & Olesen, J. L. (2018). Exercise therapy, patient education, and patellar taping in the treatment of adolescents with patellofemoral pain: a prospective pilot study with 6 months follow-up. Pilot and feasibility studies, 4, 73. https://doi.org/10.1186/s40814-017-0227-7
167. Rathleff, M. S., Roos, E. M., Olesen, J. L., & Rasmussen, S. (2015). Exercise during school hours when added to patient education improves outcome for 2 years in adolescent patellofemoral pain: a cluster randomised trial. Br J Sports Med, 49(6), 406-412. https://doi.org/10.1136/bjsports-2014-093929
168. Rathleff, M. S., Samani, A., Olesen, J. L., Roos, E. M., Rasmussen, S., Christensen, B. H., & Madeleine, P. (2013). Neuromuscular activity and knee kinematics in adolescents with patellofemoral pain. Medicine and science in sports and exercise, 45(9), 1730-1739. https://doi.org/10.1249/MSS.0b013e318292be30
169. Rhon, J. L. Y. S. J. S. J. A. C. D. I. (2021). Timing of physical therapy for individuals with patellofemoral pain and the influence on healthcare use, costs and recurrence rates. BMC Health Services Research, Vol 21, Iss 1, Pp 1-, 9(eng).
170. Rogan, S., Haehni, M., Luijckx, E., Dealer, J., Reuteler, S., & Taeymans, J. (2019). Effects of Hip Abductor Muscles Exercises on Pain and Function in Patients With Patellofemoral Pain: A Systematic Review and Meta-Analysis. Journal of strength and conditioning research, 33(11), 3174-3187. https://doi.org/10.1519/JSC.0000000000002658
171. Saad, M. C., Felício, L. R., Masullo, C. d. L., Liporaci, R. F., & Bevilaqua-Grossi, D. (2011). Analysis of the center of pressure displacement, ground reaction force and muscular activity during step exercises. Journal of electromyography and kinesiology : official journal of the International Society of Electrophysiological Kinesiology, 21(5), 712-718. https://doi.org/10.1016/j.jelekin.2011.07.014
172. Saad, M. C., Vasconcelos, R. A. d., Mancinelli, L. V. d. O., Munno, M. S. d. B., Liporaci, R. F., & Grossi, D. B. (2018). Is hip strengthening the best treatment option for females with patellofemoral pain? A randomized controlled trial of three different types of exercises. Brazilian Journal of Physical Therapy, 22(5), 408-416. https://doi.org/10.1016/j.bjpt.2018.03.009
173. Sadati, F. P. A. D. S. K. M. (2021). The Effect of Resistance Training on Lower Extremity Pain, Strength and Kinematical Parameters in Women With Patellofemoral Complications. Physical Treatments, Vol 11, Iss 4, Pp 217-, 226(eng).
174. Şahin, M., Ayhan, F. F., Borman, P., & Atasoy, H. (2016). The effect of hip and knee exercises on pain, function, and strength in patientswith patellofemoral pain syndrome: a randomized controlled trial. Turkish journal of medical sciences, 46(2), 265-277. https://doi.org/10.3906/sag-1409-66

175. Saltychev, M., Dutton, R. A., Laimi, K., Beaupré, G. S., Virolainen, P., & Fredericson, M. (2018). Effectiveness of conservative treatment for patellofemoral pain syndrome: A systematic review and meta-analysis. Journal of rehabilitation medicine, 50(5), 393-401. https://doi.org/10.2340/16501977-2295
176. Samani, M., Kordi Yoosefinejad, A., Campos, M. H., de Lira, C. A. B., & Motealleh, A. (2020). Changes in Knee Vastii Muscle Activity in Women with Patellofemoral Pain Syndrome During the Menstrual Cycle. PM & R : the journal of injury, function, and rehabilitation, 12(4), 382-390. https://doi.org/10.1002/pmrj.12239
177. Samual, A. K., Rana, S. H., Adam, L. B., Kim, L. B., Patrick, L. R., & Kade, L. P. (2023). Do biomechanical foot-based interventions reduce patellofemoral joint loads in adults with and without patellofemoral pain or osteoarthritis? A systematic review and meta-analysis. British journal of sports medicine, 57(13), 872. https://doi.org/10.1136/bjsports-2022-106542
178. Santos, T. R. T., Oliveira, B. A., Ocarino, J. M., Holt, K. G., & Fonseca, S. T. (2015). Effectiveness of hip muscle strengthening in patellofemoral pain syndrome patients: a systematic review. Brazilian Journal of Physical Therapy, 19(3), 167-176. https://doi.org/10.1590/bjpt-rbf.2014.0089
179. Scali, K., Roberts, J., McFarland, M., Marino, K., & Murray, L. (2018). IS MULTI-JOINT OR SINGLE JOINT STRENGTHENING MORE EFFECTIVE IN REDUCING PAIN AND IMPROVING FUNCTION IN WOMEN WITH PATELLOFEMORAL PAIN SYNDROME? A SYSTEMATIC REVIEW AND META-ANALYSIS. International journal of sports physical therapy, 13(3), 321-334. https://www.ncbi.nlm.nih.gov/pmc/articles/PMC6044587/pdf/ijspt-13-321.pdf
180. Selhorst, M., Fernandez-Fernandez, A., Schmitt, L., & Hoehn, J. (2020). Adolescent psychological beliefs, but not parent beliefs, associated with pain and function in adolescents with patellofemoral pain. Physical therapy in sport : official journal of the Association of Chartered Physiotherapists in Sports Medicine, 45, 155-160. https://doi.org/10.1016/j.ptsp.2020.07.003
181. Selhorst, M., Fernandez-Fernandez, A., Schmitt, L., & Hoehn, J. (2021). Effect of a Psychologically Informed Intervention to Treat Adolescents With Patellofemoral Pain: A Randomized Controlled Trial. Archives of physical medicine and rehabilitation, 102(7), 1267-1273. https://doi.org/10.1016/j.apmr.2021.03.016
182. Selhorst, M., Rice, W., Jackowski, M., Degenhart, T., & Coffman, S. (2018). A sequential cognitive and physical approach (SCOPA) for patellofemoral pain: a randomized controlled trial in adolescent patients. Clinical rehabilitation, 32(12), 1624-1635. https://doi.org/10.1177/0269215518787002
183. Selkowitz, D. M., Beneck, G. J., & Powers, C. M. (2023). Comparison of electromyographic activity of the gluteal muscles and tensor fascia lata in persons with patellofemoral pain: evaluation of selected, hip-targeted exercises using indwelling fine-wire electrodes. Physiotherapy Theory and Practice, 1-9. https://doi.org/10.1080/09593985.2023.2215389
184. Shadloo, N., Kamali, F., & Salehi Dehno, N. (2021). A comparison between whole-body vibration and conventional training on pain and performance in athletes with patellofemoral pain. Journal of bodywork and movement therapies, 27, 661-666. https://doi.org/10.1016/j.jbmt.2021.03.003
185. Shetty, K. M., Lawrence ; Hegde, Mahesh V. ; Shanmugam, Sukumar. (2016). Short - Term Effects of Eccentric Hip Abductors and Lateral Rotators Strengthening In Sedentary People with Patellofemoral Pain Syndrome on Pain and Function : A Randomized Control Trail. Journal of Health and Allied Sciences NU, 06(01), 68-73.
186. Silva, N. C., Silva, M. d. C., Tamburús, N. Y., Guimarães, M. G., Nascimento, M. B. d. O., & Felicio, L. R. (2023). Adding neuromuscular training to a strengthening program did not produce additional improvement in clinical or kinematic outcomes in women with patellofemoral pain: A blinded randomised controlled trial. Musculoskeletal Science & Practice, 63, 102720. https://doi.org/10.1016/j.msksp.2023.102720
187. Singh, S. T., Kunal ; Shweta, Shenoy ; Ravi, Saini. (2012). Concentric hip muscle function and quadriceps-hamstring ratio in athletes with and without patellofemoral pain syndrome. Ibnosina Journal of Medicine and Biomedical Sciences, 04(01), 20-27.
188. Souza, R. B., & Powers, C. M. (2009). Differences in hip kinematics, muscle strength, and muscle activation between subjects with and without patellofemoral pain. The Journal of orthopaedic and sports physical therapy, 39(1), 12-19. https://doi.org/10.2519/jospt.2009.2885
189. Steinberg, N., Tenenbaum, S., Waddington, G., Adams, R., Zakin, G., Zeev, A., & Siev-Ner, I. (2020). Unilateral and bilateral patellofemoral pain in young female dancers: Associated factors. Journal of sports sciences, 38(7), 719-730. https://doi.org/10.1080/02640414.2020.1727822
190. Steinberg, N., Tenenbaum, S., Waddington, G., Adams, R., Zakin, G., Zeev, A., & Siev-Ner, I. (2020). Isometric exercises and somatosensory training as intervention programmes for patellofemoral pain in young dancers. European Journal of Sport Science, 20(6), 845-857. https://doi.org/10.1080/17461391.2019.1675766

191. Theisen, B. J., Larson, P. D., & Chambers, C. C. (2022). Optimizing Rehabilitation and Return to Sport in Athletes With Anterior Knee Pain Using a Biomechanical Perspective. Arthroscopy, sports medicine, and rehabilitation, 4(1), e199-e207. https://doi.org/10.1016/j.asmr.2021.10.028
192. Thomson, C., Krouwel, O., Kuisma, R., & Hebron, C. (2016). The outcome of hip exercise in patellofemoral pain: A systematic review. Manual therapy, 26, 1-30. https://doi.org/10.1016/j.math.2016.06.003
193. Torlak, A. B.-B. F. (2015). Outcomes following Hip and Quadriceps Strengthening Exercises for Patellofemoral Syndrome. Sports, Vol 3, Iss 4, Pp 281-, 301(eng).
194. Toumi, H., Best, T. M., Pinti, A., Lavet, C., Benhamou, C. L., & Lespessailles, E. (2013). The role of muscle strength & activation patterns in patellofemoral pain. Clinical biomechanics (Bristol, Avon), 28(5), 544-548. https://doi.org/10.1016/j.clinbiomech.2013.04.005
195. Van Cant, J., Declève, P., Garnier, A., & Roy, J. S. (2021). Influence of symptom frequency and severity on hip abductor strength and endurance in individuals with patellofemoral pain. Physical therapy in sport : official journal of the Association of Chartered Physiotherapists in Sports Medicine, 49, 83-89. https://doi.org/10.1016/j.ptsp.2021.02.001
196. Van Cant, J., Pineux, C., Pitance, L., & Feipel, V. (2014). Hip muscle strength and endurance in females with patellofemoral pain: a systematic review with meta-analysis. International journal of sports physical therapy, 9(5), 564-582. https://www.ncbi.nlm.nih.gov/pmc/articles/PMC4196322/pdf/ijspt-10-564.pdf
197. Van Cant, J., Pitance, L., & Feipel, V. (2017). Hip abductor, trunk extensor and ankle plantar flexor endurance in females with and without patellofemoral pain. Journal of back and musculoskeletal rehabilitation, 30(2), 299-307. https://doi.org/10.3233/BMR-150505
198. Van Der Heijden, R. A., Lankhorst, N. E., Van Linschoten, R., Bierma-Zeinstra, S. M., & Van Middelkoop, M. (2016). Exercise for treating patellofemoral pain syndrome: an abridged version of Cochrane systematic review. Eur J Phys Rehabil Med, 52(1), 110-133.
199. van der Heijden, R. A., Lankhorst, N. E., van Linschoten, R., Bierma-Zeinstra, S. M. A., & van Middelkoop, M. (2015). Exercise for treating patellofemoral pain syndrome. The Cochrane database of systematic reviews, 1, CD010387. https://doi.org/10.1002/14651858.CD010387.pub2
200. Van Tiggelen, D., Cowan, S., Coorevits, P., Duvigneaud, N., & Witvrouw, E. (2009). Delayed vastus medialis obliquus to vastus lateralis onset timing contributes to the development of patellofemoral pain in previously healthy men: a prospective study. The American journal of sports medicine, 37(6), 1099-1105. https://doi.org/10.1177/0363546508331135
201. Vannatta, C. N., & Kernozek, T. W. (2015). Patellofemoral joint stress during running with alterations in foot strike pattern. Medicine and science in sports and exercise, 47(5), 1001-1008. https://doi.org/10.1249/MSS.0000000000000503
202. Waiteman, M. C., Chia, L., Ducatti, M. H. M., Bazett-Jones, D. M., Pappas, E., de Azevedo, F. M., & Briani, R. V. (2022). Trunk Biomechanics in Individuals with Knee Disorders: A Systematic Review with Evidence Gap Map and Meta-analysis. Sports medicine - open, 8(1), 145. https://doi.org/10.1186/s40798-022-00536-6
203. Wallis, J. A., Roddy, L., Bottrell, J., Parslow, S., & Taylor, N. F. (2021). A Systematic Review of Clinical Practice Guidelines for Physical Therapist Management of Patellofemoral Pain. Physical therapy, 101(3), pzab021. https://doi.org/10.1093/ptj/pzab021
204. Weiss, K., & Whatman, C. (2015). Biomechanics Associated with Patellofemoral Pain and ACL Injuries in Sports. Sports medicine (Auckland, N.Z.), 45(9), 1325-1337. https://doi.org/10.1007/s40279-015-0353-4
205. White, L. C., Dolphin, P., & Dixon, J. (2009). Hamstring length in patellofemoral pain syndrome. Physiotherapy, 95(1), 24-28. https://doi.org/10.1016/j.physio.2008.05.009
206. Willson, J. D., Ratcliff, O. M., Meardon, S. A., & Willy, R. W. (2015). Influence of step length and landing pattern on patellofemoral joint kinetics during running. Scandinavian journal of medicine & science in sports, 25(6), 736-743. https://doi.org/10.1111/sms.12383
207. Willson, J. D., Sharpee, R., Meardon, S. A., & Kernozek, T. W. (2014). Effects of step length on patellofemoral joint stress in female runners with and without patellofemoral pain. Clinical biomechanics (Bristol, Avon), 29(3), 243-247. https://doi.org/10.1016/j.clinbiomech.2013.12.016
208. Willy, R. W., Hoglund, L. T., Barton, C. J., Bolgla, L. A., Scalzitti, D. A., Logerstedt, D. S., Lynch, A. D., Snyder-Mackler, L., & McDonough, C. M. (2019). Patellofemoral Pain. The Journal of orthopaedic and sports physical therapy, 49(9), CPG1-CPG95. https://doi.org/10.2519/jospt.2019.0302

209. *Winters, M., Holden, S., Lura, C. B., Welton, N. J., Caldwell, D. M., Vicenzino, B. T., Weir, A., & Rathleff, M. S. (2020). Comparative effectiveness of treatments for patellofemoral pain: a living systematic review with network meta-analysis. British journal of sports medicine, 55(7), 369-377. https://doi.org/10.1136/bjsports-2020-102819*
210. *Xie, P., István, B., & Liang, M. (2022). The Relationship between Patellofemoral Pain Syndrome and Hip Biomechanics: A Systematic Review with Meta-Analysis. Healthcare (Basel, Switzerland), 11(1). https://doi.org/10.3390/healthcare11010099*
211. *Yalfani, A., & Ahmadi, M. (2023). Patients with Patellofemoral Pain Exhibiting Decrease Vertical Ground Reaction Force Compared to Healthy Individuals during Weight Bearing Tasks: A Systematic Reviews and Meta-Analysis. Iranian journal of public health, 52(2), 254-264. https://doi.org/10.18502/ijph.v52i2.11879*
212. *Yılmaz Yelvar, G. D., Baltacı, G., Bayrakcı Tunay, V., & Atay, A. Ö. (2015). The effect of postural stabilization exercises on pain and function in females with patellofemoral pain syndrome. Acta orthopaedica et traumatologica turcica, 49(2), 166-174. https://doi.org/10.3944/AOTT.2015.13.0118*
213. *Yosmaoglu, H. B., Kaya, D., Guney, H., Nyland, J., Baltaci, G., Yuksel, I., & Doral, M. N. (2013). Is there a relationship between tracking ability, joint position sense, and functional level in patellofemoral pain syndrome? Knee surgery, sports traumatology, arthroscopy : official journal of the ESSKA, 21(11), 2564-2571. https://doi.org/10.1007/s00167-013-2406-2*
214. *Young, J. L., Snodgrass, S. J., Cleland, J. A., & Rhon, D. I. (2021). Timing of physical therapy for individuals with patellofemoral pain and the influence on healthcare use, costs and recurrence rates: an observational study. BMC health services research, 21(1), 751. https://doi.org/10.1186/s12913-021-06768-8*
215. *Zuk, E. F., Kim, G., Rodriguez, J., Hallaway, B., Kuczo, A., Deluca, S., Allen, K., Glaviano, N. R., & DiStefano, L. J. (2021). The Utilization of Core Exercises in Patients With Patellofemoral Pain: A Critically Appraised Topic. Journal of sport rehabilitation, 30(7), 1094-1097. https://doi.org/10.1123/jsr.2020-0350*
