# Supplementary material for: Effectiveness of Self-Training With a Web-Based Digital Health Application Versus Physiotherapy in the Treatment of Disorders of the Patella: Randomized Controlled Trial
Source: J Med Internet Res. 2025 May 5;27:e66463. doi: 10.2196/66463 (PMC12089869; doi:10.2196/66463)
Supplement: Multimedia Appendix 3 [file jmir_v27i1e66463_app3.pdf]

List of pain medications

- Arcoxia
- Ibuprofen
- Naproxen
- Diclofenac (tablets and ointment)
- Metamizole
- Etoricoxib
- Novalgin
- Thomapyrin intensive (acetylsalicylic acid, paracetamol and caffeine)
- Tramadol 100 (one patient)
- Tilidine 50mg/4mg retard (one patient)
