# Supplementary material for: Effectiveness of Self-Training With a Web-Based Digital Health Application Versus Physiotherapy in the Treatment of Disorders of the Patella: Randomized Controlled Trial
Source: J Med Internet Res. 2025 May 5;27:e66463. doi: 10.2196/66463 (PMC12089869; doi:10.2196/66463)
Supplement: Multimedia Appendix 4 [file jmir_v27i1e66463_app4.pdf]

CONSORT-EHEALTH (V 1.6.1) - Submission/Publication Form

The CONSORT-EHEALTH checklist is intended for authors of randomized trials evaluating web-based and Internet-based applications/interventions, including mobile interventions, electronic games (incl multiplayer games), social media, certain telehealth applications, and other interactive and/or networked electronic applications. Some of the items (e.g. all subitems under item 5 - description of the intervention) may also be applicable for other study designs.

The goal of the CONSORT EHEALTH checklist and guideline is to be
a) a guide for reporting for authors of RCTs,
b) to form a basis for appraisal of an ehealth trial (in terms of validity)

CONSORT-EHEALTH items/subitems are MANDATORY reporting items for studies published in the Journal of Medical Internet Research and other journals / scientific societies endorsing the checklist.

Items numbered 1., 2., 3., 4a., 4b etc are original CONSORT or CONSORT-NPT (non-pharmacologic treatment) items.
Items with Roman numerals (i., ii, iii, iv etc.) are CONSORT-EHEALTH extensions/ clarifications.

As the CONSORT-EHEALTH checklist is still considered in a formative stage, we would ask that you also RATE ON A SCALE OF 1-5 how important/useful you feel each item is FOR THE PURPOSE OF THE CHECKLIST and reporting guideline (optional).

Mandatory reporting items are marked with a red *.
In the textboxes, either copy & paste the relevant sections from your manuscript into this form - please include any quotes from your manuscript in QUOTATION MARKS,
or answer directly by providing additional information not in the manuscript, or elaborating on why the item was not relevant for this study.

YOUR ANSWERS WILL BE PUBLISHED AS A SUPPLEMENTARY FILE TO YOUR PUBLICATION IN JMIR AND ARE CONSIDERED PART OF YOUR PUBLICATION (IF ACCEPTED).
Please fill in these questions diligently. Information will not be copyedited, so please use proper spelling and grammar, use correct capitalization, and avoid abbreviations.

DO NOT FORGET TO SAVE AS PDF _AND_ CLICK THE SUBMIT BUTTON SO YOUR ANSWERS ARE IN OUR DATABASE !!!

Citation Suggestion (if you append the pdf as Appendix we suggest to cite this paper in the caption):
[illegible]

Ihre Antwort ist zu lang. Kürzen Sie einige Sätze in Ihrer Antwort.

Mobile Health Interventions

J Med Internet Res 2011;13(4):e126
URL: http://www.jmir.org/2011/4/e126/
doi: 10.2196/jmir.1923
PMID: 22209829

In Google anmelden, um den Fortschritt zu speichern. Weitere Informationen

* Gibt eine erforderliche Frage an

Your name *

First Last

Tobias Mayer

Primary Affiliation (short), City, Country *

University of Toronto, Toronto, Canada

Chemnitz University of Technology, Germany

Your e-mail address *

abc@gmail.com

tobias.mayer@hsw.tu-chemnitz.de

Title of your manuscript *

Provide the (draft) title of your manuscript.

Effectiveness of self-training with a web-based digital health application vs. physiotherapy in the treatment of disorders of the patella: a randomized controlled trial

Ihre Antwort ist zu lang. Kürzen Sie einige Sätze in Ihrer Antwort.

Name of your App/Software/Intervention *

If there is a short and a long/alternate name, write the short name first and add the long name in brackets.

Mawendo

Evaluated Version (if any)

e.g. "V1", "Release 2017-03-01", "Version 2.0.27913"

Version 1.6

Language(s) *

What language is the intervention/app in? If multiple languages are available, separate by comma (e.g. "English, French")

German

URL of your Intervention Website or App

e.g. a direct link to the mobile app on app in appstore (itunes, Google Play), or URL of the website. If the intervention is a DVD or hardware, you can also link to an Amazon page.

https://www.mawendo.com/

URL of an image/screenshot (optional)

Meine Antwort

Ihre Antwort ist zu lang. Kürzen Sie einige Sätze in Ihrer Antwort.

Accessibility *

Can an enduser access the intervention presently?

- ○ access is free and open
- ○ access only for special usergroups, not open
- ◉ access is open to everyone, but requires payment/subscription/in-app purchases
- ○ app/intervention no longer accessible
- ○ Sonstiges:

Primary Medical Indication/Disease/Condition *

e.g. "Stress", "Diabetes", or define the target group in brackets after the condition, e.g. "Autism (Parents of children with)", "Alzheimers (Informal Caregivers of)"

ICD10-M22 (disorder of patella)

Primary Outcomes measured in trial *

comma-separated list of primary outcomes reported in the trial

knee function, knee pain

Secondary/other outcomes

Are there any other outcomes the intervention is expected to affect?

Meine Antwort

Ihre Antwort ist zu lang. Kürzen Sie einige Sätze in Ihrer Antwort.

Recommended "Dose" *

What do the instructions for users say on how often the app should be used?

- ( ) Approximately Daily
- ( ) Approximately Weekly
- ( ) Approximately Monthly
- ( ) Approximately Yearly
- ( ) "as needed"
- (x) Sonstiges: 2-3 times a week

Approx. Percentage of Users (starters) still using the app as recommended after 3 months *

- (x) unknown / not evaluated
- ( ) 0-10%
- ( ) 11-20%
- ( ) 21-30%
- ( ) 31-40%
- ( ) 41-50%
- ( ) 51-60%
- ( ) 61-70%
- ( ) 71%-80%
- ( ) 81-90%
- ( ) 91-100%
- ( ) Sonstiges:

Ihre Antwort ist zu lang. Kürzen Sie einige Sätze in Ihrer Antwort.

Overall, was the app/intervention effective? *

- (selected) yes: all primary outcomes were significantly better in intervention group vs control
- partly: SOME primary outcomes were significantly better in intervention group vs control
- no statistically significant difference between control and intervention
- potentially harmful: control was significantly better than intervention in one or more outcomes
- inconclusive: more research is needed
- Sonstiges:

Article Preparation Status/Stage *

At which stage in your article preparation are you currently (at the time you fill in this form)

- not submitted yet - in early draft status
- (selected) not submitted yet - in late draft status, just before submission
- submitted to a journal but not reviewed yet
- submitted to a journal and after receiving initial reviewer comments
- submitted to a journal and accepted, but not published yet
- published
- Sonstiges:

Ihre Antwort ist zu lang. Kürzen Sie einige Sätze in Ihrer Antwort.

Journal *

If you already know where you will submit this paper (or if it is already submitted), please provide the journal name (if it is not JMIR, provide the journal name under "other")

- ( ) not submitted yet / unclear where I will submit this
- (•) Journal of Medical Internet Research (JMIR)
- ( ) JMIR mHealth and UHealth
- ( ) JMIR Serious Games
- ( ) JMIR Mental Health
- ( ) JMIR Public Health
- ( ) JMIR Formative Research
- ( ) Other JMIR sister journal
- ( ) Sonstiges:

Is this a full powered effectiveness trial or a pilot/feasibility trial? *

- ( ) Pilot/feasibility
- (•) Fully powered

Manuscript tracking number *

If this is a JMIR submission, please provide the manuscript tracking number under "other" (The ms tracking number can be found in the submission acknowledgement email, or when you login as author in JMIR. If the paper is already published in JMIR, then the ms tracking number is the four-digit number at the end of the DOI, to be found at the bottom of each published article in JMIR)

- (•) no ms number (yet) / not (yet) submitted to / published in JMIR
- ( ) Sonstiges:

Ihre Antwort ist zu lang. Kürzen Sie einige Sätze in Ihrer Antwort.

TITLE AND ABSTRACT

1a) TITLE: Identification as a randomized trial in the title

1a) Does your paper address CONSORT item 1a? *

I.e does the title contain the phrase "Randomized Controlled Trial"? (if not, explain the reason under "other")

- (x) yes
- ( ) Sonstiges:

1a-i) Identify the mode of delivery in the title

Identify the mode of delivery. Preferably use “web-based” and/or “mobile” and/or “electronic game” in the title. Avoid ambiguous terms like “online”, “virtual”, “interactive”. Use “Internet-based” only if Intervention includes non-web-based Internet components (e.g. email), use “computer-based” or “electronic” only if offline products are used. Use “virtual” only in the context of “virtual reality” (3-D worlds). Use “online” only in the context of “online support groups”. Complement or substitute product names with broader terms for the class of products (such as “mobile” or “smart phone” instead of “iphone”), especially if the application runs on different platforms.

	1	2	3	4	5	
subitem not at all important	( )	( )	( )	( )	( )	essential

Does your paper address subitem 1a-i? *

Copy and paste relevant sections from manuscript title (include quotes in quotation marks "like this" to indicate direct quotes from your manuscript), or elaborate on this item by providing additional information not in the ms, or briefly explain why the item is not applicable/relevant for your study

Yes, "Effectiveness of self-training with a web-based digital health application vs. physiotherapy in the treatment of disorders of the patella: a randomized controlled trial"

Ihre Antwort ist zu lang. Kürzen Sie einige Sätze in Ihrer Antwort.

1a-ii) Non-web-based components or important co-interventions in title

Mention non-web-based components or important co-interventions in title, if any (e.g., “with telephone support”).

	1	2	3	4	5	
subitem not at all important	○	○	○	○	○	essential

Does your paper address subitem 1a-ii?

Copy and paste relevant sections from manuscript title (include quotes in quotation marks "like this" to indicate direct quotes from your manuscript), or elaborate on this item by providing additional information not in the ms, or briefly explain why the item is not applicable/relevant for your study

Not applicable to our study.

1a-iii) Primary condition or target group in the title

Mention primary condition or target group in the title, if any (e.g., “for children with Type I Diabetes”) Example: A Web-based and Mobile Intervention with Telephone Support for Children with Type I Diabetes: Randomized Controlled Trial

	1	2	3	4	5	
subitem not at all important	○	○	○	○	○	essential

Does your paper address subitem 1a-iii? *

Copy and paste relevant sections from manuscript title (include quotes in quotation marks "like this" to indicate direct quotes from your manuscript), or elaborate on this item by providing additional information not in the ms, or briefly explain why the item is not applicable/relevant for your study

Yes, "Effectiveness of self-training with a web-based digital health application vs. physiotherapy in the treatment of disorders of the patella: a randomized controlled trial"

Ihre Antwort ist zu lang. Kürzen Sie einige Sätze in Ihrer Antwort.

1b) ABSTRACT: Structured summary of trial design, methods, results, and conclusions
NPT extension: Description of experimental treatment, comparator, care providers, centers, and blinding status.

1b-i) Key features/functionalities/components of the intervention and comparator in the METHODS section of the ABSTRACT
Mention key features/functionalities/components of the intervention and comparator in the abstract. If possible, also mention theories and principles used for designing the site. Keep in mind the needs of systematic reviewers and indexers by including important synonyms. (Note: Only report in the abstract what the main paper is reporting. If this information is missing from the main body of text, consider adding it)

	1	2	3	4	5	
subitem not at all important	○	○	○	○	○	essential

Does your paper address subitem 1b-i? *
Copy and paste relevant sections from the manuscript abstract (include quotes in quotation marks "like this" to indicate direct quotes from your manuscript), or elaborate on this item by providing additional information not in the ms, or briefly explain why the item is not applicable/relevant for your study

Yes, criterion is fulfilled.

1b-ii) Level of human involvement in the METHODS section of the ABSTRACT
Clarify the level of human involvement in the abstract, e.g., use phrases like “fully automated” vs. “therapist/nurse/care provider/physician-assisted” (mention number and expertise of providers involved, if any). (Note: Only report in the abstract what the main paper is reporting. If this information is missing from the main body of text, consider adding it)

	1	2	3	4	5	
subitem not at all important	○	○	○	○	○	essential

Ihre Antwort ist zu lang. Kürzen Sie einige Sätze in Ihrer Antwort.

Does your paper address subitem 1b-ii?

Copy and paste relevant sections from the manuscript abstract (include quotes in quotation marks "like this" to indicate direct quotes from your manuscript), or elaborate on this item by providing additional information not in the ms, or briefly explain why the item is not applicable/relevant for your study

Yes, criterion is fulfilled in combination with section Objective.

1b-iii) Open vs. closed, web-based (self-assessment) vs. face-to-face assessments in the METHODS section of the ABSTRACT

Mention how participants were recruited (online vs. offline), e.g., from an open access website or from a clinic or a closed online user group (closed usergroup trial), and clarify if this was a purely web-based trial, or there were face-to-face components (as part of the intervention or for assessment). Clearly say if outcomes were self-assessed through questionnaires (as common in web-based trials). Note: In traditional offline trials, an open trial (open-label trial) is a type of clinical trial in which both the researchers and participants know which treatment is being administered. To avoid confusion, use “blinded” or “unblinded” to indicated the level of blinding instead of “open”, as “open” in web-based trials usually refers to “open access” (i.e. participants can self-enrol). (Note: Only report in the abstract what the main paper is reporting. If this information is missing from the main body of text, consider adding it)

	1	2	3	4	5	
subitem not at all important	○	○	○	○	○	essential

Does your paper address subitem 1b-iii?

Copy and paste relevant sections from the manuscript abstract (include quotes in quotation marks "like this" to indicate direct quotes from your manuscript), or elaborate on this item by providing additional information not in the ms, or briefly explain why the item is not applicable/relevant for your study

Yes, "A total of 259 patients with diagnosed disorders of the patella were included in the trial and randomly allocated to IG DHA (n=136) and CG SHI-PT (n=123)."

Ihre Antwort ist zu lang. Kürzen Sie einige Sätze in Ihrer Antwort.

1b-iv) RESULTS section in abstract must contain use data

Report number of participants enrolled/assessed in each group, the use/uptake of the intervention (e.g., attrition/adherence metrics, use over time, number of logins etc.), in addition to primary/secondary outcomes. (Note: Only report in the abstract what the main paper is reporting. If this information is missing from the main body of text, consider adding it)

	1	2	3	4	5	
subitem not at all important	○	○	○	○	○	essential

Does your paper address subitem 1b-iv?

Copy and paste relevant sections from the manuscript abstract (include quotes in quotation marks "like this" to indicate direct quotes from your manuscript), or elaborate on this item by providing additional information not in the ms, or briefly explain why the item is not applicable/relevant for your study

Yes, criterion is fulfilled.

1b-v) CONCLUSIONS/DISCUSSION in abstract for negative trials

Conclusions/Discussions in abstract for negative trials: Discuss the primary outcome - if the trial is negative (primary outcome not changed), and the intervention was not used, discuss whether negative results are attributable to lack of uptake and discuss reasons. (Note: Only report in the abstract what the main paper is reporting. If this information is missing from the main body of text, consider adding it)

	1	2	3	4	5	
subitem not at all important	○	○	○	○	○	essential

Ihre Antwort ist zu lang. Kürzen Sie einige Sätze in Ihrer Antwort.

Does your paper address subitem 1b-v?

Copy and paste relevant sections from the manuscript abstract (include quotes in quotation marks "like this" to indicate direct quotes from your manuscript), or elaborate on this item by providing additional information not in the ms, or briefly explain why the item is not applicable/relevant for your study

Not applicable to our study.

INTRODUCTION

2a) In INTRODUCTION: Scientific background and explanation of rationale

2a-i) Problem and the type of system/solution

Describe the problem and the type of system/solution that is object of the study: intended as stand-alone intervention vs. incorporated in broader health care program? Intended for a particular patient population? Goals of the intervention, e.g., being more cost-effective to other interventions, replace or complement other solutions? (Note: Details about the intervention are provided in “Methods” under 5)

	1	2	3	4	5	
subitem not at all important	○	○	○	○	○	essential

Ihre Antwort ist zu lang. Kürzen Sie einige Sätze in Ihrer Antwort.

Does your paper address subitem 2a-i? *

Copy and paste relevant sections from the manuscript (include quotes in quotation marks "like this" to indicate direct quotes from your manuscript), or elaborate on this item by providing additional information not in the ms, or briefly explain why the item is not applicable/relevant for your study

"The knee is the second most common site for musculoskeletal pain[1], with 11-17% of the cases affecting the anterior knee or the patellofemoral joint area. Muscular dysfunctions are considered an important biomechanical cause of such patella-related disorders[2–4]. Therefore, exercise therapy is widely regarded as an effective treatment strategy[4–8]. Conventional exercise therapy is typically administered on an outpatient basis under the guidance of a physiotherapist. In Germany, the benefits of having a trained physiotherapist, however, may be offset by drawbacks such as difficulties in scheduling appointments or a limited number of training sessions available due to regulatory limitations of German statutory health insurance. Therefore, home-based exercise interventions may present a valid alternative, as they have been reported to be at least as effective as traditional physiotherapy in reducing patellafemoral pain[9] and improving knee function[10]. "

2a-ii) Scientific background, rationale: What is known about the (type of) system

Scientific background, rationale: What is known about the (type of) system that is the object of the study (be sure to discuss the use of similar systems for other conditions/ diagnoses, if appropiate), motivation for the study, i.e. what are the reasons for and what is the context for this specific study, from which stakeholder viewpoint is the study performed, potential impact of findings [2]. Briefly justify the choice of the comparator.

	1	2	3	4	5	
subitem not at all important	○	○	○	○	○	essential

Ihre Antwort ist zu lang. Kürzen Sie einige Sätze in Ihrer Antwort.

Does your paper address subitem 2a-ii? *

Copy and paste relevant sections from the manuscript (include quotes in quotation marks "like this" to indicate direct quotes from your manuscript), or elaborate on this item by providing additional information not in the ms, or briefly explain why the item is not applicable/relevant for your study

"Kettunen et al.[11,12] found that an 8-week home exercise program was as effective as knee arthroscopy and an 8-week home exercise program together improving knee function and reducing pain. Similar findings have been reported for other knee conditions, such as meniscal tears[13–15], knee osteoarthritis[16,17], and other nonoperative knee conditions[18]. One way to deliver home-based exercise therapy is through digital health applications (DHA). These offer comprehensive possibilities to visualize exercises, educate about the condition in question, track training sessions and training progress, and boost individual training motivation. There is evidence that DHA may achieve better efficacy than conventional home training, for example, in treating degenerative meniscal tears[19], low back pain[20], or shoulder disorders[21]."

2b) In INTRODUCTION: Specific objectives or hypotheses

Does your paper address CONSORT subitem 2b? *

Copy and paste relevant sections from the manuscript (include quotes in quotation marks "like this" to indicate direct quotes from your manuscript), or elaborate on this item by providing additional information not in the ms, or briefly explain why the item is not applicable/relevant for your study

"In Germany, the Digital Healthcare Act allows for DHA to be covered by statutory health insurance once their efficacy is demonstrated. In recent years, a number of DHAs for exercise therapy[22–24] have been approved for coverage by German statutory health insurance. In this context, the present study aimed to evaluate the efficacy (improvement in knee function and/or pain) of a 12-week exercise intervention using a novel DHA (Mawendo, Biberach, Germany) compared to conventional physiotherapy covered by statutory health insurance in Germany (SHI-PT) in patients with disorders of the patella

METHODS

[illegible]

Ihre Antwort ist zu lang. Kürzen Sie einige Sätze in Ihrer Antwort.

Does your paper address CONSORT subitem 3a? *

Copy and paste relevant sections from the manuscript (include quotes in quotation marks "like this" to indicate direct quotes from your manuscript), or elaborate on this item by providing additional information not in the ms, or briefly explain why the item is not applicable/relevant for your study

"In this work, different strategies for treating diseases of the patella (ICD-10 M22) were compared between an active control group that received the current standard therapy in Germany (CG SHI-PT), and an intervention group using the DHA Mawendo (IG DHA). Participants and investigators were not blinded to the type of treatment or group allocation. All participants had to give written informed consent prior to enrollment in the study.", "Participants were allocated to the treatment arms using simple randomization within the following strata:

1) Recruiter
2) Pain medication intake of maximum WHO level 1 (yes/no)
3) Baseline VNRS pain level (3 - 4 / 5 - 7)"

3b) Important changes to methods after trial commencement (such as eligibility criteria), with reasons

Does your paper address CONSORT subitem 3b? *

Copy and paste relevant sections from the manuscript (include quotes in quotation marks "like this" to indicate direct quotes from your manuscript), or elaborate on this item by providing additional information not in the ms, or briefly explain why the item is not applicable/relevant for your study

Not applicable to our study.

Ihre Antwort ist zu lang. Kürzen Sie einige Sätze in Ihrer Antwort.

3b-i) Bug fixes, Downtimes, Content Changes

Bug fixes, Downtimes, Content Changes: ehealth systems are often dynamic systems. A description of changes to methods therefore also includes important changes made on the intervention or comparator during the trial (e.g., major bug fixes or changes in the functionality or content) (5-iii) and other "unexpected events" that may have influenced study design such as staff changes, system failures/downtimes, etc. [2].

	1	2	3	4	5	
subitem not at all important	○	○	○	○	○	essential

Does your paper address subitem 3b-i?

Copy and paste relevant sections from the manuscript (include quotes in quotation marks "like this" to indicate direct quotes from your manuscript), or elaborate on this item by providing additional information not in the ms, or briefly explain why the item is not applicable/relevant for your study

Not applicable to our study, no changes, no down times etc.

4a) Eligibility criteria for participants

Ihre Antwort ist zu lang. Kürzen Sie einige Sätze in Ihrer Antwort.

Does your paper address CONSORT subitem 4a? *

Copy and paste relevant sections from the manuscript (include quotes in quotation marks "like this" to indicate direct quotes from your manuscript), or elaborate on this item by providing additional information not in the ms, or briefly explain why the item is not applicable/relevant for your study

"The participants in this study were recruited by orthopedists (recruiters) during their regular consultation activities. Patients with confirmed disorders of the patella were eligible for participation. Children (age < 12 y) were not allowed to take part in the study. Adolescents (age < 18 y) required the consent of a legal representative. The pain intensity level was assessed by the recruiter prior to study inclusion using a Verbal Numerical Rating Scale[25] (VNRS, value range 0-10). Patients with very low (VNRS pain intensity ≤2) or very severe pain (VNRS pain intensity ≥ 8) were excluded from the study to control for disease severity. The following illnesses or physical conditions were defined as exclusion criteria:
(a) Knee surgery up to six months prior to the start of treatment
(b) Severe or acute diseases of the cardiovascular system (e.g., acute myocardial infarction, acute ischemic heart disease, high blood pressure with heart failure, or hypertensive crisis)
(c) Diseases of the lungs or respiratory tract (e.g., pneumonia, pulmonary embolism)
(d) Tumor diseases (e.g., malignant neoplasm of the bone and articular cartilage, malignant neoplasm of internal organs)
(e) Infection and fever (e.g., rheumatic fever, purulent arthritis, sepsis, bacterial infection)
(f) Injuries or diseases of the musculoskeletal system outside the indication ICD-10 M22
(g) Bleeding tendencies (history of increased bleeding or taking anticoagulant medication)
(h) Mental disorders (e.g., acute psychosis)

4a-i) Computer / Internet literacy

Computer / Internet literacy is often an implicit “de facto” eligibility criterion - this should be explicitly clarified.

	1	2	3	4	5	
subitem not at all important	○	○	○	○	○	essential

Ihre Antwort ist zu lang. Kürzen Sie einige Sätze in Ihrer Antwort.

Does your paper address subitem 4a-i?

Copy and paste relevant sections from the manuscript (include quotes in quotation marks "like this" to indicate direct quotes from your manuscript), or elaborate on this item by providing additional information not in the ms, or briefly explain why the item is not applicable/relevant for your study

There were no patients without internet access and literacy.

4a-ii) Open vs. closed, web-based vs. face-to-face assessments:

Open vs. closed, web-based vs. face-to-face assessments: Mention how participants were recruited (online vs. offline), e.g., from an open access website or from a clinic, and clarify if this was a purely web-based trial, or there were face-to-face components (as part of the intervention or for assessment), i.e., to what degree got the study team to know the participant. In online-only trials, clarify if participants were quasi-anonymous and whether having multiple identities was possible or whether technical or logistical measures (e.g., cookies, email confirmation, phone calls) were used to detect/prevent these.

	1	2	3	4	5	
subitem not at all important	○	○	○	○	○	essential

Does your paper address subitem 4a-ii? *

Copy and paste relevant sections from the manuscript (include quotes in quotation marks "like this" to indicate direct quotes from your manuscript), or elaborate on this item by providing additional information not in the ms, or briefly explain why the item is not applicable/relevant for your study

"The participants in this study were recruited by orthopedists (recruiters) during their regular consultation activities. Patients with confirmed disorders of the patella were eligible for participation. Children (age < 12 y) were not allowed to take part in the study. Adolescents (age < 18 y) required the consent of a legal representative. The pain intensity level was assessed by the recruiter prior to study inclusion using a Verbal Numerical Rating Scale[25] (VNRS, value range 0-10). Patients with very low (VNRS pain intensity ≤2) or very severe pain (VNRS pain intensity ≥ 8) were excluded from the study to control for

Ihre Antwort ist zu lang. Kürzen Sie einige Sätze in Ihrer Antwort.

4a-iii) Information giving during recruitment

Information given during recruitment. Specify how participants were briefed for recruitment and in the informed consent procedures (e.g., publish the informed consent documentation as appendix, see also item X26), as this information may have an effect on user self-selection, user expectation and may also bias results.

	1	2	3	4	5	
subitem not at all important	○	○	○	○	○	essential

Does your paper address subitem 4a-iii?

Copy and paste relevant sections from the manuscript (include quotes in quotation marks "like this" to indicate direct quotes from your manuscript), or elaborate on this item by providing additional information not in the ms, or briefly explain why the item is not applicable/relevant for your study

"All participants had to give written informed consent prior to enrollment in the study."

4b) Settings and locations where the data were collected

Ihre Antwort ist zu lang. Kürzen Sie einige Sätze in Ihrer Antwort.

Does your paper address CONSORT subitem 4b? *

Copy and paste relevant sections from the manuscript (include quotes in quotation marks "like this" to indicate direct quotes from your manuscript), or elaborate on this item by providing additional information not in the ms, or briefly explain why the item is not applicable/relevant for your study

We don't exactly know where the patients completed the surveys, because: "The endpoints were assessed using an electronic questionnaire accessible using a web browser and an internet connection. The questionnaire was hosted and operated independently from any IT infrastructure associated with the DHA.

Participants were asked to complete a survey twice during the course of the study:

PRE: Before the first training session with DHA or the first physiotherapy treatment.

POST: After completing the 12-week treatment period.

Participants received the survey URL by email at the specified times and were reminded a maximum of three times by email to complete the pending questionnaires."

4b-i) Report if outcomes were (self-)assessed through online questionnaires

Clearly report if outcomes were (self-)assessed through online questionnaires (as common in web-based trials) or otherwise.

	1	2	3	4	5	
subitem not at all important	○	○	○	○	○	essential

Ihre Antwort ist zu lang. Kürzen Sie einige Sätze in Ihrer Antwort.

Does your paper address subitem 4b-i? *

Copy and paste relevant sections from the manuscript (include quotes in quotation marks "like this" to indicate direct quotes from your manuscript), or elaborate on this item by providing additional information not in the ms, or briefly explain why the item is not applicable/relevant for your study

"The endpoints were assessed using an electronic questionnaire accessible using a web browser and an internet connection. The questionnaire was hosted and operated independently from any IT infrastructure associated with the DHA.

Participants were asked to complete a survey twice during the course of the study:

PRE: Before the first training session with DHA or the first physiotherapy treatment.

POST: After completing the 12-week treatment period.

Participants received the survey URL by email at the specified times and were reminded a maximum of three times by email to complete the pending questionnaires."

4b-ii) Report how institutional affiliations are displayed

Report how institutional affiliations are displayed to potential participants [on ehealth media], as affiliations with prestigious hospitals or universities may affect volunteer rates, use, and reactions with regards to an intervention.(Not a required item – describe only if this may bias results)

	1	2	3	4	5	
subitem not at all important	○	○	○	○	○	essential

Does your paper address subitem 4b-ii?

Copy and paste relevant sections from the manuscript (include quotes in quotation marks "like this" to indicate direct quotes from your manuscript), or elaborate on this item by providing additional information not in the ms, or briefly explain why the item is not applicable/relevant for your study

Not applicable to our study.

Ihre Antwort ist zu lang. Kürzen Sie einige Sätze in Ihrer Antwort.

5) The interventions for each group with sufficient details to allow replication, including how and when they were actually administered

5-i) Mention names, credential, affiliations of the developers, sponsors, and owners

Mention names, credential, affiliations of the developers, sponsors, and owners [6] (if authors/evaluators are owners or developer of the software, this needs to be declared in a “Conflict of interest” section or mentioned elsewhere in the manuscript).

	1	2	3	4	5	
subitem not at all important	○	○	○	○	○	essential

Does your paper address subitem 5-i?

Copy and paste relevant sections from the manuscript (include quotes in quotation marks "like this" to indicate direct quotes from your manuscript), or elaborate on this item by providing additional information not in the ms, or briefly explain why the item is not applicable/relevant for your study

Yey criterion is fullfilled.

5-ii) Describe the history/development process

Describe the history/development process of the application and previous formative evaluations (e.g., focus groups, usability testing), as these will have an impact on adoption/use rates and help with interpreting results.

	1	2	3	4	5	
subitem not at all important	○	○	○	○	○	essential

Ihre Antwort ist zu lang. Kürzen Sie einige Sätze in Ihrer Antwort.

Does your paper address subitem 5-ii?

Copy and paste relevant sections from the manuscript (include quotes in quotation marks "like this" to indicate direct quotes from your manuscript), or elaborate on this item by providing additional information not in the ms, or briefly explain why the item is not applicable/relevant for your study

Not applicable to our study.

5-iii) Revisions and updating

Revisions and updating. Clearly mention the date and/or version number of the application/intervention (and comparator, if applicable) evaluated, or describe whether the intervention underwent major changes during the evaluation process, or whether the development and/or content was "frozen" during the trial. Describe dynamic components such as news feeds or changing content which may have an impact on the replicability of the intervention (for unexpected events see item 3b).

	1	2	3	4	5	
subitem not at all important	○	○	○	○	○	essential

Does your paper address subitem 5-iii?

Copy and paste relevant sections from the manuscript (include quotes in quotation marks "like this" to indicate direct quotes from your manuscript), or elaborate on this item by providing additional information not in the ms, or briefly explain why the item is not applicable/relevant for your study

No revions or updates, Mawendo v1.6 was used.

5-iv) Quality assurance methods

Provide information on quality assurance methods to ensure accuracy and quality of information provided [1], if applicable.

	1	2	3	4	5
subitem not at all	○	○	○	○	○

Ihre Antwort ist zu lang. Kürzen Sie einige Sätze in Ihrer Antwort.

Does your paper address subitem 5-iv?

Copy and paste relevant sections from the manuscript (include quotes in quotation marks "like this" to indicate direct quotes from your manuscript), or elaborate on this item by providing additional information not in the ms, or briefly explain why the item is not applicable/relevant for your study

All digital health applications in Germany, so called DiGA have to be certified medical devices. Mawendo is a certified medical device (risk class 1). "IG DHA was treated using DHA Mawendo v1.6 (certified medical device, risk class 1) and used the DHA at any suitable location."

5-v) Ensure replicability by publishing the source code, and/or providing screenshots/screen-capture video, and/or providing flowcharts of the algorithms used

Ensure replicability by publishing the source code, and/or providing screenshots/screen-capture video, and/or providing flowcharts of the algorithms used. Replicability (i.e., other researchers should in principle be able to replicate the study) is a hallmark of scientific reporting.

	1	2	3	4	5	
subitem not at all important	○	○	○	○	○	essential

Does your paper address subitem 5-v?

Copy and paste relevant sections from the manuscript (include quotes in quotation marks "like this" to indicate direct quotes from your manuscript), or elaborate on this item by providing additional information not in the ms, or briefly explain why the item is not applicable/relevant for your study

Yes criterion is fullfilled. "Data and R Scripts are available on request due to privacy or other restrictions."

Ihre Antwort ist zu lang. Kürzen Sie einige Sätze in Ihrer Antwort.

5-vi) Digital preservation

Digital preservation: Provide the URL of the application, but as the intervention is likely to change or disappear over the course of the years; also make sure the intervention is archived (Internet Archive, webcitation.org, and/or publishing the source code or screenshots/videos alongside the article). As pages behind login screens cannot be archived, consider creating demo pages which are accessible without login.

	1	2	3	4	5	
subitem not at all important	○	○	○	○	○	essential

Does your paper address subitem 5-vi?

Copy and paste relevant sections from the manuscript (include quotes in quotation marks "like this" to indicate direct quotes from your manuscript), or elaborate on this item by providing additional information not in the ms, or briefly explain why the item is not applicable/relevant for your study

The intervention has been approved by the German Federal Institute for Drugs and Medical Devices for the treatment of disorders of the patella in persons of all sexes aged 12 and over and is permantly listed in the DiGA-Verzeichnis (https://diga.bfarm.de/de)

5-vii) Access

Access: Describe how participants accessed the application, in what setting/context, if they had to pay (or were paid) or not, whether they had to be a member of specific group. If known, describe how participants obtained "access to the platform and Internet" [1]. To ensure access for editors/reviewers/readers, consider to provide a "backdoor" login account or demo mode for reviewers/readers to explore the application (also important for archiving purposes, see vi).

	1	2	3	4	5	
subitem not at all important	○	○	○	○	○	essential

Ihre Antwort ist zu lang. Kürzen Sie einige Sätze in Ihrer Antwort.

Does your paper address subitem 5-vii? *

Copy and paste relevant sections from the manuscript (include quotes in quotation marks "like this" to indicate direct quotes from your manuscript), or elaborate on this item by providing additional information not in the ms, or briefly explain why the item is not applicable/relevant for your study

"IG DHA was treated using DHA Mawendo v1.6 (certified medical device, risk class 1) and used the DHA at any suitable location.", "The DHA used in this study is a browser-based web application that provides digital exercise videos with self-explanatory exercise instructions, health information, and documentation options for self-administered home training. Training sessions using the DHA contain sets of exercises lasting between 20-40 minutes, corresponding to the duration of a physiotherapy session. The user interface of the app includes the following sections: overview (1), education and information (2), therapy plan (3), and exercises (4).
In the overview section (1), users can record their training frequency and pain levels. Entries are used to feed a progress chart, which is used to track changes over time. The education and information section (2) contains information and educational material regarding the indication being treated. The therapy plan section (3) includes a therapy timeline, which is divided into three successive phases: basic, advanced, and stabilization. To ensure training progress and to avoid excessive demands, the three phases are designed to provide increasing exercise intensity and difficulty. In each phase, 8-16 exercises are provided (mobilization, coordination, strengthening, stretching, and massage), which should be performed 2 to 3 times a week during the treatment period of 12 weeks. The exercise section (4) provides instructional videos for the exercises to be performed by participants in their current therapy phase. Exercises are selected and compiled based on the guidelines of scientific medical societies and other scientific studies[26–45].
For IG DHA, the treating orthopedist selected and individually configured a training program in the DHA for the 12-week treatment period. Participants could initiate their

Ihre Antwort ist zu lang. Kürzen Sie einige Sätze in Ihrer Antwort.

5-viii) Mode of delivery, features/functionalities/components of the intervention and comparator, and the theoretical framework

Describe mode of delivery, features/functionalities/components of the intervention and comparator, and the theoretical framework [6] used to design them (instructional strategy [1], behaviour change techniques, persuasive features, etc., see e.g., [7, 8] for terminology). This includes an in-depth description of the content (including where it is coming from and who developed it) [1]," whether [and how] it is tailored to individual circumstances and allows users to track their progress and receive feedback" [6]. This also includes a description of communication delivery channels and – if computer-mediated communication is a component – whether communication was synchronous or asynchronous [6]. It also includes information on presentation strategies [1], including page design principles, average amount of text on pages, presence of hyperlinks to other resources, etc. [1].

	1	2	3	4	5	
subitem not at all important	○	○	○	○	○	essential

Ihre Antwort ist zu lang. Kürzen Sie einige Sätze in Ihrer Antwort.

Does your paper address subitem 5-viii? *

Copy and paste relevant sections from the manuscript (include quotes in quotation marks "like this" to indicate direct quotes from your manuscript), or elaborate on this item by providing additional information not in the ms, or briefly explain why the item is not applicable/relevant for your study

"The DHA used in this study is a browser-based web application that provides digital exercise videos with self-explanatory exercise instructions, health information, and documentation options for self-administered home training. Training sessions using the DHA contain sets of exercises lasting between 20-40 minutes, corresponding to the duration of a physiotherapy session. The user interface of the app includes the following sections: overview (1), education and information (2), therapy plan (3), and exercises (4). In the overview section (1), users can record their training frequency and pain levels. Entries are used to feed a progress chart, which is used to track changes over time. The education and information section (2) contains information and educational material regarding the indication being treated. The therapy plan section (3) includes a therapy timeline, which is divided into three successive phases: basic, advanced, and stabilization. To ensure training progress and to avoid excessive demands, the three phases are designed to provide increasing exercise intensity and difficulty. In each phase, 8-16 exercises are provided (mobilization, coordination, strengthening, stretching, and massage), which should be performed 2 to 3 times a week during the treatment period of 12 weeks. The exercise section (4) provides instructional videos for the exercises to be performed by participants in their current therapy phase. Exercises are selected and compiled based on the guidelines of scientific medical societies and other scientific studies[26–45].
For IG DHA, the treating orthopedist selected and individually configured a training program in the DHA for the 12-week treatment period. Participants could initiate their treatment immediately after recruitment."

5-ix) Describe use parameters

Describe use parameters (e.g., intended “doses” and optimal timing for use). Clarify what instructions or recommendations were given to the user, e.g., regarding timing, frequency, heaviness of use, if any, or was the intervention used ad libitum.

	1	2	3	4	5	
subitem not at all important	○	○	○	○	○	essential

Ihre Antwort ist zu lang. Kürzen Sie einige Sätze in Ihrer Antwort.

Does your paper address subitem 5-ix?

Copy and paste relevant sections from the manuscript (include quotes in quotation marks "like this" to indicate direct quotes from your manuscript), or elaborate on this item by providing additional information not in the ms, or briefly explain why the item is not applicable/relevant for your study

"The DHA used in this study is a browser-based web application that provides digital exercise videos with self-explanatory exercise instructions, health information, and documentation options for self-administered home training. Training sessions using the DHA contain sets of exercises lasting between 20-40 minutes, corresponding to the duration of a physiotherapy session. The user interface of the app includes the following sections: overview (1), education and information (2), therapy plan (3), and exercises (4). In the overview section (1), users can record their training frequency and pain levels. Entries are used to feed a progress chart, which is used to track changes over time. The education and information section (2) contains information and educational material regarding the indication being treated. The therapy plan section (3) includes a therapy timeline, which is divided into three successive phases: basic, advanced, and stabilization. To ensure training progress and to avoid excessive demands, the three phases are designed to provide increasing exercise intensity and difficulty. In each phase, 8-16 exercises are provided (mobilization, coordination, strengthening, stretching, and massage), which should be performed 2 to 3 times a week during the treatment period of 12 weeks. The exercise section (4) provides instructional videos for the exercises to be performed by participants in their current therapy phase. Exercises are selected and compiled based on the guidelines of scientific medical societies and other scientific studies[26–45].
For IG DHA, the treating orthopedist selected and individually configured a training program in the DHA for the 12-week treatment period. Participants could initiate their

5-x) Clarify the level of human involvement

Clarify the level of human involvement (care providers or health professionals, also technical assistance) in the e-intervention or as co-intervention (detail number and expertise of professionals involved, if any, as well as “type of assistance offered, the timing and frequency of the support, how it is initiated, and the medium by which the assistance is delivered”. It may be necessary to distinguish between the level of human involvement required for the trial, and the level of human involvement required for a routine application outside of a RCT setting (discuss under item 21 – generalizability).

	1	2	3	4	5	
subitem not at all important	○	○	○	○	○	essential

Ihre Antwort ist zu lang. Kürzen Sie einige Sätze in Ihrer Antwort.

Does your paper address subitem 5-x?

Copy and paste relevant sections from the manuscript (include quotes in quotation marks "like this" to indicate direct quotes from your manuscript), or elaborate on this item by providing additional information not in the ms, or briefly explain why the item is not applicable/relevant for your study

"Treatment of CG SHI-PT
Physiotherapeutic treatment in Germany includes measures that can be prescribed according to the German Heilmittel-Richtlinie §92 Abs. 1 S2 No. 6 SGB V (German Therapeutic Products Guidelines, §92 Para. 1 Sentence 2 No. 6 Social Code V):

- preparation of an individual treatment plan
- assistance provided by the physiotherapist
- execution of physiotherapeutic measures on/with the patient
- standard treatment duration
- necessary rest following treatment
- progress documentation and, if necessary, progress report to the prescribing physician
- additional work and administrative tasks

Ideally, the first physiotherapy treatment session should include a medical history, pain localization and, if necessary, mobility tests. The physiotherapist then develops a therapy plan that is implemented in the subsequent sessions. This may include active and passive mobilization and strengthening exercises, or manual therapy. The number of sessions and prescriptions within the study period was determined by the treating orthopedist according to the current statutory health regulations in Germany. These permit 6 to12 sessions during a 12-week treatment period, with up to 18 sessions allowed in severe cases.
Treatment of IG DHA
The DHA used in this study is a browser-based web application that provides digital exercise videos with self-explanatory exercise instructions, health information, and documentation options for self-administered home training. Training sessions using the DHA contain sets of exercises lasting between 20-40 minutes, corresponding to the duration of a physiotherapy session. The user interface of the app includes the following sections: overview (1), education and information (2), therapy plan (3), and exercises (4). In the overview section (1), users can record their training frequency and pain levels. Entries are used to feed a progress chart, which is used to track changes over time. The education and information section (2) contains information and educational material regarding the indication being treated. The therapy plan section (3) includes a therapy timeline, which is divided into three successive phases: basic, advanced, and stabilization. To ensure training progress and to avoid excessive demands, the three phases are designed to provide increasing exercise intensity and difficulty. In each phase, 8-16 exercises are provided (mobilization, coordination, strengthening, stretching, and massage), which should be performed 2 to 3 times a week during the treatment period of 12 weeks. The exercise section (4) provides instructional videos for the exercises to be performed by participants in their current therapy phase. Exercises are selected and compiled based on the guidelines of scientific medical societies and other scientific studies[26–45].
For IG DHA, the treating orthopedist selected and individually configured a training

Ihre Antwort ist zu lang. Kürzen Sie einige Sätze in Ihrer Antwort.

5-xi) Report any prompts/reminders used

Report any prompts/reminders used: Clarify if there were prompts (letters, emails, phone calls, SMS) to use the application, what triggered them, frequency etc. It may be necessary to distinguish between the level of prompts/reminders required for the trial, and the level of prompts/reminders for a routine application outside of a RCT setting (discuss under item 21 – generalizability).

	1	2	3	4	5	
subitem not at all important	○	○	○	○	○	essential

Does your paper address subitem 5-xi? *

Copy and paste relevant sections from the manuscript (include quotes in quotation marks "like this" to indicate direct quotes from your manuscript), or elaborate on this item by providing additional information not in the ms, or briefly explain why the item is not applicable/relevant for your study

"Participants received the survey URL by email at the specified times and were reminded a maximum of three times by email to complete the pending questionnaires."

5-xii) Describe any co-interventions (incl. training/support)

Describe any co-interventions (incl. training/support): Clearly state any interventions that are provided in addition to the targeted eHealth intervention, as ehealth intervention may not be designed as stand-alone intervention. This includes training sessions and support [1]. It may be necessary to distinguish between the level of training required for the trial, and the level of training for a routine application outside of a RCT setting (discuss under item 21 – generalizability.

	1	2	3	4	5	
subitem not at all important	○	○	○	○	○	essential

Ihre Antwort ist zu lang. Kürzen Sie einige Sätze in Ihrer Antwort.

Does your paper address subitem 5-xii? *

Copy and paste relevant sections from the manuscript (include quotes in quotation marks "like this" to indicate direct quotes from your manuscript), or elaborate on this item by providing additional information not in the ms, or briefly explain why the item is not applicable/relevant for your study

Not applicable to our study.

6a) Completely defined pre-specified primary and secondary outcome measures, including how and when they were assessed

Ihre Antwort ist zu lang. Kürzen Sie einige Sätze in Ihrer Antwort.

Does your paper address CONSORT subitem 6a? *

Copy and paste relevant sections from the manuscript (include quotes in quotation marks "like this" to indicate direct quotes from your manuscript), or elaborate on this item by providing additional information not in the ms, or briefly explain why the item is not applicable/relevant for your study

"Two primary endpoints were examined in this study:

1) Improvement in ‘knee function’
2) Reduction of ‘knee pain’

Both endpoints describe different aspects of positive treatment effects and were therefore considered to be equally valid to assess medical benefit. The study was designed to evaluate therapeutic superiority of the DHA over SHI-PT in at least one of the two endpoints to comply with regulations given by the Federal Institute for Drugs and Medical Devices in Germany (Bundesinstitut für Arzneimittel und Medizinprodukte, BfArM).
The endpoints were assessed using an electronic questionnaire accessible using a web browser and an internet connection. The questionnaire was hosted and operated independently from any IT infrastructure associated with the DHA.

Participants were asked to complete a survey twice during the course of the study:

PRE: Before the first training session with DHA or the first physiotherapy treatment.

POST: After completing the 12-week treatment period.

Participants received the survey URL by email at the specified times and were reminded a maximum of three times by email to complete the pending questionnaires.
The endpoint ‘knee function’ was quantified using the German version of the Patient Reported Outcome (PRO) KOOS (Knee Injury and Osteoarthritis Outcome Score)[46]. KOOS is a validated questionnaire that measures aspects of health and functional status in diseases and injuries of the knee joint using 5 scales (‘Pain’, ‘Symptoms’, ‘Activities of daily Living’, ‘Functionality in sports and leisure’, ‘Quality of life-related to the affected knee’). The ‘Activities of daily Living’ (KOOSADL) subscale was used to represent ‘knee function’. The KOOSADL score is calculated from the 17 ADL items, with each item scored from best (0 p) to worst (4 p). A KOOSADL score of zero p represents extreme knee problems and 100 p represents no knee problems.

〖KOOS〗_ADL=100-(1/n ∑_(i=1)^(n=17)▒〖〖ItemScore〗_i*100〗)/4

The endpoint ‘knee pain’ was assessed using a separate pain score based on a visual analogue scale (VAS, range 0-100 p, where 100 p represents worst pain imaginable). Participants were asked to quantify the intensity of their pain over the past week[47]."

Ihre Antwort ist zu lang. Kürzen Sie einige Sätze in Ihrer Antwort.

6a-i) Online questionnaires: describe if they were validated for online use and apply CHERRIES items to describe how the questionnaires were designed/ deployed

If outcomes were obtained through online questionnaires, describe if they were validated for online use and apply CHERRIES items to describe how the questionnaires were designed/deployed [9].

	1	2	3	4	5	
subitem not at all important	○	○	○	○	○	essential

Does your paper address subitem 6a-i?

Copy and paste relevant sections from manuscript text

The online KOOS questionaire was an exact replication of the German paper version. All the CHERRIES items should addressed in the methods section.

6a-ii) Describe whether and how “use” (including intensity of use/dosage) was defined/measured/monitored

Describe whether and how “use” (including intensity of use/dosage) was defined/ measured/monitored (logins, logfile analysis, etc.). Use/adoption metrics are important process outcomes that should be reported in any ehealth trial.

	1	2	3	4	5	
subitem not at all important	○	○	○	○	○	essential

Does your paper address subitem 6a-ii?

Copy and paste relevant sections from manuscript text

Usage was not specifically monitored, but collected by self-reporting in the post survey.

Ihre Antwort ist zu lang. Kürzen Sie einige Sätze in Ihrer Antwort.

6a-iii) Describe whether, how, and when qualitative feedback from participants was obtained

Describe whether, how, and when qualitative feedback from participants was obtained (e.g., through emails, feedback forms, interviews, focus groups).

	1	2	3	4	5	
subitem not at all important	○	○	○	○	○	essential

Does your paper address subitem 6a-iii?

Copy and paste relevant sections from manuscript text

No qualitative feedback was collected in our study.

6b) Any changes to trial outcomes after the trial commenced, with reasons

Does your paper address CONSORT subitem 6b? *

Copy and paste relevant sections from the manuscript (include quotes in quotation marks "like this" to indicate direct quotes from your manuscript), or elaborate on this item by providing additional information not in the ms, or briefly explain why the item is not applicable/relevant for your study

No changes to the prespecification.

7a) How sample size was determined

NPT: When applicable, details of whether and how the clustering by care provides or centers was addressed

Ihre Antwort ist zu lang. Kürzen Sie einige Sätze in Ihrer Antwort.

7a-i) Describe whether and how expected attrition was taken into account when calculating the sample size

Describe whether and how expected attrition was taken into account when calculating the sample size.

	1	2	3	4	5	
subitem not at all important	○	○	○	○	○	essential

Does your paper address subitem 7a-i?

Copy and paste relevant sections from manuscript title (include quotes in quotation marks "like this" to indicate direct quotes from your manuscript), or elaborate on this item by providing additional information not in the ms, or briefly explain why the item is not applicable/relevant for your study

"Sample size
Due to multiple testing (one hypothesis for each of the two primary endpoints), the significance level was adjusted using the Bonferroni method and set at α = 0.0125 (one-sided testing). The minimal clinically important difference (MCID) was used as the effect size to be detected, and a power of 1 - β = 0.9 was specified to calculate the required sample size for a superiority scenario. MCIDs for both outcomes were determined from several studies (KOOSADL : 10 p (sd = 15 p) [48–50], VAS: 10 p (sd=22 p) [47,51,52]). This resulted in sample sizes of n=56 (KOOSADL) and n=121 (VAS). The more conservative (larger) sample size of n=121 was used to generate sufficient statistical power for both endpoints. Sample sizes were determined using the SampleSize4ClinicalTrials R package[53]."

7b) When applicable, explanation of any interim analyses and stopping guidelines

Does your paper address CONSORT subitem 7b? *

Copy and paste relevant sections from the manuscript (include quotes in quotation marks "like this" to indicate direct quotes from your manuscript), or elaborate on this item by providing additional information not in the ms, or briefly explain why the item is not applicable/relevant for your study

Not applicable to our study.

Ihre Antwort ist zu lang. Kürzen Sie einige Sätze in Ihrer Antwort.

8a) Method used to generate the random allocation sequence
NPT: When applicable, how care providers were allocated to each trial group

Does your paper address CONSORT subitem 8a? *

Copy and paste relevant sections from the manuscript (include quotes in quotation marks "like this" to indicate direct quotes from your manuscript), or elaborate on this item by providing additional information not in the ms, or briefly explain why the item is not applicable/relevant for your study

"Participants were allocated to the treatment arms using simple randomization within the following strata:
1) Recruiter
2) Pain medication intake of maximum WHO level 1 (yes/no)
3) Baseline VNRS pain level (3 - 4 / 5 - 7)"

8b) Type of randomisation; details of any restriction (such as blocking and block size)

Does your paper address CONSORT subitem 8b? *

Copy and paste relevant sections from the manuscript (include quotes in quotation marks "like this" to indicate direct quotes from your manuscript), or elaborate on this item by providing additional information not in the ms, or briefly explain why the item is not applicable/relevant for your study

"Randomization
Participants were allocated to the treatment arms using simple randomization within the following strata:
1) Recruiter
2) Pain medication intake of maximum WHO level 1 (yes/no)
3) Baseline VNRS pain level (3 - 4 / 5 - 7)"

9) Mechanism used to implement the random allocation sequence (such as sequentially numbered containers), describing any steps taken to conceal the sequence until interventions were assigned

Ihre Antwort ist zu lang. Kürzen Sie einige Sätze in Ihrer Antwort.

Does your paper address CONSORT subitem 9? *

Copy and paste relevant sections from the manuscript (include quotes in quotation marks "like this" to indicate direct quotes from your manuscript), or elaborate on this item by providing additional information not in the ms, or briefly explain why the item is not applicable/relevant for your study

"Current literature provided no evidence for potential bias between treatment arms for treatment history, stage of disease, age, or sex in previous studies, and thus did not include them as strata for randomization. A browser-based web application (Rek-app, Mawendo, Biberach, Germany) created specifically for recruiting purposes was provided to the recruiters. A set of 4 randomization lists (2 levels for pain medication × 2 levels for baseline VNRS pain) with 100 patients each was stored for each recruiter in the Rek-app. The statistical software R (version 3.6.2) was used to create these randomization lists [26]. The recruiters used the Rek-app to check exclusion criteria, include participants in the study, and randomly allocate participants to treatment arms according to the stored randomization lists (allocation sequence was blinded to the recruiters). Rek-app provided recruiters and participants with a participant-specific ID, through which the participants gained access to the web-based survey platform. A total of seven recruitment centers were involved with this study. Figure 1 provides a summary of the recruitment process."

10) Who generated the random allocation sequence, who enrolled participants, and who assigned participants to interventions

Ihre Antwort ist zu lang. Kürzen Sie einige Sätze in Ihrer Antwort.

Does your paper address CONSORT subitem 10? *

Copy and paste relevant sections from the manuscript (include quotes in quotation marks "like this" to indicate direct quotes from your manuscript), or elaborate on this item by providing additional information not in the ms, or briefly explain why the item is not applicable/relevant for your study

Ihre Antwort ist zu lang. Kürzen Sie einige Sätze in Ihrer Antwort.

"Study population
The participants in this study were recruited by orthopedists (recruiters) during their regular consultation activities. Patients with confirmed disorders of the patella were eligible for participation. Children (age < 12 y) were not allowed to take part in the study. Adolescents (age < 18 y) required the consent of a legal representative. The pain intensity level was assessed by the recruiter prior to study inclusion using a Verbal Numerical Rating Scale[25] (VNRS, value range 0-10). Patients with very low (VNRS pain intensity ≤2) or very severe pain (VNRS pain intensity ≥ 8) were excluded from the study to control for disease severity. The following illnesses or physical conditions were defined as exclusion criteria:
(a) Knee surgery up to six months prior to the start of treatment
(b) Severe or acute diseases of the cardiovascular system (e.g., acute myocardial infarction, acute ischemic heart disease, high blood pressure with heart failure, or hypertensive crisis)
(c) Diseases of the lungs or respiratory tract (e.g., pneumonia, pulmonary embolism)
(d) Tumor diseases (e.g., malignant neoplasm of the bone and articular cartilage, malignant neoplasm of internal organs)
(e) Infection and fever (e.g., rheumatic fever, purulent arthritis, sepsis, bacterial infection)
(f) Injuries or diseases of the musculoskeletal system outside the indication ICD-10 M22
(g) Bleeding tendencies (history of increased bleeding or taking anticoagulant medication)
(h) Mental disorders (e.g., acute psychosis)
(i) Severe visual impairment
(j) Pregnancy
The study data were analyzed according to the intention-to-treat principle. Therefore, the occurrence of an exclusion criterion during the study did not lead to the exclusion of participants from further data analysis.
Randomization
Participants were allocated to the treatment arms using simple randomization within the following strata:
1) Recruiter
2) Pain medication intake of maximum WHO level 1 (yes/no)
3) Baseline VNRS pain level (3 - 4 / 5 - 7)
Current literature provided no evidence for potential bias between treatment arms for treatment history, stage of disease, age, or sex in previous studies, and thus did not include them as strata for randomization. A browser-based web application (Rek-app, Mawendo, Biberach, Germany) created specifically for recruiting purposes was provided to the recruiters. A set of 4 randomization lists (2 levels for pain medication × 2 levels for baseline VNRS pain) with 100 patients each was stored for each recruiter in the Rek-app. The statistical software R (version 3.6.2) was used to create these randomization lists [26]. The recruiters used the Rek-app to check exclusion criteria, include participants in the study, and randomly allocate participants to treatment arms according to the stored randomization lists (allocation sequence was blinded to the recruiters). Rek-app provided recruiters and participants with a participant-specific ID, through which the participants

Ihre Antwort ist zu lang. Kürzen Sie einige Sätze in Ihrer Antwort.

11a) If done, who was blinded after assignment to interventions (for example, participants, care providers, those assessing outcomes) and how
NPT: Whether or not administering co-interventions were blinded to group assignment

11a-i) Specify who was blinded, and who wasn't
Specify who was blinded, and who wasn't. Usually, in web-based trials it is not possible to blind the participants [1, 3] (this should be clearly acknowledged), but it may be possible to blind outcome assessors, those doing data analysis or those administering co-interventions (if any).

	1	2	3	4	5	
subitem not at all important	○	○	○	○	○	essential

Does your paper address subitem 11a-i? *
Copy and paste relevant sections from the manuscript (include quotes in quotation marks "like this" to indicate direct quotes from your manuscript), or elaborate on this item by providing additional information not in the ms, or briefly explain why the item is not applicable/relevant for your study

Not applicable to our study

11a-ii) Discuss e.g., whether participants knew which intervention was the "intervention of interest" and which one was the "comparator"
Informed consent procedures (4a-ii) can create biases and certain expectations - discuss e.g., whether participants knew which intervention was the "intervention of interest" and which one was the "comparator".

	1	2	3	4	5	
subitem not at all important	○	○	○	○	○	essential

Ihre Antwort ist zu lang. Kürzen Sie einige Sätze in Ihrer Antwort.

Does your paper address subitem 11a-ii?

Copy and paste relevant sections from the manuscript (include quotes in quotation marks "like this" to indicate direct quotes from your manuscript), or elaborate on this item by providing additional information not in the ms, or briefly explain why the item is not applicable/relevant for your study

"Treatment strategies were not blinded to patients or recruiters, since the therapy strategies cannot be effectively hidden from either. Blinding created through a placebo app was not implemented, since BfArM guidelines for the evaluation of DHAs require evidence of superiority over usual care (SHI-PT in Germany), which does not include any additional DHA. Data collection was performed using web-based electronic questionnaires with only participants themselves being involved in generating the data. We can therefore rule out virtually any bias on our results created by the lack of blinding in our study."

11b) If relevant, description of the similarity of interventions

(this item is usually not relevant for ehealth trials as it refers to similarity of a placebo or sham intervention to a active medication/intervention)

Does your paper address CONSORT subitem 11b? *

Copy and paste relevant sections from the manuscript (include quotes in quotation marks "like this" to indicate direct quotes from your manuscript), or elaborate on this item by providing additional information not in the ms, or briefly explain why the item is not applicable/relevant for your study

Not applicable to our study.

12a) Statistical methods used to compare groups for primary and secondary outcomes

NPT: When applicable, details of whether and how the clustering by care providers or centers was addressed

Ihre Antwort ist zu lang. Kürzen Sie einige Sätze in Ihrer Antwort.

Does your paper address CONSORT subitem 12a? *

Copy and paste relevant sections from the manuscript (include quotes in quotation marks "like this" to indicate direct quotes from your manuscript), or elaborate on this item by providing additional information not in the ms, or briefly explain why the item is not applicable/relevant for your study

"Statistical analysis
Hypothesis tests were performed on null hypotheses of DHA not providing superior medical benefit in either one of the endpoints compared to SHI-PT.
A linear model (ANCOVA) was used to determine the effectiveness of DHA for both endpoints. The outcome score in the respective endpoint ('knee function', 'knee pain') at POST was used as the dependent variable, the treatment group as the predictor variable, and the baseline score at PRE as a covariate. The strata 'recruitment center', 'pain medication intake', and 'VNRS pain level' were used as control variables. Type II sum of squares were used due to simple randomization and unbalanced group sizes."

12a-i) Imputation techniques to deal with attrition / missing values

Imputation techniques to deal with attrition / missing values: Not all participants will use the intervention/comparator as intended and attrition is typically high in ehealth trials. Specify how participants who did not use the application or dropped out from the trial were treated in the statistical analysis (a complete case analysis is strongly discouraged, and simple imputation techniques such as LOCF may also be problematic [4]).

	1	2	3	4	5	
subitem not at all important	○	○	○	○	○	essential

Ihre Antwort ist zu lang. Kürzen Sie einige Sätze in Ihrer Antwort.

Does your paper address subitem 12a-i? *

Copy and paste relevant sections from the manuscript (include quotes in quotation marks "like this" to indicate direct quotes from your manuscript), or elaborate on this item by providing additional information not in the ms, or briefly explain why the item is not applicable/relevant for your study

"Missing values in either PRE or POST were imputed using the jump to reference method (J2R, R package RefBasedMI [55,56]). For KOOSADL, the imputations were executed at item level and the total KOOSADL score was calculated for each of 50 imputations (reference group: SHI PT, covariates: 'recruitment center', 'pain medication intake', and 'VNRS pain level'). Implausible values less than 0 resulting from the J2R imputation were replaced by the value 0 and floating point numbers were rounded to integers. Variables that were not required for the inferential statistical analysis of the two primary clinical endpoints, such as adherence data or information on additional therapies, were not

12b) Methods for additional analyses, such as subgroup analyses and adjusted analyses

Does your paper address CONSORT subitem 12b? *

Copy and paste relevant sections from the manuscript (include quotes in quotation marks "like this" to indicate direct quotes from your manuscript), or elaborate on this item by providing additional information not in the ms, or briefly explain why the item is not applicable/relevant for your study

Not applicable to our study.

X26) REB/IRB Approval and Ethical Considerations [recommended as subheading under "Methods"] (not a CONSORT item)

X26-i) Comment on ethics committee approval

	1	2	3	4	5	
subitem not at all	○	○	○	○	○	essential

Ihre Antwort ist zu lang. Kürzen Sie einige Sätze in Ihrer Antwort.

Does your paper address subitem X26-i?

Copy and paste relevant sections from the manuscript (include quotes in quotation marks "like this" to indicate direct quotes from your manuscript), or elaborate on this item by providing additional information not in the ms, or briefly explain why the item is not applicable/relevant for your study

"The prespecified study protocol was approved and registered by the Institutional Ethics Committee of the Faculty of Behavioural and Social Sciences of the Chemnitz University of Technology (registration number: V-439-17-CM-MAWENDO-II-18042021) in agreement with current data protection regulations."

x26-ii) Outline informed consent procedures

Outline informed consent procedures e.g., if consent was obtained offline or online (how? Checkbox, etc.?), and what information was provided (see 4a-ii). See [6] for some items to be included in informed consent documents.

	1	2	3	4	5	
subitem not at all important	○	○	○	○	○	essential

Does your paper address subitem X26-ii?

Copy and paste relevant sections from the manuscript (include quotes in quotation marks "like this" to indicate direct quotes from your manuscript), or elaborate on this item by providing additional information not in the ms, or briefly explain why the item is not applicable/relevant for your study

"All participants had to give written informed consent prior to enrollment in the study."
Fig.1 details the recruiting process.

Ihre Antwort ist zu lang. Kürzen Sie einige Sätze in Ihrer Antwort.

X26-iii) Safety and security procedures

Safety and security procedures, incl. privacy considerations, and any steps taken to reduce the likelihood or detection of harm (e.g., education and training, availability of a hotline)

	1	2	3	4	5	
subitem not at all important	○	○	○	○	○	essential

Does your paper address subitem X26-iii?

Copy and paste relevant sections from the manuscript (include quotes in quotation marks "like this" to indicate direct quotes from your manuscript), or elaborate on this item by providing additional information not in the ms, or briefly explain why the item is not applicable/relevant for your study

Not applicable to our study.

RESULTS

13a) For each group, the numbers of participants who were randomly assigned, received intended treatment, and were analysed for the primary outcome

NPT: The number of care providers or centers performing the intervention in each group and the number of patients treated by each care provider in each center

Ihre Antwort ist zu lang. Kürzen Sie einige Sätze in Ihrer Antwort.

Does your paper address CONSORT subitem 13a? *

Copy and paste relevant sections from the manuscript (include quotes in quotation marks "like this" to indicate direct quotes from your manuscript), or elaborate on this item by providing additional information not in the ms, or briefly explain why the item is not applicable/relevant for your study

"259 participants were recruited between November 10, 2021 and January 21, 2023, and randomly assigned to either IG DHA or CG SHI-PT. IG DHA (n=136) received treatment using the DHA while CG SHI-PT (n=123) received SHI-PT. Six participants (IG DHA: n = 4, CG SHI-PT: n = 2) provided no survey data despite several e-mail inquiries. These participants were assumed to be missing completely at random and excluded from further analysis. Subsequently, data from 253 participants (IG DHA: n= 132; CG SHI-PT: n = 121) were analyzed. Another 6 of the remaining 253 participants provided incomplete datasets (IG DHA: n = 1; CG SHI-PT: n = 5; assumption missing at random): one participant did not provide PRE data, and 5 participants were lost to follow-up (POST data missing). These missing data were imputed for the inferential statistical analysis (jump to reference, J2R) [59]. Figure 2 summarizes the above information in a flowchart according to

13b) For each group, losses and exclusions after randomisation, together with reasons

Does your paper address CONSORT subitem 13b? (NOTE: Preferably, this is shown in a CONSORT flow diagram) *

Copy and paste relevant sections from the manuscript (include quotes in quotation marks "like this" to indicate direct quotes from your manuscript), or elaborate on this item by providing additional information not in the ms, or briefly explain why the item is not applicable/relevant for your study

"259 participants were recruited between November 10, 2021 and January 21, 2023, and randomly assigned to either IG DHA or CG SHI-PT. IG DHA (n=136) received treatment using the DHA while CG SHI-PT (n=123) received SHI-PT. Six participants (IG DHA: n = 4, CG SHI-PT: n = 2) provided no survey data despite several e-mail inquiries. These participants were assumed to be missing completely at random and excluded from further analysis. Subsequently, data from 253 participants (IG DHA: n= 132; CG SHI-PT: n = 121) were analyzed. Another 6 of the remaining 253 participants provided incomplete datasets (IG DHA: n = 1; CG SHI-PT: n = 5; assumption missing at random): one participant did not provide PRE data, and 5 participants were lost to follow-up (POST data missing). These missing data were imputed for the inferential statistical analysis (jump to reference, J2R) [59]. Figure 2 summarizes the above information in a flowchart according to

Ihre Antwort ist zu lang. Kürzen Sie einige Sätze in Ihrer Antwort.

13b-i) Attrition diagram

Strongly recommended: An attrition diagram (e.g., proportion of participants still logging in or using the intervention/comparator in each group plotted over time, similar to a survival curve) or other figures or tables demonstrating usage/dose/engagement.

	1	2	3	4	5	
subitem not at all important	○	○	○	○	○	essential

Does your paper address subitem 13b-i?

Copy and paste relevant sections from the manuscript or cite the figure number if applicable (include quotes in quotation marks "like this" to indicate direct quotes from your manuscript), or elaborate on this item by providing additional information not in the ms, or briefly explain why the item is not applicable/relevant for your study

Yes criterion is fullfilled.

14a) Dates defining the periods of recruitment and follow-up

Does your paper address CONSORT subitem 14a? *

Copy and paste relevant sections from the manuscript (include quotes in quotation marks "like this" to indicate direct quotes from your manuscript), or elaborate on this item by providing additional information not in the ms, or briefly explain why the item is not applicable/relevant for your study

"November 10, 2021 and January 21, 2023"

Ihre Antwort ist zu lang. Kürzen Sie einige Sätze in Ihrer Antwort.

14a-i) Indicate if critical “secular events” fell into the study period

Indicate if critical “secular events” fell into the study period, e.g., significant changes in Internet resources available or “changes in computer hardware or Internet delivery resources”

	1	2	3	4	5	
subitem not at all important	○	○	○	○	○	essential

Does your paper address subitem 14a-i?

Copy and paste relevant sections from the manuscript (include quotes in quotation marks "like this" to indicate direct quotes from your manuscript), or elaborate on this item by providing additional information not in the ms, or briefly explain why the item is not applicable/relevant for your study

Not applicable to our study.

14b) Why the trial ended or was stopped (early)

Does your paper address CONSORT subitem 14b? *

Copy and paste relevant sections from the manuscript (include quotes in quotation marks "like this" to indicate direct quotes from your manuscript), or elaborate on this item by providing additional information not in the ms, or briefly explain why the item is not applicable/relevant for your study

The trial was stopped after reaching the prespecified sample size.

15) A table showing baseline demographic and clinical characteristics for each group

NPT: When applicable, a description of care providers (case volume, qualification, expertise, etc.) and centers (volume) in each group

Ihre Antwort ist zu lang. Kürzen Sie einige Sätze in Ihrer Antwort.

Does your paper address CONSORT subitem 15? *

Copy and paste relevant sections from the manuscript (include quotes in quotation marks "like this" to indicate direct quotes from your manuscript), or elaborate on this item by providing additional information not in the ms, or briefly explain why the item is not applicable/relevant for your study

Yes criterion ist fullfilled.

15-i) Report demographics associated with digital divide issues

In ehealth trials it is particularly important to report demographics associated with digital divide issues, such as age, education, gender, social-economic status, computer/Internet/ehealth literacy of the participants, if known.

	1	2	3	4	5	
subitem not at all important	○	○	○	○	○	essential

Does your paper address subitem 15-i? *

Copy and paste relevant sections from the manuscript (include quotes in quotation marks "like this" to indicate direct quotes from your manuscript), or elaborate on this item by providing additional information not in the ms, or briefly explain why the item is not applicable/relevant for your study

Yes criterion ist fullfilled, as far as information was available.

16) For each group, number of participants (denominator) included in each analysis and whether the analysis was by original assigned groups

Ihre Antwort ist zu lang. Kürzen Sie einige Sätze in Ihrer Antwort.

16-i) Report multiple “denominators” and provide definitions

Report multiple “denominators” and provide definitions: Report N’s (and effect sizes) “across a range of study participation [and use] thresholds” [1], e.g., N exposed, N consented, N used more than x times, N used more than y weeks, N participants “used” the intervention/comparator at specific pre-defined time points of interest (in absolute and relative numbers per group). Always clearly define “use” of the intervention.

	1	2	3	4	5	
subitem not at all important	○	○	○	○	○	essential

Does your paper address subitem 16-i? *

Copy and paste relevant sections from the manuscript (include quotes in quotation marks "like this" to indicate direct quotes from your manuscript), or elaborate on this item by providing additional information not in the ms, or briefly explain why the item is not applicable/relevant for your study

"259 participants were recruited between November 10, 2021 and January 21, 2023, and randomly assigned to either IG DHA or CG SHI-PT. IG DHA (n=136) received treatment using the DHA while CG SHI-PT (n=123) received SHI-PT. Six participants (IG DHA: n = 4, CG SHI-PT: n = 2) provided no survey data despite several e-mail inquiries. These participants were assumed to be missing completely at random and excluded from further analysis. Subsequently, data from 253 participants (IG DHA: n= 132; CG SHI-PT: n = 121) were analyzed. Another 6 of the remaining 253 participants provided incomplete datasets (IG DHA: n = 1; CG SHI-PT: n = 5; assumption missing at random): one participant did not provide PRE data, and 5 participants were lost to follow-up (POST data missing). These missing data were imputed for the inferential statistical analysis (jump to reference, J2R) [59]. Figure 2 summarizes the above information in a flowchart according to

16-ii) Primary analysis should be intent-to-treat

Primary analysis should be intent-to-treat, secondary analyses could include comparing only “users”, with the appropriate caveats that this is no longer a randomized sample (see 18-i).

	1	2	3	4	5	
subitem not at all important	○	○	○	○	○	essential

Ihre Antwort ist zu lang. Kürzen Sie einige Sätze in Ihrer Antwort.

Does your paper address subitem 16-ii?

Copy and paste relevant sections from the manuscript (include quotes in quotation marks "like this" to indicate direct quotes from your manuscript), or elaborate on this item by providing additional information not in the ms, or briefly explain why the item is not applicable/relevant for your study

Yes criterion is fullfilled.

17a) For each primary and secondary outcome, results for each group, and the estimated effect size and its precision (such as 95% confidence interval)

Ihre Antwort ist zu lang. Kürzen Sie einige Sätze in Ihrer Antwort.

Does your paper address CONSORT subitem 17a? *

Copy and paste relevant sections from the manuscript (include quotes in quotation marks "like this" to indicate direct quotes from your manuscript), or elaborate on this item by providing additional information not in the ms, or briefly explain why the item is not applicable/relevant for your study

"Primary endpoint ‘knee function’ (KOOSADL score)
In IG DHA, the pooled mean KOOSADL score improved from 66.2 p (95 % CI [64.3 p; 68.1 p]) to 81.9 p (95 % CI [80.3 p; 83.5 p]) from PRE to POST. This corresponds to an improvement in ‘knee function’ of 15.7 p (95 % CI [13.7 p; 17.6 p]).
In CG SHI-PT, ‘knee function’ (pooled mean KOOSADL score) improved by 3.5 p (95 % CI [1.5 p; 5.5 p]) from 70.4 p (95 % CI [68.3 p; 72.5 p]) PRE to 73.9 p (95 % CI [71.5 p; 76.3 p]) POST.
Training with DHA resulted in a 4.5 times greater improvement in ‘knee function’ compared to SHI-PT. Figure 3 illustrates this result using the imputed data set with the largest effect for CG SHI-PT. Table 3 summarizes the results of the primary endpoint ‘knee function’.
The difference in KOOSADL score between IG DHA and CG SHI-PT (factor 'Intervention') was estimated at -10.1 p [Inf; -8.0 p] (adjusted one-sided 95 % CI, see Table 5) by the pooled ANCOVA and was statistically significant (P < .001, see Table 4)."

"Primary endpoint ‘knee pain’ (VAS pain score)
In IG DHA, the pooled mean VAS pain score decreased from 48.2 p (95 % CI [46.1 p; 50.3 p]) to 25.6 p (95 % CI [23.5 p; 27.8 p]) from PRE to POST. This corresponds to an improvement in ‘knee pain’ of -22.5 p (95 % CI [-25.2 p; -19.9 p]).
In CG SHI-PT, pooled mean VAS pain score improved by -6.5 p (95 % CI [-8.7 p; -4.4 p]) from PRE 44.7 p (95 % CI [42.4 p; 47.1 p]) to POST 38.2 p (95 % CI [35.3 p; 41.0 p]).
Thus, training with the DHA resulted in a 3.5 times greater reduction in ‘knee pain’ compared to SHI-PT. Figure 4 illustrates this result using the imputed data set with the greatest effect for CG SHI-PT. Table 6 summarizes the VAS pain score results of the primary endpoint ‘knee pain’. In the pooled ANCOVA for VAS pain score, the group difference between IG DHA and CG SHI-PT (factor 'intervention') was estimated at 14.3 p [11.7 p; Inf] (adjusted one-sided 95 % CI, see Table 8), and was also statistically significant

Ihre Antwort ist zu lang. Kürzen Sie einige Sätze in Ihrer Antwort.

17a-i) Presentation of process outcomes such as metrics of use and intensity of use

In addition to primary/secondary (clinical) outcomes, the presentation of process outcomes such as metrics of use and intensity of use (dose, exposure) and their operational definitions is critical. This does not only refer to metrics of attrition (13-b) (often a binary variable), but also to more continuous exposure metrics such as "average session length". These must be accompanied by a technical description how a metric like a "session" is defined (e.g., timeout after idle time) [1] (report under item 6a).

	1	2	3	4	5	
subitem not at all important	○	○	○	○	○	essential

Does your paper address subitem 17a-i?

Copy and paste relevant sections from the manuscript (include quotes in quotation marks "like this" to indicate direct quotes from your manuscript), or elaborate on this item by providing additional information not in the ms, or briefly explain why the item is not applicable/relevant for your study

Not applicable to our study.

17b) For binary outcomes, presentation of both absolute and relative effect sizes is recommended

Does your paper address CONSORT subitem 17b? *

Copy and paste relevant sections from the manuscript (include quotes in quotation marks "like this" to indicate direct quotes from your manuscript), or elaborate on this item by providing additional information not in the ms, or briefly explain why the item is not applicable/relevant for your study

Not applicable to our study.

18) Results of any other analyses performed, including subgroup analyses and

Ihre Antwort ist zu lang. Kürzen Sie einige Sätze in Ihrer Antwort.

Does your paper address CONSORT subitem 18? *

Copy and paste relevant sections from the manuscript (include quotes in quotation marks "like this" to indicate direct quotes from your manuscript), or elaborate on this item by providing additional information not in the ms, or briefly explain why the item is not applicable/relevant for your study

Not applicable to our study.

18-i) Subgroup analysis of comparing only users

A subgroup analysis of comparing only users is not uncommon in ehealth trials, but if done, it must be stressed that this is a self-selected sample and no longer an unbiased sample from a randomized trial (see 16-iii).

	1	2	3	4	5	
subitem not at all important	○	○	○	○	○	essential

Does your paper address subitem 18-i?

Copy and paste relevant sections from the manuscript (include quotes in quotation marks "like this" to indicate direct quotes from your manuscript), or elaborate on this item by providing additional information not in the ms, or briefly explain why the item is not applicable/relevant for your study

Not applicable to our study.

19) All important harms or unintended effects in each group

(for specific guidance see CONSORT for harms)

Ihre Antwort ist zu lang. Kürzen Sie einige Sätze in Ihrer Antwort.

Does your paper address CONSORT subitem 19? *

Copy and paste relevant sections from the manuscript (include quotes in quotation marks "like this" to indicate direct quotes from your manuscript), or elaborate on this item by providing additional information not in the ms, or briefly explain why the item is not applicable/relevant for your study

Not applicable to our study.

19-i) Include privacy breaches, technical problems

Include privacy breaches, technical problems. This does not only include physical “harm” to participants, but also incidents such as perceived or real privacy breaches [1], technical problems, and other unexpected/unintended incidents. “Unintended effects” also includes unintended positive effects [2].

	1	2	3	4	5	
subitem not at all important	○	○	○	○	○	essential

Does your paper address subitem 19-i?

Copy and paste relevant sections from the manuscript (include quotes in quotation marks "like this" to indicate direct quotes from your manuscript), or elaborate on this item by providing additional information not in the ms, or briefly explain why the item is not applicable/relevant for your study

"Two participants (IG DHA: n=1, CG SHI-PT: n=1) reported a change in therapy during the course of the study due to time restrictions (IG DHA) and supplementary medication (CG SHI-PT). Three participants in CG SHI-PT reported the occurrence of one or more exclusion criteria during the treatment phase. Two participants reported an infection during treatment (answer option ‘Infections and fever (e.g., rheumatic fever, purulent arthritis, sepsis, bacterial infections)’) and one participant reported an unspecified mental disorder (answer option ‘Mental disorders (e.g., acute psychosis)’)."

Ihre Antwort ist zu lang. Kürzen Sie einige Sätze in Ihrer Antwort.

19-ii) Include qualitative feedback from participants or observations from staff/researchers

Include qualitative feedback from participants or observations from staff/researchers, if available, on strengths and shortcomings of the application, especially if they point to unintended/unexpected effects or uses. This includes (if available) reasons for why people did or did not use the application as intended by the developers.

	1	2	3	4	5	
subitem not at all important	○	○	○	○	○	essential

Does your paper address subitem 19-ii?

Copy and paste relevant sections from the manuscript (include quotes in quotation marks "like this" to indicate direct quotes from your manuscript), or elaborate on this item by providing additional information not in the ms, or briefly explain why the item is not applicable/relevant for your study

Not applicable to our study.

DISCUSSION

22) Interpretation consistent with results, balancing benefits and harms, and considering other relevant evidence

NPT: In addition, take into account the choice of the comparator, lack of or partial blinding, and unequal expertise of care providers or centers in each group

22-i) Restate study questions and summarize the answers suggested by the data, starting with primary outcomes and process outcomes (use)

Restate study questions and summarize the answers suggested by the data, starting with primary outcomes and process outcomes (use).

1	2	3	4	5

Ihre Antwort ist zu lang. Kürzen Sie einige Sätze in Ihrer Antwort.

Does your paper address subitem 22-i? *

Copy and paste relevant sections from the manuscript (include quotes in quotation marks "like this" to indicate direct quotes from your manuscript), or elaborate on this item by providing additional information not in the ms, or briefly explain why the item is not applicable/relevant for your study

"Primary outcomes
This study provides evidence of the clinical superiority of DHA over SHI-PT for both primary endpoints, 'knee function' (KOOSADL score) and 'knee pain' (VAS pain score).
Knee function
The improvement achieved by IG DHA in our study (15.7 p KOOSADL score) not only meets the criterion of clinical relevance, but also that of 'substantial clinical benefit', suggested by several previous studies[49,51,61]. The improvement through SHI-PT was 4.5 times lower than in IG DHA and did not reach clinical relevance in our study.
To the best of our knowledge, the present study is the first RCT comparing a DHA to SHI-PT for ICD-10 M22. Only few other studies provide comparable results. Comparing a 12-week "Digital Care Program for Chronic Knee Pain on Pain, Mobility, and Surgery Risk" intervention to an education-only control group, Mecklenburg et al.[50] observed a reduction of 17 % in KOOS-PS score in IG DHA compared to only 3.7 % in CG SHI-PT. The difference between the "Digital Care Program" group and the control group was -7.2 p (95 % CI [-11.5 p; -3 p], P = .001). In a previous uncontrolled study on the efficacy of the Mawendo DHA, Kölle et al.[10] reported an improvement in the Kujala[62] score of 14 % over a treatment period of 12 weeks. In the present study, 'knee function' improved by 23.7 % in IG DHA and by 4.8 % in CG SHI-PT. Although different PROs/scores and control conditions were used, effects are largely in line across these studies and allow for similar conclusions that DHAs are more effective for improving 'knee function' than conventional physiotherapeutic interventions.
Knee pain
The pain reduction of 22.5 p (VAS pain score) in IG DHA classifies as clinically relevant, and as substantially clinically beneficial[52,53]. We observed no clinically relevant effects for pain reduction through SHI-PT in our study.
No similar studies were found that quantify treatment effects of DHA for knee pain using a VAS pain score. However, our VAS pain score results are consistent with similar RCTs investigating the performance of DHAs for other orthopedic diseases. Comparing DHA to physiotherapy interventions, Toelle et al.[22] found a 47 % reduction in back pain using the Kaia DHA vs. 37 % for physiotherapy, and Weise et al.[23] reported a 53.1 % reduction in back pain using the Vivira DHA vs. 14.6 % for physiotherapy. In the present study, the Mawendo DHA achieved a 47% reduction in 'knee pain' compared to 14 % through SHI-PT. Using the same DHA and treatment period as in the current study, Kölle et al.[10] reported a 64% reduction in knee pain on a knee pain VAS. In summary, our results for the endpoint

Ihre Antwort ist zu lang. Kürzen Sie einige Sätze in Ihrer Antwort.

22-ii) Highlight unanswered new questions, suggest future research

Highlight unanswered new questions, suggest future research.

	1	2	3	4	5	
subitem not at all important	○	○	○	○	○	essential

Does your paper address subitem 22-ii?

Copy and paste relevant sections from the manuscript (include quotes in quotation marks "like this" to indicate direct quotes from your manuscript), or elaborate on this item by providing additional information not in the ms, or briefly explain why the item is not applicable/relevant for your study

"Comparison of treatment arms
We found large between-group differences for both endpoints in our study. One reason may be different treatment strategies used by different physiotherapists. Furthermore, an earlier start of treatment is associated with faster healing [63–66], and problems scheduling timely appointments may in turn limit the efficacy of SHI-PT. In comparison, self-managed training using a DHA can begin immediately after being prescribed by a doctor and eliminates the need for travel, further anamnesis, and potential waiting for appointments. DHAs also allow for significantly more treatment sessions during a 12-week intervention period, since the German regulations usually limit SHI-PT to 6 to 12 physiotherapy sessions (maximum 18 sessions) during the same period. We hypothesize the greater training frequency to be one of the causal reasons for the significantly greater treatment success of DHA compared to SHI-PT. The actual start of treatment after diagnosis and the exact treatment frequency were not recorded in the present study. Further research should therefore address the relationship between onset of treatment, treatment frequency, and therapy success using DHAs."

"Adherence & change of therapy
In both treatment arms, self-reporting revealed a comparably high level of adherence: 89.4% of IG DHA patients and 85.1% of CG SHI-PT patients attended ‘all’ or ‘almost all’ therapy sessions. Thus, treatment frequency, and not adherence, may provide a better explanation for the between-group differences found in our study. In addition, similar treatment changes occurred in both treatment arms (1 participant in each group; no information for 2 participants in DHA, and 7 participants in SHI-PT). We found a slightly larger POST dropout rate in SHI-PT (4 in SHI-PT vs. 1 in DHA). The reasons for this are speculative and may include the above-mentioned scheduling and travel required for SHI-

Ihre Antwort ist zu lang. Kürzen Sie einige Sätze in Ihrer Antwort.

20-i) Typical limitations in ehealth trials

Typical limitations in ehealth trials: Participants in ehealth trials are rarely blinded. Ehealth trials often look at a multiplicity of outcomes, increasing risk for a Type I error. Discuss biases due to non-use of the intervention/usability issues, biases through informed consent procedures, unexpected events.

	1	2	3	4	5	
subitem not at all important	○	○	○	○	○	essential

Does your paper address subitem 20-i? *

Copy and paste relevant sections from the manuscript (include quotes in quotation marks "like this" to indicate direct quotes from your manuscript), or elaborate on this item by providing additional information not in the ms, or briefly explain why the item is not applicable/relevant for your study

"Treatment strategies were not blinded to patients or recruiters, since the therapy strategies cannot be effectively hidden from either. Blinding created through a placebo app was not implemented, since BfArM guidelines for the evaluation of DHAs require evidence of superiority over usual care (SHI-PT in Germany), which does not include any additional DHA. Data collection was performed using web-based electronic questionnaires with only participants themselves being involved in generating the data. We can therefore rule out virtually any bias on our results created by the lack of blinding in our study."

21) Generalisability (external validity, applicability) of the trial findings

NPT: External validity of the trial findings according to the intervention, comparators, patients, and care providers or centers involved in the trial

21-i) Generalizability to other populations

Generalizability to other populations: In particular, discuss generalizability to a general Internet population, outside of a RCT setting, and general patient population, including applicability of the study results for other organizations

1 2 3 4 5

Ihre Antwort ist zu lang. Kürzen Sie einige Sätze in Ihrer Antwort.

Does your paper address subitem 21-i?

Copy and paste relevant sections from the manuscript (include quotes in quotation marks "like this" to indicate direct quotes from your manuscript), or elaborate on this item by providing additional information not in the ms, or briefly explain why the item is not applicable/relevant for your study

"Our findings indicate that the investigated DHA is superior to SHI-PT for treating disorders of the patella. Sex and age did not have any effect on the outcomes of the study. In addition, post treatment pain medication intake was substantially lower for DHA compared to SHI-PT. DHA has therefore been approved by BfArM for persons of all sexes aged 12 and over for the treatment of disorders of the patella."

21-ii) Discuss if there were elements in the RCT that would be different in a routine application setting

Discuss if there were elements in the RCT that would be different in a routine application setting (e.g., prompts/reminders, more human involvement, training sessions or other co-interventions) and what impact the omission of these elements could have on use, adoption, or outcomes if the intervention is applied outside of a RCT setting.

	1	2	3	4	5	
subitem not at all important	○	○	○	○	○	essential

Does your paper address subitem 21-ii?

Copy and paste relevant sections from the manuscript (include quotes in quotation marks "like this" to indicate direct quotes from your manuscript), or elaborate on this item by providing additional information not in the ms, or briefly explain why the item is not applicable/relevant for your study

Not applicable to our study.

OTHER INFORMATION

Ihre Antwort ist zu lang. Kürzen Sie einige Sätze in Ihrer Antwort.

Does your paper address CONSORT subitem 23? *

Copy and paste relevant sections from the manuscript (include quotes in quotation marks "like this" to indicate direct quotes from your manuscript), or elaborate on this item by providing additional information not in the ms, or briefly explain why the item is not applicable/relevant for your study

"The prespecified study protocol was approved and registered by the Institutional Ethics Committee of the Faculty of Behavioural and Social Sciences of the Chemnitz University of Technology (registration number: V-439-17-CM-MAWENDO-II-18042021) in agreement with current data protection regulations. The trial was registered with the DRKS (German Clinical Trials Register; WHO Primary Register) under No. DRKS00023454 for 14 different orthopedic ICD-10 indications. These 14 ICD-10 indications will be examined in 11 separate randomized controlled prospective clinical trials (RCT) with two treatment groups. The present RCT is the first among the 11 RCTs to have completed treating the pre-specified number of patients. At the time of writing this manuscript, the 10 remaining RCTs are still in the stage of data collection."

24) Where the full trial protocol can be accessed, if available

Does your paper address CONSORT subitem 24? *

Cite a Multimedia Appendix, other reference, or copy and paste relevant sections from the manuscript (include quotes in quotation marks "like this" to indicate direct quotes from your manuscript), or elaborate on this item by providing additional information not in the ms, or briefly explain why the item is not applicable/relevant for your study

Not applicable to our study.

25) Sources of funding and other support (such as supply of drugs), role of funders

Ihre Antwort ist zu lang. Kürzen Sie einige Sätze in Ihrer Antwort.

Does your paper address CONSORT subitem 25? *

Copy and paste relevant sections from the manuscript (include quotes in quotation marks "like this" to indicate direct quotes from your manuscript), or elaborate on this item by providing additional information not in the ms, or briefly explain why the item is not applicable/relevant for your study

"The study was sponsored by Mawendo (Mawendo GmbH, Biberach, Germany). Mawendo provided access to DHA for all participants. The sponsor of the study had no role in designing or conducting the study, analyzing or interpreting data, or in writing the manuscript. The trial was designed and conducted by independent academic investigators from the Professorship for Research Methodology and Data Analysis in Biomechanics at the Chemnitz University of Technology."

X27) Conflicts of Interest (not a CONSORT item)

X27-i) State the relation of the study team towards the system being evaluated

In addition to the usual declaration of interests (financial or otherwise), also state the relation of the study team towards the system being evaluated, i.e., state if the authors/evaluators are distinct from or identical with the developers/sponsors of the intervention.

	1	2	3	4	5	
subitem not at all important	○	○	○	○	○	essential

Does your paper address subitem X27-i?

Copy and paste relevant sections from the manuscript (include quotes in quotation marks "like this" to indicate direct quotes from your manuscript), or elaborate on this item by providing additional information not in the ms, or briefly explain why the item is not applicable/relevant for your study

"The authors declare no conflicts of interests."

About the CONSORT EHEALTH checklist

Ihre Antwort ist zu lang. Kürzen Sie einige Sätze in Ihrer Antwort.

As a result of using this checklist, did you make changes in your manuscript? *

- ○ yes, major changes
- ◉ yes, minor changes
- ○ no

What were the most important changes you made as a result of using this checklist?

More details of the randomization process.

How much time did you spend on going through the checklist INCLUDING making changes in your manuscript *

three hours................

As a result of using this checklist, do you think your manuscript has improved? *

- ○ yes
- ◉ no
- ○ Sonstiges:

Ihre Antwort ist zu lang. Kürzen Sie einige Sätze in Ihrer Antwort.

Would you like to become involved in the CONSORT EHEALTH group?

This would involve for example becoming involved in participating in a workshop and writing an "Explanation and Elaboration" document

- ○ yes
- ◉ no
- ○ Sonstiges:

Auswahl löschen

Any other comments or questions on CONSORT EHEALTH

Meine Antwort

STOP - Save this form as PDF before you click submit

To generate a record that you filled in this form, we recommend to generate a PDF of this page (on a Mac, simply select "print" and then select "print as PDF") before you submit it.

When you submit your (revised) paper to JMIR, please upload the PDF as supplementary file.

Don't worry if some text in the textboxes is cut off, as we still have the complete information in our database. Thank you!

Final step: Click submit !

Click submit so we have your answers in our database!

Senden

Alle Eingaben löschen

Geben Sie niemals Passwörter über Google Formulare weiter.

Dieser Inhalt wurde nicht von Google erstellt und wird von Google auch nicht unterstützt. Missbrauch melden - Nutzungsbedingungen - Datenschutzerklärung

Google Formulare

Ihre Antwort ist zu lang. Kürzen Sie einige Sätze in Ihrer Antwort.
